# Supplementary material for: Novel AAV variants with improved tropism for human Schwann cells
Source: Mol Ther Methods Clin Dev. 2024 Mar 11;32(2):101234. doi: 10.1016/j.omtm.2024.101234 (PMC10978538; doi:10.1016/j.omtm.2024.101234)
Supplement: Document S1. Figures S1–S12 and Tables S1–S3 [file mmc1.pdf]

OMTM, Volume *32*

Supplemental information

Novel AAV variants with improved tropism for human Schwann cells

Matthieu Drouyer, Tak-Ho Chu, Elodie Labit, Florencia Haase, Renina Gale Navarro, Deborah Nazareth, Nicole Rosin, Jessica Merjane, Suzanne Scott, Marti Cabanes-Creus, Adrian Westhaus, Erhua Zhu, Rajiv Midha, Ian E. Alexander, Jeff Biernaskie, Samantha L. Ginn, and Leszek Lisowski

Supplemental material

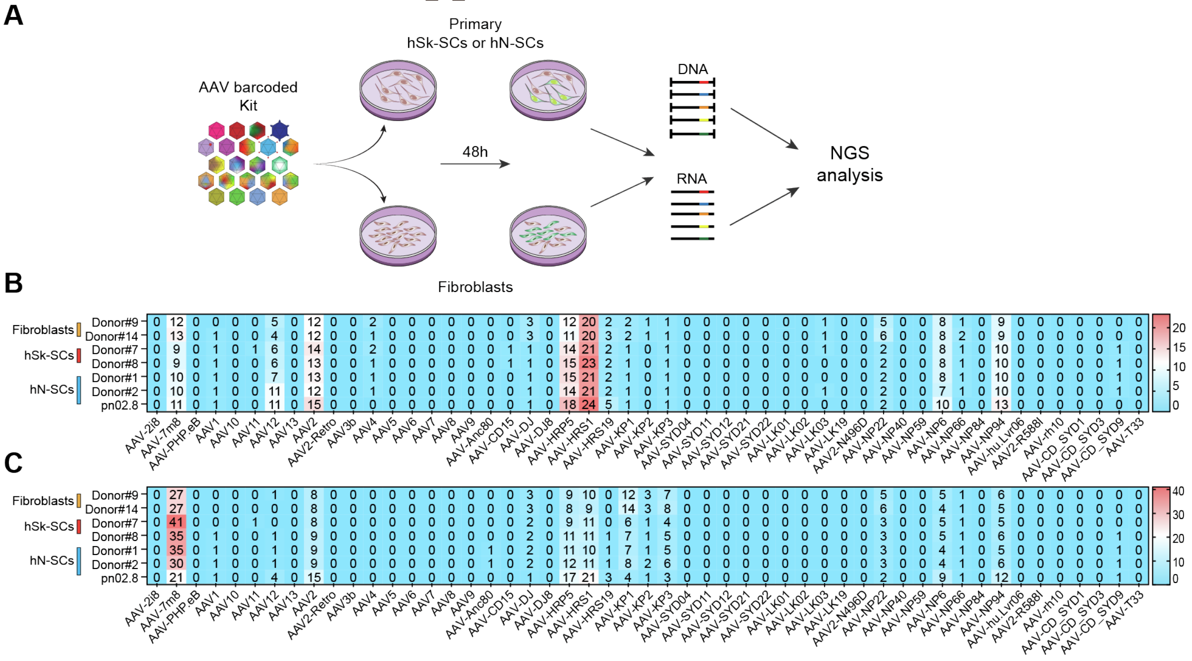

Figure S1. Performance of pooled AAV variants in primary hSCs and in fibroblasts. (A) Graphical overview of the AAV testing kit process *in vitro.* (B and C) The use of the pooled 51-variant "AAV Testing Kit" allows for simultaneous analysis of barcoded variants with capsid recovery achieved at the level of both (B) cell entry (DNA) and (C) transgene expression (mRNA). Heat-map indicates the performance of each capsid as a percentage of total NGS reads per cell type. Values are normalized to the 'pre-mix pool'.

DNA_human SCs

Donor#11	Donor#12	Donor#13	Donor#14	Donor#4	Donor#3	pn02.8	Variant	Average
11.860	8.610	9.000	7.260	8.200	8.770	2.964	Pep2hSC2	8.095
11.420	7.790	7.040	6.390	6.870	7.390	4.216	Pep2hSC1	7.302
5.950	4.410	4.110	4.710	5.630	5.040	4.056	AAV-HRP5	4.844
5.070	4.430	4.140	4.350	4.880	4.840	3.749	AAV2	4.494
4.500	4.270	3.990	4.200	4.810	4.230	3.431	AAV-NP6	4.204
4.530	4.630	3.800	4.160	4.600	4.200	3.470	Shuffle35	4.199
4.360	4.040	3.940	4.100	4.530	4.230	3.400	AAV-NP94	4.086
4.420	4.150	3.790	3.950	4.430	4.160	3.447	AAV-HRS1	4.050
4.420	3.900	3.970	3.460	3.500	3.810	3.790	Pep9hSC5	3.836
4.040	3.910	3.710	3.780	3.960	3.920	3.302	Shuffle9	3.803
1.630	2.880	3.010	3.700	6.950	2.940	3.711	Pep9hSC2	3.546
2.810	3.720	4.050	3.850	3.930	3.590	2.093	AAV-7m8	3.435
3.040	3.210	3.130	3.180	3.740	3.210	2.666	Shuffle32	3.168
2.660	3.190	3.450	3.080	2.160	3.240	3.972	Pep9hSC9	3.107
1.710	2.390	2.490	2.700	3.810	2.350	3.621	Pep9hSC1	2.724
3.730	2.350	2.440	2.210	2.550	2.460	3.190	PephSC10	2.704
2.710	2.870	2.700	2.830	1.520	2.680	3.031	Shuffle31	2.620
2.720	2.700	2.880	2.520	1.340	2.510	2.894	Pep2hSC5	2.509
1.780	2.340	2.420	2.150	3.330	2.120	2.444	Pep2hSC4	2.369
1.390	2.380	2.400	2.680	1.670	2.400	3.643	Pep9hSC10	2.366
1.370	2.080	2.240	3.180	0.780	2.510	3.478	Pep9hSC8	2.234
1.270	2.040	2.070	2.480	0.690	2.230	3.498	Pep9hSC7	2.040
1.120	1.740	2.050	1.520	4.030	1.370	1.175	AAV-DJ	1.858
1.870	2.020	2.290	1.930	0.410	2.340	2.137	Pep2hSC9	1.857
1.780	1.690	1.730	1.740	0.980	1.770	1.394	AAV-KP3	1.583
0.630	1.010	1.140	1.850	2.530	1.150	2.605	Pep9hSC6	1.559
1.260	1.730	2.000	1.240	1.130	1.520	1.641	PephSC8	1.503
0.660	1.370	1.360	1.930	0.840	1.450	2.845	Pep9hSC11	1.494
0.890	1.460	1.490	1.110	1.030	1.250	1.914	Pep2hSC7	1.306
1.170	1.510	1.460	1.150	0.630	0.960	2.227	Pep2hSC3	1.301
0.850	1.310	1.440	1.220	1.020	1.130	1.847	Pep2hSC6	1.260
0.510	0.980	0.920	1.590	0.690	1.040	2.644	Pep9hSC12	1.196
0.480	0.880	1.050	1.500	0.230	1.060	2.625	Pep9hSC3	1.118
0.630	0.820	1.030	0.790	0.590	0.640	0.850	AAV-NP22	0.764
0.350	0.570	0.540	1.010	0.680	0.780	1.339	Pep9hSC4	0.753
0.410	0.640	0.730	0.510	1.320	0.700	0.693	AAV-KP1	0.715

cDNA_human SCs

Donor#11	Donor#12	Donor#13	Donor#14	Donor#4	Donor#3	pn02.8	Variant	Average
18.250	11.050	7.510	19.040	15.290	20.210	9.615	Pep2hSC1	14.424
8.420	20.330	18.660	2.260	13.450	6.720	4.816	AAV-7m8	10.665
10.010	3.200	1.800	18.120	5.800	5.150	1.521	Pep2hSC2	6.514
5.590	5.500	4.970	3.120	6.150	4.690	4.827	Shuffle35	4.978
4.370	4.350	3.880	7.850	5.860	3.700	4.531	Shuffle32	4.934
3.510	8.460	11.290	1.120	4.830	2.470	1.211	AAV-KP1	4.699
3.890	3.040	3.090	7.150	3.900	3.720	4.263	Shuffle9	4.150
2.920	4.980	5.830	0.660	4.140	2.980	1.949	AAV-KP3	3.351
3.040	3.760	3.640	0.660	4.210	3.580	4.499	Shuffle31	3.341
4.280	2.440	2.340	0.830	2.950	3.770	4.766	AAV-HRP5	3.054
3.280	2.440	3.210	0.620	2.750	3.180	5.255	AAV2	2.962
3.160	2.310	3.130	1.500	2.910	3.050	3.853	AAV-NP6	2.845
3.410	2.120	1.730	1.740	2.980	4.170	2.611	Pep9hSC5	2.680
2.930	1.720	2.420	1.490	2.370	2.770	4.428	AAV-NP94	2.590
2.360	1.810	1.350	3.990	2.200	3.210	2.845	Pep9hSC9	2.538
2.800	1.680	2.530	0.760	2.430	2.910	4.643	AAV-HRS1	2.536
1.770	3.080	4.110	1.070	2.140	1.040	1.272	AAV-DJ	2.069
2.100	2.230	2.770	0.520	1.650	1.600	2.512	Pep2hSC5	1.912
1.280	1.530	1.460	3.080	1.420	2.330	2.026	Pep2hSC4	1.875
0.990	1.640	1.420	4.350	1.630	1.510	0.999	Pep2hSC8	1.791
1.160	1.040	0.950	1.430	0.990	2.040	3.235	Pep9hSC1	1.549
0.530	0.740	0.700	3.130	0.630	1.590	2.284	Pep9hSC11	1.372
0.350	0.540	0.510	0.850	0.500	1.200	2.280	Pep2hSC6	1.335
0.910	0.870	0.810	1.210	0.860	1.650	2.674	Pep9hSC2	1.283
1.130	1.760	1.440	0.640	0.950	0.800	2.055	Pep2hSC3	1.254
0.830	0.900	1.310	1.200	1.170	0.950	1.884	Pep2hSC7	1.178
0.610	0.750	0.710	1.100	0.860	1.480	2.025	Pep9hSC7	1.076
0.280	0.250	0.220	4.440	0.270	0.440	1.056	Pep9hSC3	0.994
0.840	1.620	2.260	0.010	0.910	0.530	0.773	AAV-NP22	0.992
0.580	0.600	0.530	1.290	0.560	1.250	1.571	Pep9hSC8	0.912
1.610	0.350	0.290	1.080	0.760	0.740	1.443	PephSC10	0.896
0.940	1.320	1.660	1.460	0.880	1.350	1.737	Pep9hSC6	0.890
0.680	0.460	0.390	1.090	0.650	0.900	0.849	Pep9hSC10	0.717
0.700	0.560	0.560	0.220	0.510	0.890	1.491	Pep2hSC9	0.704
0.290	0.360	0.300	0.580	0.310	0.760	1.391	Pep9hSC12	0.570
0.190	0.220	0.230	0.370	0.150	0.670	0.808	Pep9hSC4	0.377

DNA_Fibroblasts

F-Donor#10	F-Donor#6	F-Donor#5	Variant	Average
5.830	6.120	6.380	AAV2	6.110
5.640	5.710	5.720	AAV-7m8	5.690
5.400	5.770	5.820	AAV-NP94	5.663
5.200	5.610	5.880	Shuffle35	5.563
5.280	5.520	5.850	AAV-NP6	5.550
6.180	5.390	4.550	Pep2hSC5	5.373
4.940	5.340	5.660	Shuffle9	5.313
4.900	5.370	5.560	AAV-HRS1	5.277
4.390	4.530	4.750	AAV-HRP5	4.557
5.380	4.830	2.940	AAV-DJ	4.383
4.100	4.340	4.460	Shuffle31	4.300
3.890	4.160	4.620	Shuffle32	4.223
3.900	3.870	3.520	Pep2hSC4	3.763
3.390	3.590	3.820	Pep2hSC10	3.600
3.480	3.250	2.920	Pep2hSC9	3.217
3.600	2.710	2.440	Pep2hSC8	2.917
2.330	2.600	3.630	Pep2hSC1	2.853
2.710	2.600	1.960	Pep2hSC7	2.423
2.510	2.430	2.240	AAV-KP3	2.393
1.930	2.120	3.050	Pep2hSC2	2.367
2.400	2.160	2.140	Pep2hSC3	2.233
2.680	2.070	1.220	AAV-NP22	1.990
2.360	1.660	1.010	AAV-KP1	1.677
1.800	1.690	1.200	Pep2hSC6	1.563
1.180	1.170	1.640	Pep9hSC5	1.330
0.920	1.040	1.250	Pep9hSC9	1.070
0.540	0.670	0.930	Pep9hSC2	0.713
0.500	0.680	0.820	Pep9hSC1	0.667
0.530	0.560	0.770	Pep9hSC10	0.620
0.500	0.580	0.720	Pep9hSC8	0.600
0.430	0.510	0.700	Pep9hSC7	0.547
0.260	0.310	0.420	Pep9hSC11	0.330
0.230	0.320	0.440	Pep9hSC12	0.330
0.310	0.310	0.360	Pep9hSC6	0.327
0.230	0.250	0.340	Pep9hSC3	0.273
0.120	0.160	0.240	Pep9hSC4	0.173

cDNA_Fibroblasts

F-Donor#10	F-Donor#6	F-Donor#5	Variant	Average
23.200	17.700	10.630	AAV-KP1	17.177
13.340	13.740	20.100	AAV-7m8	15.727
7.650	6.840	6.570	AAV-KP3	7.020
8.040	6.800	4.890	AAV-DJ	6.577
5.400	5.970	7.650	Shuffle32	6.340
4.970	5.900	7.150	Shuffle35	6.007
1.830	4.280	5.700	Shuffle31	3.937
3.280	3.590	4.460	Shuffle9	3.777
2.630	3.080	4.610	AAV2	3.440
2.700	3.320	3.940	AAV-HRP5	3.320
2.370	3.020	4.430	AAV-NP6	3.273
2.160	2.610	3.820	AAV-HRS1	2.863
2.100	2.620	3.630	AAV-NP94	2.783
2.930	2.620	2.250	Pep2hSC5	2.600
3.120	3.770	0.660	Pep2hSC1	2.517
2.830	2.530	1.820	AAV-NP22	2.393
1.830	1.510	1.370	Pep2hSC7	1.570
1.870	1.450	1.240	Pep2hSC8	1.520
1.300	1.390	1.130	Pep2hSC6	1.273
1.000	0.930	1.140	Pep2hSC3	1.023
0.970	1.030	1.010	Pep2hSC4	1.003
1.120	1.330	0.210	Pep2hSC2	0.887
0.640	0.900	0.170	Pep9hSC5	0.570
0.500	0.550	0.530	Pep2hSC9	0.527
0.450	0.590	0.110	Pep9hSC9	0.383
0.320	0.350	0.140	Pep2hSC10	0.270
0.290	0.330	0.140	Pep9hSC1	0.253
0.220	0.250	0.100	Pep9hSC2	0.190
0.160	0.170	0.060	Pep9hSC6	0.130
0.150	0.160	0.070	Pep9hSC7	0.127
0.150	0.130	0.050	Pep9hSC11	0.110
0.130	0.150	0.050	Pep9hSC8	0.110
0.110	0.140	0.040	Pep9hSC10	0.097
0.090	0.100	0.030	Pep9hSC12	0.073
0.080	0.090	0.050	Pep9hSC3	0.073
0.050	0.060	0.030	Pep9hSC4	0.047

Highest

Lowest

Figure S2. Performance of selected AAV variants in primary hSCs and in fibroblasts. Heat maps show the performance of selected variants. Percentage of NGS reads mapped to each AAV capsid (average of n = 2 barcodes/capsid) in seven primary SCs cultures and in three fibroblast cultures at both the level of DNA (cell entry, physical transduction) and mRNA (expression, functional transduction). Data is normalized to the 'pre-mix pool'.

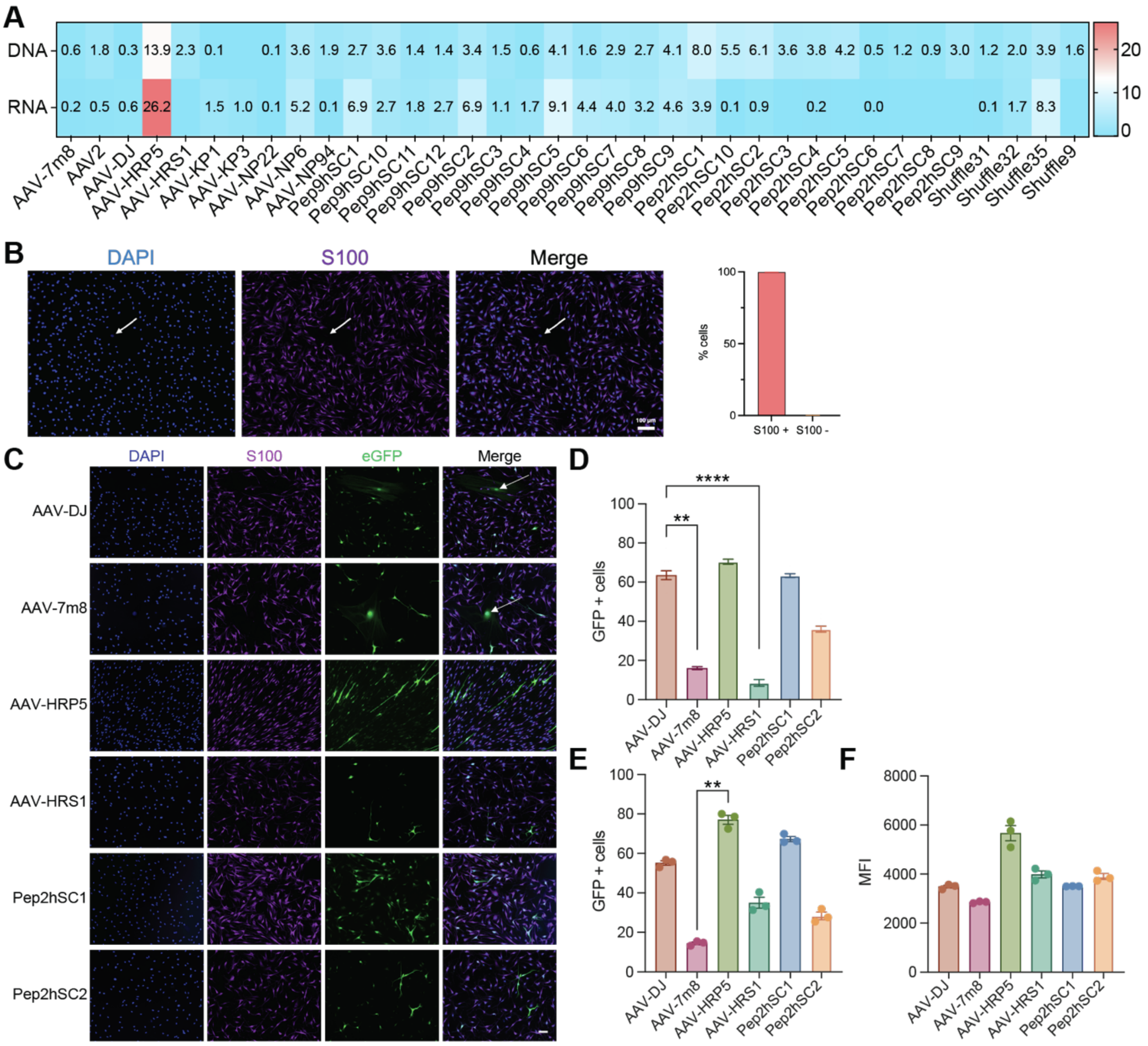

Figure S3. Assessment of AAV transduction on rat SCs.

(A) Analysis of barcoded variants with capsid recovery achieved at the level of both cell entry (DNA) and transgene expression (mRNA). Heat-map indicates the performance of each capsid as a percentage of total NGS reads for each cell type. Values are normalized to 'pre-mix pool' and are the average of 2 barcodes. (B and C) Rat SCs were transduced with the indicated variants (1,000 vg/cell) and analyzed by flow cytometry. (D) Characterization of the primary rat SC cultures derived from rat nerve segment. Representative images of rat and SC cultures stained with DAPI (blue) and the SCs marker S100 (purple). Quantification of the S100+ and S100- cells in three independent cultures. (E) Representative images of pure cultured rat SCs transduced with eGFP reporter AAVs packaged using indicated capsids (1,000 vg/cell). Blue: DAPI, purple: S100 (SCs marker), green: AAV-encoded GFP. Scale bar: 50 μm. (F) Percentage of eGFP+/S100+ cells. Quantification was performed from ≥ 10 images per variants with ≥ 80 cells counted per image. P values were determined by one-way ANOVA non-parametric Kruskal-Wallis test with Dunn's multiple comparison test (**p≤0.01; ****p≤0.0001). Data is represented as mean ± SEM.

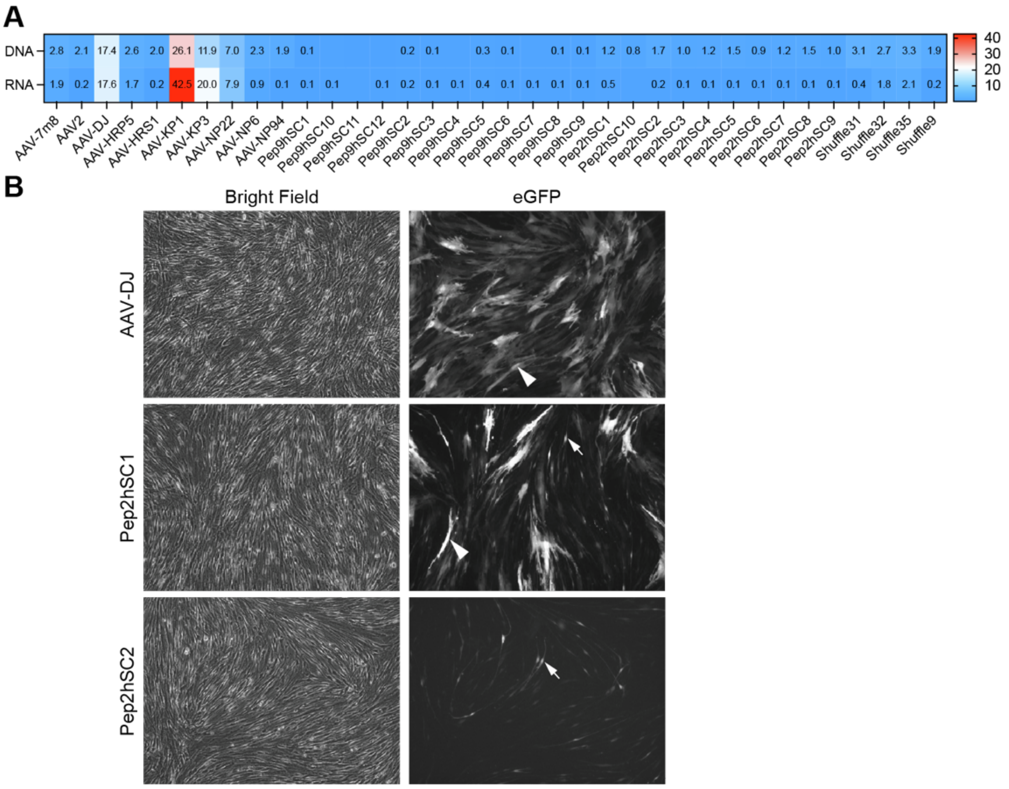

Figure S4. Assessment of AAV transduction on mouse SCs.
(A) Analysis of barcoded variants with capsid recovery achieved at the level of both cell entry (DNA) and transgene expression (mRNA). Heat-map indicates the performance of each capsid as a percentage of total NGS reads for each cell type. Values were normalized to 'pre-mix pool' and are the average of 2 barcodes. (B) Bright-field and fluorescence images 48 hours after AAV-GFP transduction of mouse SCs. Examples of transduced fibroblasts (arrowheads) and mouse SCs (arrows) are shown.

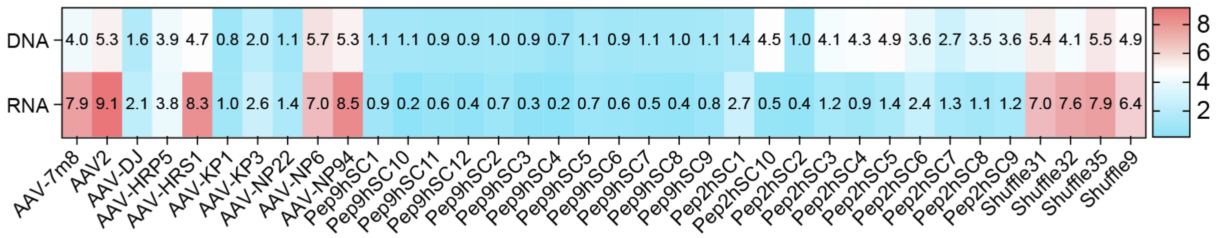

Figure S5. Assessment of AAV transduction on immortalized hSCs.

Analysis of barcoded variants with capsid recovery achieved at the level of both cell entry (DNA) and transgene expression (mRNA). Heat-map indicates the performance of each capsid as a percentage of total NGS reads from two transductions. Values were normalized to the 'pre-mix pool' and are the average of 2 barcodes.

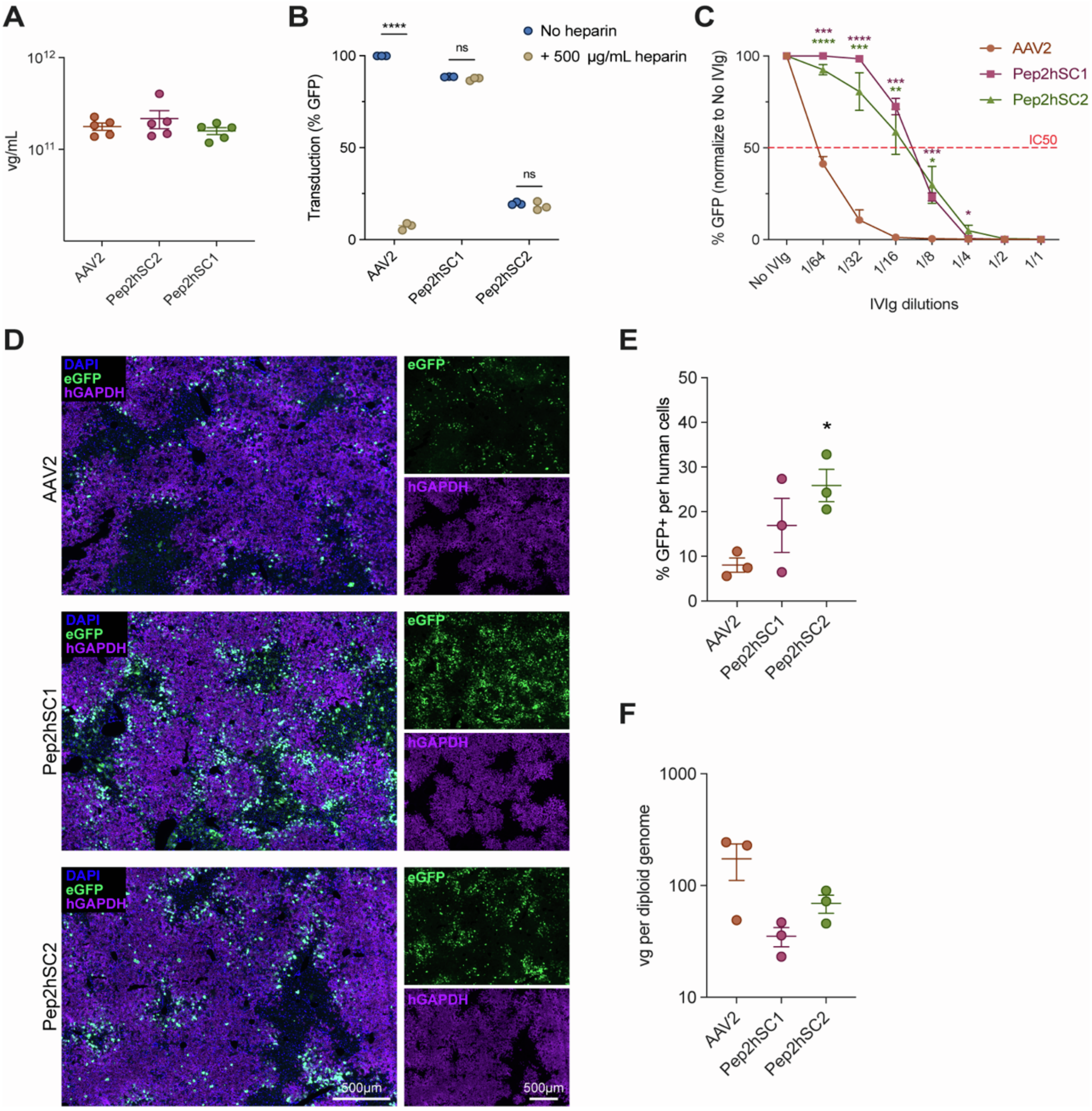

Figure S6. Characterisation of novel AAV capsid variants.
(A) Comparison of recombinant AAV crude production. Data are presented as mean ± SEM (n = 5 independent single dish lysate). (B) Heparin competition assay. AAV vector variants and the parental AAV2 were pre-incubated with heparin or not. Transduction efficiency on HEK293T cells was quantified by FACS according to eGFP positive cells 72h after transduction (n = 3 independent experiments). Data are represented as means ± SEM. Statistical significance was calculated using the two-tailed Mann-Whitney test by comparing vector performance with and without the presence of soluble heparin (****$p \leq 0.0001$; n.s., $p > 0.05$). (C) Neutralization assay of indicated AAV vectors following pre-incubation with human IVIg prior to transduction of HEK293T cells. The percentage of eGFP-positive cells 72h after transduction was analyzed by flow cytometry (n = 4 independent experiments). The dotted red line represents IVIg-mediated inhibition of AAV transduction by 50%. P values were determined by ordinary two-way ANOVA using Śidák's multiple comparison test (*$p \leq 0.05$; **$p \leq 0.01$, ***$p \leq 0.001$; ****$p \leq 0.0001$). Data is shown as mean ± SEM. (D-F) Evaluation of novel AAV variants in the humanized FRG (hFRG) model. (D) Representative images of hFRG mice liver tissue following I.V. injection with either AAV-DJ, Pep2hSC1 or Pep2hSC2 (dose = 2×10^{11} vg; n = 3 per variant). Human hepatocytes (purple), vector-expressed eGFP (green), DAPI (blue). Scale bar: 500µm. (E) Quantification of the percentage of transduced

human hepatocytes, individual data points represent the average of 11-15 human clusters per mouse. (F) AAV vector genomes per diploid cell in human hepatocyte cells. (F) Quantification of (D) the percentage of transduced human hepatocytes, Individual data points represent the average of 11-15 human clusters per mouse. Data are represented as mean ± SEM.

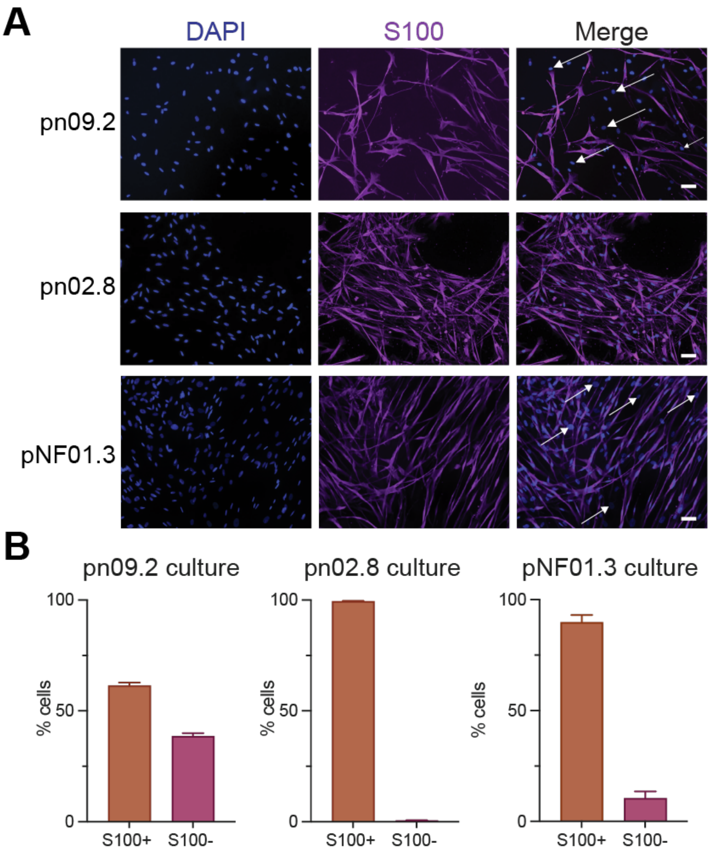

Figure S7. Characterisation of the primary human SC cultures.
(A) Representative images of human pn09.2, pn02.8 and pNF01.3 SC cultures stained with DAPI (blue) and the SCs marker S100 (purple). Arrows indicate cells that are S100-. Scale bar, 50µm. (B) Quantification of the S100+ and S100- cells in three independent cultures shows that pn09.2 culture contained both population with on average 60% of S100 positive and 40% of S100 negative cells, pNF03.1 culture contained both population with on average 90% of S100 positive and 10% of S100 negative cells while pn02.8 culture contained only S100 positive cells.

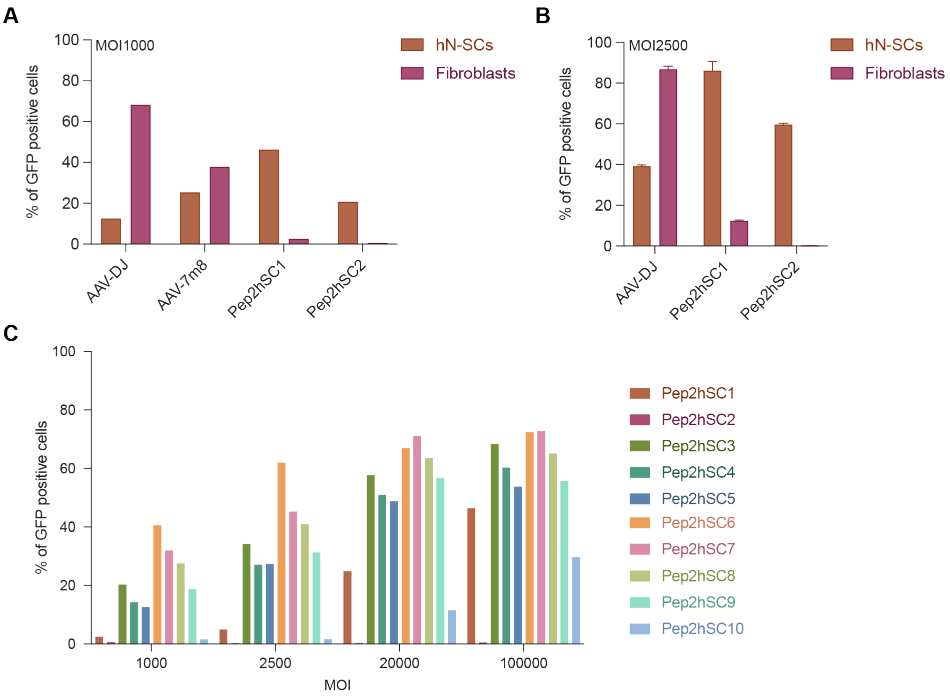

Figure S8. Transduction efficiency of selected variants for primary human SCs and fibroblasts.

(A-C) hN-SCs or fibroblast cells were transduced with vectors encoding a CMV-eGFP transgene at the doses indicated. 3 days post transduction cells were analysed by flow cytometry according to eGFP expression.

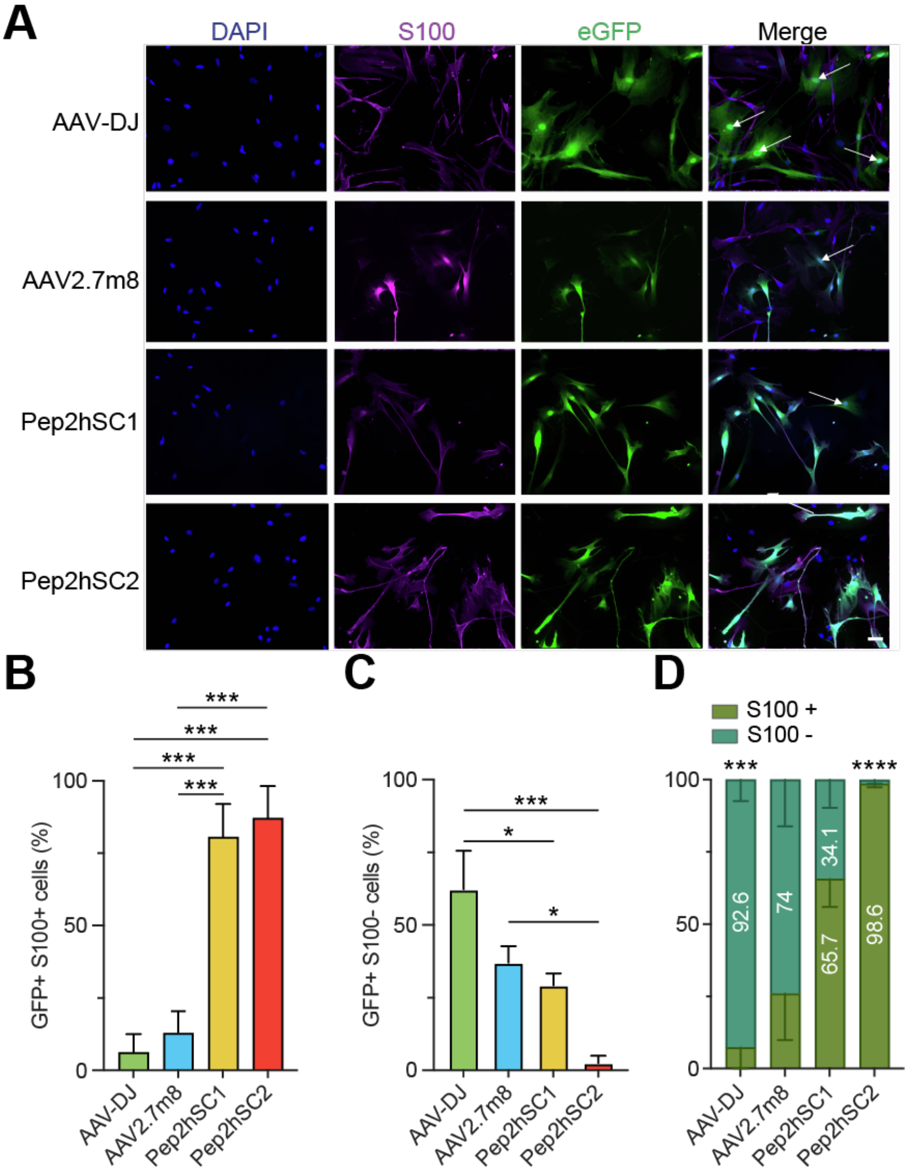

Figure S9. Transduction of pn09.2 SC culture with top AAV variants at MOT 10,000. (A) Representative immunofluorescence images of mixed cultured hSCs transduced with indicated AAV variants encoding an eGFP reporter (10,000 vg/cell). Arrows indicate eGFP+/S100- cells. Scale bar, 50µm. (B and C) Percentage of (B) eGFP+/S100+ cells, (C) eGFP+/S100- cells. Quantification was performed using ≥ 30 cells per image, and ≥ 4 images per variant. P values were determined by one-way ANOVA with Holm-Śidák's multiple comparison test (*p≤0.05; ***p≤0.001). Data are given as mean ± SEM. (D) Proportion of eGFP+ cells in the mixed hSC culture. Percentages of S100+ and S100- cells among total eGFP+ cells were calculated. P values were determined by unpaired t-test (***p≤0.001; ****p≤0.0001).

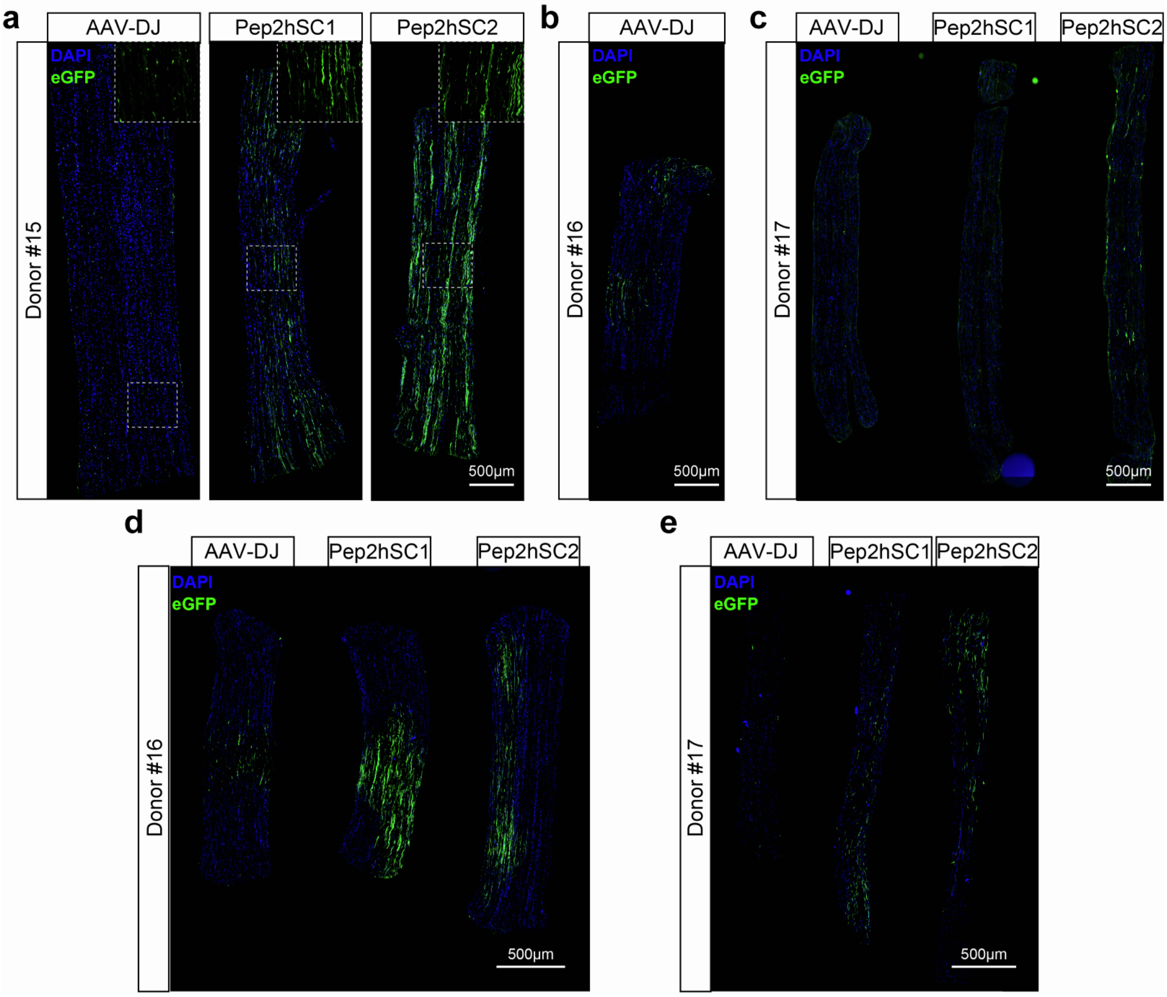

Figure S10. eGFP expression in human nerve segments.
Immunofluorescence of longitudinal sections of human sural nerve segments at (A-C) 7 days post injection and (D and E) 14 days post injection. Nerve segments (0.5cm) were injected with AAV-DJ, Pep2hSC1 or Pep2hSC2 vectors encoding a CMV-eGFP transgene (1×10^{10} vg dose per segment). DAPI (blue), AAV-encoded eGFP (green). Scale bar: 500µm.
Donor#15: 42-year-old male; Donor#16: 44-year-old male, Caucasian; Donor#17: 73-year-old male, Asian.

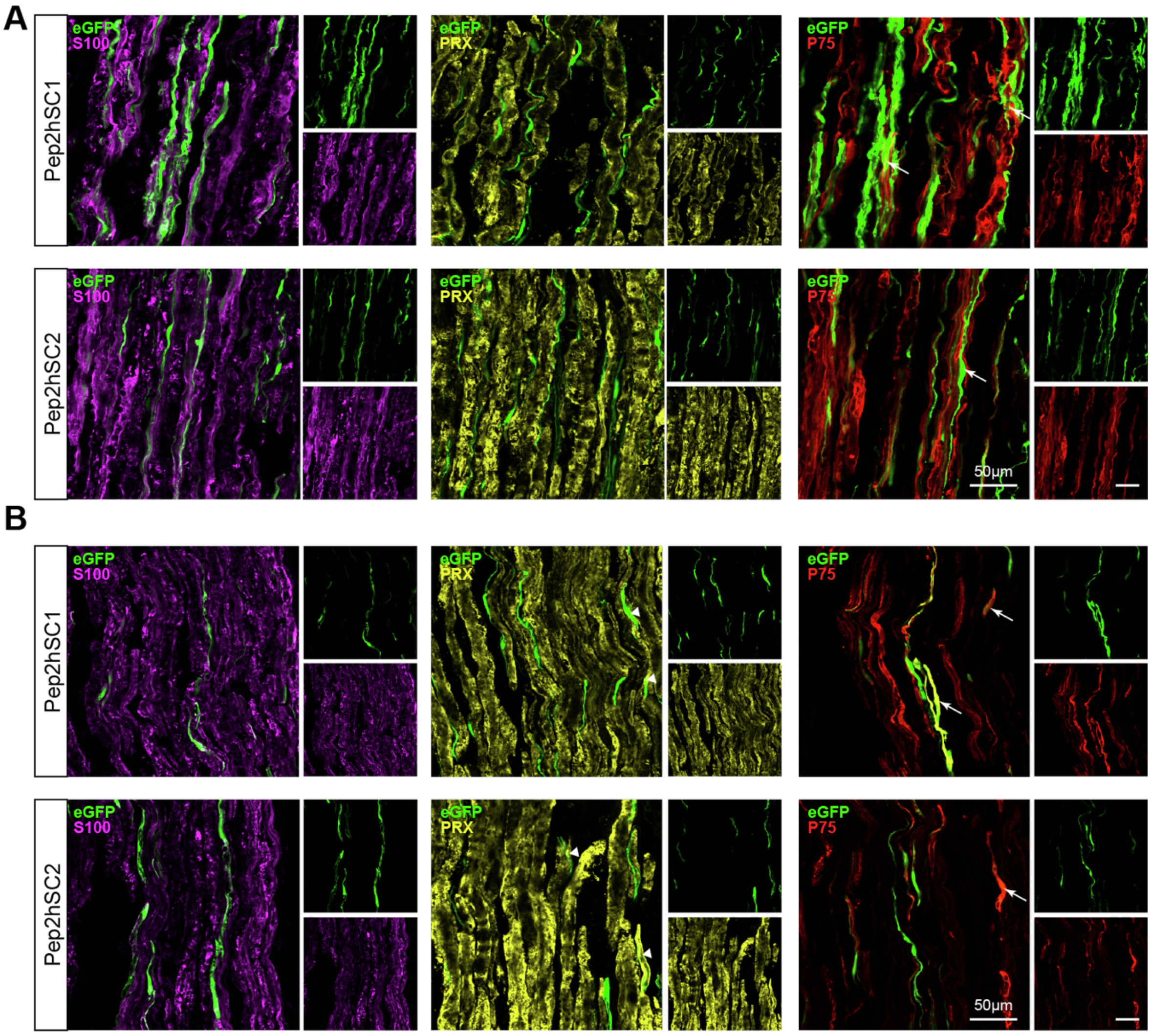

Figure S11. Immunolabeling for myelinating and non-myelinating SCs.
(A and B) Confocal microscopy images showing double immunostaining for eGFP and various markers on longitudinal sections (A) for Donor#16 and (B) for Donor#17. The sections were stained with either S100 (purple) for SCs, PRX (yellow) for myelinating SCs, or P75 (red) for non-myelinating SCs. Arrowheads in the right panel images show colocalization with non-myelinating SC marker P75. Donor#17 had some eGFP+ and PRX positive cells (arrows). Scale bar: 50µm.
Donor#16: 44-year-old male, Caucasian; Donor#17: 73-year-old male, Asian.

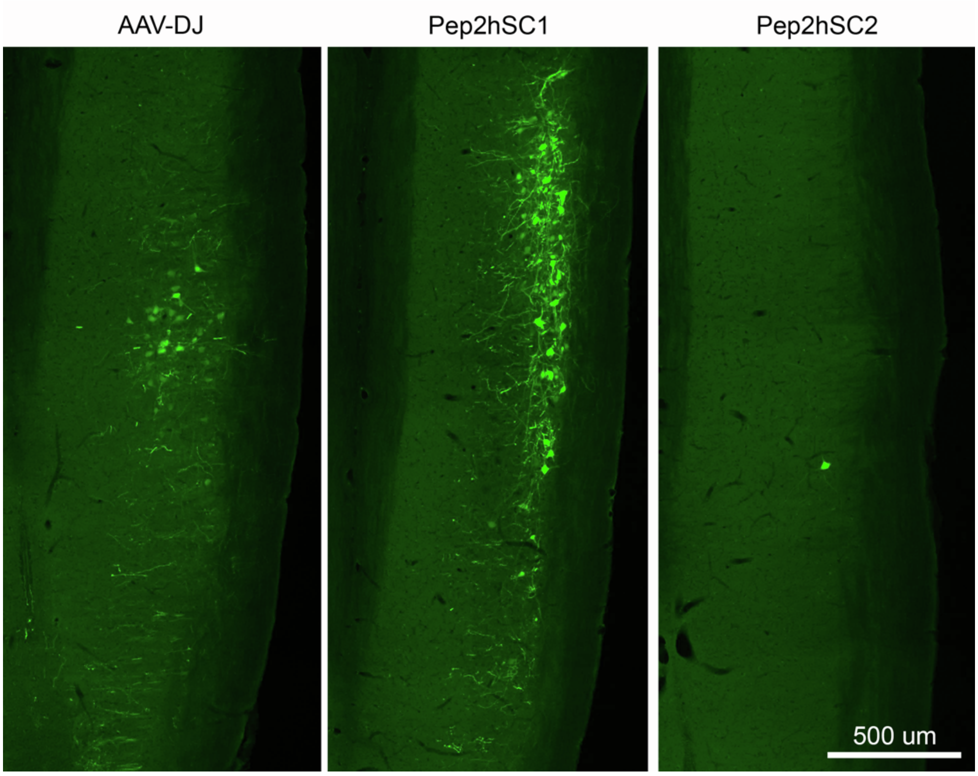

Figure S12. eGFP expression in spinal cord of mice administered by intraneural injection. Representative images of longitudinal spinal cord sections of mice following intraneural injection with either AAV-DJ, Pep2hSC1 or Pep2hSC2 (dose = 2×10^{10} vg; n = 1 per variant). Scale bar: 500µm.

Table S1. Information of donors used in studies conducted in primary hSC and fibroblast cultures and with *ex vivo* nerve explants.

Cell/Donor ID	Skin/Nerve/Fibroblast	Race/ethnicity	Age	Sex	Sources
pn02.8	Nerve	/	55	M	/
pNF01.3	Nerve tumor	/	/	/	Biopsy
pn09.2	Nerve	/	/	/	/
Donor#1	Nerve	Caucasian	28	M	Autopsy
Donor#2	Nerve	Asian	47	M	Autopsy
Donor#3	Nerve	Caucasian	58	M	Autopsy
Donor#4	Nerve	Caucasian	62	M	Autopsy
Donor#5	Fibroblast	Caucasian	50	F	Biopsy
Donor#6	Fibroblast	Caucasian	60	M	Biopsy
Donor#7	Skin	Caucasian	51	F	Biopsy
Donor#8	Skin	Caucasian	56	F	Biopsy
Donor#9	Fibroblast	Caucasian	63	M	Autopsy
Donor#10	Fibroblast	Caucasian	48	F	Biopsy
Donor#11	Skin	Caucasian	26	M*	Biopsy
Donor#12	Skin	Caucasian	64	F	Biopsy
Donor#13	Skin	Caucasian	49	F	Biopsy
Donor#14	Nerve	Caucasian	66	M	Autopsy
Donor#15	Nerve	/	42	M	Autopsy
Donor#16	Nerve	Caucasian	44	M	Autopsy
Donor#17	Nerve	Asian	73	M	Autopsy

Table S2. Information of primary human hepatocyte donors used to engraft FRG mice.

Sex	Internal ID	Albumin level [mg/ml]	Experiment	Cell origin	Replacement Index
Female	311	16.207	AAV2_2e11vg	Lonza #HUM181971	90.7%
Female	323	14.188	AAV2_2e11vg	Lonza #HUM181971	85.5%
Female	70	16.446	AAV2_2e11vg	Lonza #HUM181971	81.0%
Female	548	10.506	Pep2hSC1_2e11vg	Lonza #HUM181141	78.4%
Female	392	15.481	Pep2hSC1_2e11vg	Lonza #HUM181141	88.6%
Female	434	14.224	Pep2hSC1_2e11vg	Lonza #HUM181971	84.2%
Female	589	13.72	Pep2hSC2_2e11vg	Lonza #HUM181141	37.3%
Female	438	17.057	Pep2hSC2_2e11vg	Lonza #HUM181971	81.6%
Female	469	15.331	Pep2hSC2_2e11vg	Lonza #HUM181971	89.1%

Table S3. Oligonucleotide sequences.

Primer Name	Sequence (5'->3')
lco9_NNK7	CCATCCGGTTTGGGCCGCCTBMNNMNNMNNMNNMNNMNNMNNMCTCTGGCCCTGGTGGTTGGT
lco9-dsSyn	ACCAACCACCAGGGCCAGAG
F-cap-recovery	CCCTGCAGACAATGCGAGAGAATGAATCAGAATTCAAATATCTGC
R-cap-recovery	ATGCATATGGAAACTAGATAAGAAAGAAATACG
External_Seq_F	TGTGGATTTGGATGACTGC
External_Seq_R	GACCAAAGTTCAACTGAAACG
internal_cap_Seq	GTTCTTGATGAGAATCTGTGGAGGAGG
EGFP-F	TCAAGATCCGCCACAACATC
EGFP-R	TTCTCGTTGGGGTCTTTGCT
WPRE_R	GGATTTATACAAGGAGGAGAAAATGAAAG
lco2/lco9-NGS_R	CGGTTTATTGATTAACAGGCAATTACAGATTACG
lco2_PepLib_F	RCCACTAACCCTGTGGCC
lco9_PepLib_F	AACGAAGAGGAAATTAAAACCACTAACC
PepLib-R	CGTCTCTGTCTTGCCACACC
BC_F01	GTTCAGCTGGAGTTCGTGACCGCCG
BC_F02	GTCATGCTGGAGTTCGTGACCGCCG
BC_F03	CTGTAGCTGGAGTTCGTGACCGCCG
BC_F04	GTATTGCTGGAGTTCGTGACCGCCG
BC_F05	CTAGTGCTGGAGTTCGTGACCGCCG
BC_R	CAACATAGTTAAGAATACCAGTCAATCTTTCACAAATTTTGTAATCCAGAGG
hALB_F	TGCTGTCATCTCTTGTGGGCTG
hALB_R	AACTCATGGGAGCTGCTGGTTC
Pep2hSC1-F	GATTCGTGGTCTGAGGCGGCCGCGACCGCA
Pep2hSC1-R	ACCCCGCTTCCTCTGGCCCTGGAGGTTGGT
Pep2hSC2-F	GTTGATTTTGATAAGGCGGCCGCGACCGCA
Pep2hSC2-R	ATCCTTCCTCCTCTGGCCCTGGAGGTTGGT
