# Supplementary material for: Novel AAV variants with improved tropism for human Schwann cells
Source: Mol Ther Methods Clin Dev. 2024 Mar 11;32(2):101234. doi: 10.1016/j.omtm.2024.101234 (PMC10978538; doi:10.1016/j.omtm.2024.101234)
Supplement: Document S2. Article plus supplemental information [file mmc2.pdf]

Molecular Therapy
Methods & Clinical Development
Original Article

Novel AAV variants with improved tropism for human Schwann cells

Matthieu Drouyer,[1] Tak-Ho Chu,[2] Elodie Labit,[3] Florencia Haase,[1] Renina Gale Navarro,[1] Deborah Nazareth,[1] Nicole Rosin,[3] Jessica Merjane,[1] Suzanne Scott,[1] Marti Cabanes-Creus,[1] Adrian Westhaus,[1] Erhua Zhu,[4] Rajiv Midha,[2] Ian E. Alexander,[4,5] Jeff Biernaskie,[2,3,6] Samantha L. Ginn,[4,9] and Leszek Lisowski[1,7,8,9]

[1]Translational Vectorology Research Unit, Children's Medical Research Institute, Faculty of Medicine and Health, The University of Sydney, Westmead, NSW, Australia; [2]Department of Clinical Neurosciences and Hotchkiss Brain Institute, Cumming School of Medicine, University of Calgary, Calgary, AB, Canada; [3]Faculty of Veterinary Medicine, University of Calgary, Calgary, AB, Canada; [4]Gene Therapy Research Unit, Children's Medical Research Institute and Sydney Children's Hospitals Network, Faculty of Medicine and Health, The University of Sydney, Westmead, NSW, Australia; [5]Discipline of Child and Adolescent Health, Faculty of Medicine and Health, The University of Sydney, Sydney, NSW, Australia; [6]Department of Surgery, Cumming School of Medicine, University of Calgary, Calgary, AB, Canada; [7]Australian Genome Therapeutics Centre, Children's Medical Research Institute and Sydney Children's Hospitals Network, Westmead, NSW, Australia; [8]Laboratory of Molecular Oncology and Innovative Therapies, Military Institute of Medicine - National Research Institute, Warsaw, Poland

Gene therapies and associated technologies are transforming biomedical research and enabling novel therapeutic options for patients living with debilitating and incurable genetic disorders. The vector system based on recombinant adeno-associated viral vectors (AAVs) has shown great promise in recent clinical trials for genetic diseases of multiple organs, such as the liver and the nervous system. Despite recent successes toward the development of novel bioengineered AAV variants for improved transduction of primary human tissues and cells, vectors that can efficiently transduce human Schwann cells (hSCs) have yet to be identified. Here, we report the application of the functional transduction-RNA selection method in primary hSCs for the development of AAV variants for specific and efficient transgene delivery to hSCs. The two identified capsid variants, Pep2hSC1 and Pep2hSC2, show conserved potency for delivery across various *in vitro*, *in vivo*, and *ex vivo* models of hSCs. These novel AAV capsids will serve as valuable research tools, forming the basis for therapeutic solutions for both SC-related disorders or peripheral nervous system injury.

INTRODUCTION

Gene therapy is poised to transform medicine as it carries the promise to treat, or even cure, millions of patients living with debilitating and currently incurable genetic disorders. As exemplified by the successful treatment of spinal muscular atrophy (SMA),[1] recombinant viral vectors based on adeno-associated viral vectors (AAVs) are showing immense therapeutic promise in targeting organs that necessitate direct *in vivo* gene delivery.[2] These include the liver and the nervous system, in which therapeutic efficacy has already been demonstrated. The current challenge in the field is to enhance vector-mediated gene delivery to target tissues of high therapeutic value that are more difficult-to-access, such as Schwann cells (SCs).[2,3] Importantly, these recent clinical successes hinge on continuing progress in the development of AAV-based gene delivery systems, driven primarily by advances in AAV capsid technology.[4]

SCs are the primary glial cell in the peripheral nervous system and play essential roles in ensuring physiological functions of peripheral nerves. They achieve this by providing support functions and secreting key signaling molecules critical for both axonal maintenance and repair.[5] Due to their ability to promote axonal regeneration and remyelination following nerve injury SCs are the primary clinical target in peripheral nerve-related neuropathies.[6–8] One such example is a group of inherited demyelinating neuropathies collectively known as Charcot-Marie-Tooth (CMT) disease, caused by mutations in genes such as *PMP22*, *GJB1*, or *SH3TC2*, whose proteins are expressed in myelinating SCs (nmSCs).[9] Another potential target is the tumor forming SCs that underlie neurofibromatosis 1 (NF1), NF2, and schwannomatosis.[10,11] Although the resulting tumors are typically benign, these debilitating diseases are often disfiguring, cause intractable pain and secondary neurological dysfunction, which significantly impair patient quality of life. Recent advances in gene therapy technologies have enabled the delivery of a functional copy of a disease-causing gene in gene replacement strategies, or, alternatively, correction, replacement, or silencing of disease-causing genes at the endogenous locus.[10–14] However, the success of all gene therapy strategies depends on the ability to deliver the therapeutic cargo

Received 11 July 2023; accepted 8 March 2024;
https://doi.org/10.1016/j.omtm.2024.101234.

[9]These authors contributed equally

Correspondence: Samantha L. Ginn, Gene Therapy Research Unit, Children's Medical Research Institute and Sydney Children's Hospitals Network, Faculty of Medicine and Health, The University of Sydney, Westmead, NSW, Australia.
E-mail: sginn@cmri.org.au
Correspondence: Leszek Lisowski, Translational Vectorology Research Unit, Children's Medical Research Institute, Faculty of Medicine and Health, The University of Sydney, Westmead, NSW, Australia.
E-mail: llisowski@cmri.org.au

© 2024 The Authors.
This is an open access article under the CC BY license (http://creativecommons.org/licenses/by/4.0/).

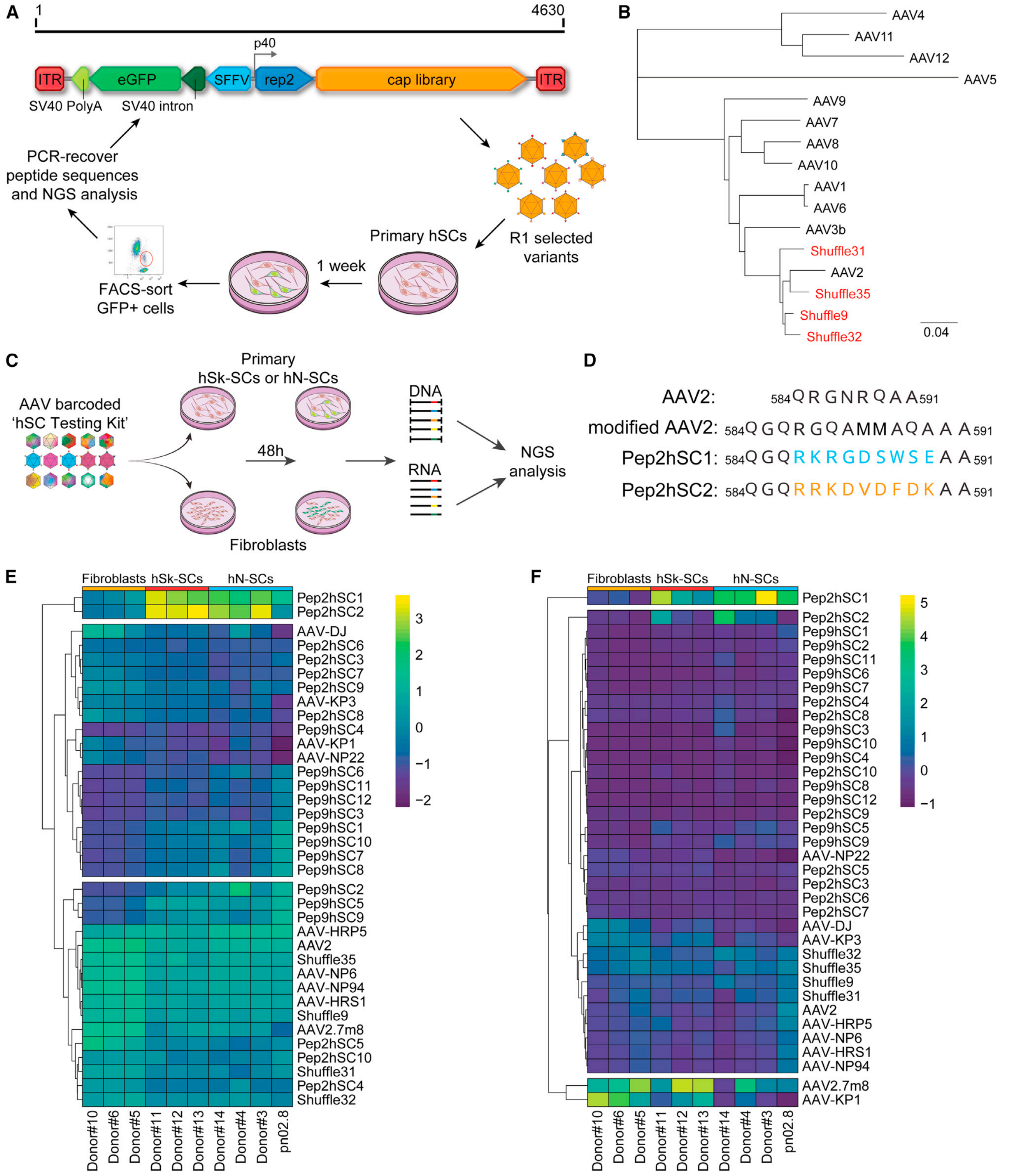

Figure 1. FT selection platform for AAV capsids targeting primary hSCs

(A) Overview of the capsid variant selection using the FT platform. (B) Phylogenic relation of the selected AAV shuffling capsid variants and the parental AAV serotypes used to construct the library. Scale: evolutionary distance of the number of substitutions per site. (C) Schematic representation of the barcoded-AAV hSC testing kit for NGS

(legend continued on next page)

specifically and efficiently to the relevant target cells. Thus, novel delivery vectors capable of efficiently targeting the SCs are needed to enable the translation of promising preclinical programs into clinically validated solutions for patients affected by genetic and possibly even acquired disorders affecting SCs.

A central feature of recombinant AAVs (rAAVs) is their capsid-driven tissue tropisms.[15] Several AAV vectors have demonstrated tropism for SCs with different degrees of success between variants. Notably, AAV1, AAV2, AAV6, and AAV-DJ have been shown to transduce primary human Schwann cells (hSCs), with AAV6 and AAV-DJ being the most efficient.[10] In rat nerve explants, AAV1, AAV5, AAV6, AAV7, AAV8, and AAV9 were the most efficient variants, whereas, in human nerve explants, AAV2 is shown to be the most effective.[16] AAV9, in combination with the *Mpz* promoter, has been used in mice to target SCs in X-linked CMT after a single lumbar intrathecal injection.[13] AAV9 and AAV-rh10 have been shown to transduce SCs in mice, rats, and non-human primates (NHPs) after intraneural injection.[14] In addition, a single injection of AAV9 in a rat model of CMT1A has been shown to prevent dysfunctions in CMT1A.[14] However, despite promising results in these preclinical models, the aforementioned AAV variants lack specificity and show broad off-target tropism, significantly decreasing their translational potential.[17]

Recent advances in AAV capsid bioengineering strategies, including rational design and directed evolution, have demonstrated the ability to select for novel clinically translatable properties of rAAV.[18] However, the success of such strategies depends on the interplay of three critical elements: the starting AAV library, the selection strategy or platform, and the preclinical model on which the selection and subsequent validation of AAV variants are performed.[19]

In this study, we aimed to develop novel AAV variants capable of targeting primary hSCs with high efficiency and specificity for translational applications. We utilized our recently developed proprietary functional transduction (FT)-RNA selection method[19] to select a highly variable AAV2-based peptide display library. Following two rounds of selection in purified primary hSC cultures, we identified two novel AAV2 capsid variants, named Pep2hSC1 and Pep2hSC2. Because hSC cultures cannot recapitulate the complexity of the whole tissue containing various cell types and barriers, we next characterized these novel AAVs across mouse peripheral nerve injury model and *ex vivo* human nerve segments, to clarify the SC subtypes tropism of the novel AAV capsids. These variants also demonstrated a decrease in targeting human fibroblasts and a lower entry in primary human hepatocyte cells. Based on their high efficiency and specificity, these two new variants hold great promise to enable the development of the first SC-specific gene therapies in human patients.

RESULTS

Identifying AAV capsids with improved efficacy on primary hSCs using the FT platform

To identify novel variants of AAV vectors for efficient transgene delivery to hSCs, we performed AAV-directed evolution selections on cultured primary hSCs. We generated three capsid libraries: AAV2 and AAV9 peptide display libraries, as well as a DNA-shuffled library based on the capsid genes from AAV serotypes 1–12. The three libraries were cloned into our proprietary FT library constructs, which allows for efficient variant selection based on transgene expression at the RNA level.[19] Following packaging, all three AAV vector libraries were used to transduce primary hSC cultures. To increase both the stringency and the chance of selecting the most functional variants, transduced cells underwent fluorescence-activated cell sorting (FACS) based on the expression of FT library-encoded EGFP reporter (Figure 1A). EGFP-positive cells were subsequently used for RNA extraction and PCR recovery of most functional variants from AAV-encoded RNA/cDNA.

After two rounds of selection, we selected the 10 top variants from the AAV2 peptide display library, namely, Pep2hSC1–Pep2hSC10. Interestingly, we were able to detect only a very small number of EGFP-positive hSCs for the other two libraries (AAV9 peptide display and the shuffled library, data not shown) and thus opted to process the entire hSC population for capsid recovery. After two rounds of selection of the AAV9 peptide display library we identified 12 highly enriched capsids (Pep9hSC1–Pep9hSC12) in the RNA/cDNA extracted from the whole SCs population. The DNA-shuffled library underwent four rounds of selection. After sequencing 15 random clones, we identified 4 capsid variants (Shuffle9, 31, 32, and 35) from a phylogenetically distinct subpopulation closely related to AAV2 (Figure 1B). The four shuffled capsids were included in subsequent functional evaluations.

We next performed a functional evaluation of the selected 26 novel variants on hSCs. As reference controls, we included 10 previously published natural and bioengineered AAV variants that were identified using our published ‘AAV Testing Kit’ approach[20] for their ability to functionally transduce primary hSCs in culture (Figure S1). Specifically, we packaged two single-barcoded AAV expression cassettes encoding EGFP fluorescent reporter under the control of the ubiquitous human cytomegalovirus (CMV) immediate-early enhancer and promoter[20] per novel variant and reference control. Vectors were individually titrated and pooled at an equimolar ratio. The barcoded hSC testing kit was tested on cultured primary hSCs isolated from the skin (hSk-SCs) or from the sciatic nerve (hN-SCs) from multiple donors (Table S1).[21] Human dermal fibroblasts were also transduced in parallel to confirm the specificity of the capsids for targeting only the hSCs. After 48 h, cells were harvested, and DNA and RNA

comparison in primary hSk-SCs or the hN-SCs and primary human fibroblasts from different donors. (D) Identity of the amino acid peptide sequences of Pep2hSC1 and Pep2hSC2 inserted in a modified AAV2 capsid. (E and F) Analysis of barcoded variants with capsid recovery achieved at the level of both (E) cell entry (DNA) and (F) transgene expression (mRNA). Heatmap and clustering analysis of capsid performance as a percentage of total NGS reads for each cell type. Values are normalized to a pre-mix pool and are the average of two barcodes.

extracted for subsequent NGS analysis of the barcode composition at the DNA (cell entry) and RNA/cDNA (transgene expression) levels, as per our established method (Figure 1C).[20]

For viral entry (DNA biodistribution) (Figure 1E), the analysis identified two variants, RKRGDSWSE and RRKDVDFDK, referred as Pep2hSC1 and Pep2hSC2, respectively (Figure 1D), with relatively high enrichment in both hSk-SCs and hN-SCs, and which were almost absent in fibroblasts. For transgene expression (RNA/cDNA) (Figure 1F), Pep2hSC1, and to a lesser extent Pep2hSC2, were the most enriched in both hSk-SCs and hN-SCs. Both variants also demonstrated high specificity for SCs, unlike AAV2.7m8, which performed very well regardless of the target cell type. Finally, AAV-DJ, which has been previously reported to be the most efficient at transducing hSCs,[10] was outperformed by AAV2.7m8 and our novel variants. Non-clustered heatmap visualizations of DNA and RNA/cDNA analysis for all cell lines are shown in Figure S2.

To evaluate whether the AAV variants selected in primary hSCs could also transduce primary cells from other species, we performed the same AAV barcoded hSC testing kit analysis on primary rat and mouse SCs (Figures S3 and S4, respectively). The bioengineered AAV-HRP5, a variant that we previously selected for superior homologous recombination in HuH-7 cells,[22] was the top performer both at cell entry (DNA) and transgene expression (RNA/cDNA) levels in rat cells while AAV-KP1[23] was the top variant in mouse SCs (Figure S4). Interestingly, Pep2hSC1 and Pep2hSC2, efficiently transduced primary rat SCs with 60% and 40% of EGFP-positive cells, respectively. AAV2.7m8 did not transduce rat SCs efficiently (17%) (Figure S3). Pep2hSC1 and Pep2hSC2 were able to transduce primary mouse SCs. However, EGFP expression was also observed in non-SCs cells (difference in morphology) following Pep2hSC1 transduction, while EGFP expression was strictly limited to SCs with Pep2hSC2 (Figure S4).

Characterization of Pep2hSC1 and Pep2hSC2

For further characterization, we decided to focus on two variants, Pep2hSC1 and Pep2hSC2. As manufacturability is one of the biggest bottlenecks impacting the clinical development of many novel bioengineered variants, we first evaluated the production yields of the variants using standard adherent HEK293T production protocols (Materials and methods). Our data showed that there was no significant difference in manufacturability between the novel variants and the parental AAV2 capsid (Figure S6A). As clinical grade AAV2 has been produced at scale to support clinical studies, the data indicate that the novel SC-tropic variants would be amenable to large scale production using standard AAV manufacturing protocols.

Based on the relative low performance of both Pep2hSC1 and Pep2hSC2 compared with AAV2 and other variants that harbors the heparin-binding motif of AAV2 (AAV-HRS1, AAV-HRP5, AAV-NP6, AAV-NP94, and the Shuffled variants) in immortalized hSCs (Figure S5), and the fact that peptide insertions at positions 587–588 of AAV2 capsid sequence reduces the heparan sulfate proteoglycan (HSPG)-binding phenotype,[24,25] we hypothesized that Pep2hSC1 and Pep2hSC2 were HSPG detargeted and their transduction was not dependent on heparan sulfate binding. To test this, we performed a heparin competition assay. As expected, the transduction efficiency of AAV2 was significantly decreased by soluble heparin. However, the performance of Pep2hSC1 and Pep2hSC2 was unaffected (Figure S6B), supporting our hypothesis regarding to the heparan sulfate binding of those new variants.

Because it is known that peptide insertions at this location not only alter vector tropism, but also impact the structure of the most protruding surface exposed loop, this modified structure had the potential to impact recognition by pre-existing neutralizing antibodies. We, therefore, performed a neutralization assay with serial dilutions of human intravenous immunoglobulin (IVIg). Both novel variants demonstrated significantly improved immune escape properties compared with the parental AAV2 capsid (Figure S6C).

Finally, we investigated the utility of Pep2hSC1 and Pep2hSC2 for systemic delivery by assessing the off-target transduction of those vectors *in vivo*. Specifically, because the liver is a natural target for most natural and bioengineered AAVs,[17] and thus presents a major safety concern, we wanted to evaluate our new variants for their ability to transduce primary human hepatocytes *in vivo* using the chimeric FRG model.[26]

Chimeric mice repopulated with primary human hepatocytes to a similar high repopulation index[27] were intravenously administered with a dose of 2×10^{11} vector genomes (vgs), representing approximately 1×10^{13} vg/kg, of Pep2hSC1, Pep2hSC2, or the parental AAV2, encoding CMV-EGFP-pA reporter construct (Figures S6D–S6F). The relative performance in human hepatocytes was assessed by immunofluorescence (Figures S6D and S6E) and vector entry into human hepatocytes at the DNA level (Figure S6F) 2 weeks after injection. While Pep2hSC1 and Pep2hSC2 exhibited a higher transgene expression in human hepatocytes when compared with parental AAV2 (Figure S6E), both variants showed a diminished entry into these cells, as evident by a respective 5-fold and 2.5-fold lower average vector copy number per diploid human genome (Figure S6F). However, the difference in cell entry was not statistically significant compared with AAV2.

Novel variants show a strong tropism for primary hSCs

We next wanted to perform a detailed functional evaluation of the two new variants on primary hSCs compared with control capsid AAV-DJ, which has been reported to efficiently transduce hSCs.[10] We also included AAV2.7m8 that, based on our studies (Figures 1E and 1F), was highly efficient at transgene delivery to hSCs. We transduced primary hSCs using a low dose of 1,000 vg/cell and harvested the cells three days later. Transduction efficiency was then determined by immunofluorescence (Figures 2A and 2C) and by flow cytometry (Figure S8A). Our data supported the next-generation sequencing (NGS) results (Figures 1E and 1F) and showed that the two novel AAV variants, Pep2hSC1 and Pep2hSC2, transduced

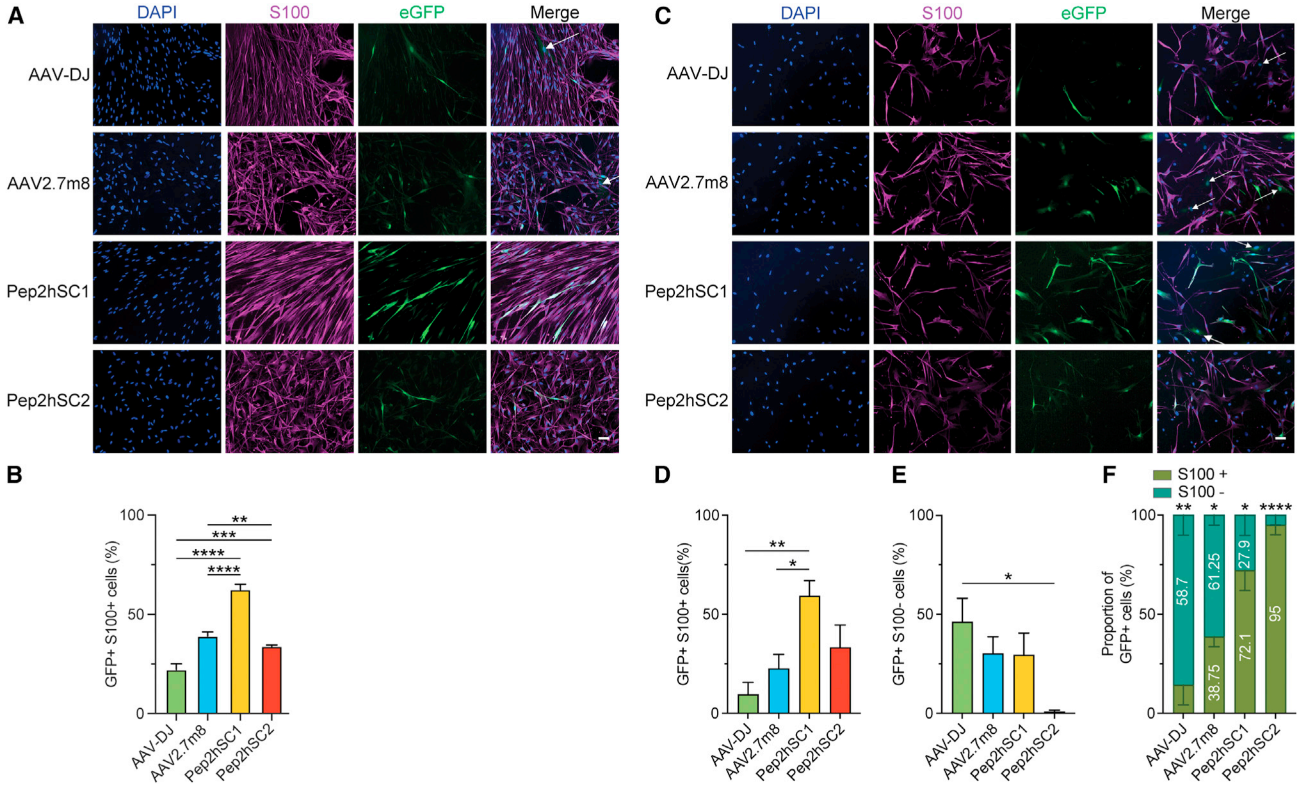

Figure 2. Novel AAV variants transduce hSCs with high efficiency and specificity

(A) Representative images of pure cultured hSCs transduced with EGFP reporter AAVs packaged using indicated capsids at 1,000 vg/cell. Blue, DAPI; purple, S100 (SCs marker); green, AAV-encoded EGFP. Arrows show EGFP$^+$/S100$^-$ cells. Scale bar, 50 μm. (B) Percentage of EGFP$^+$ SCs. Quantification was performed using $\geq$80 cells per image, and $\geq$3 images per variant. p values were determined by one-way ANOVA with Holm-Šidák's multiple comparison test (**$p \leq 0.01$; ***$p \leq 0.001$; ****$p \leq 0.0001$). Data are shown as mean ± SEM. (C) Representative immunofluorescence images of mixed cultured hSCs transduced with EGFP reporter AAVs packaged using indicated capsids at 1,000 vg/cell. For (A) and (C), arrows indicate EGFP$^+$/S100$^-$ cells. Scale bar, 50 μm. (D and E) Percentage of (D) EGFP$^+$/S100$^+$ cells and (E) EGFP$^+$/S100$^-$ cells. Quantification was performed using $\geq$30 cells per image, and $\geq$4 images per variant. p values were determined by one-way ANOVA with Holm-Šidák multiple comparison test (*$p \leq 0.05$; **$p \leq 0.01$). Data are shown as mean ± SEM. (F) Proportion of EGFP$^+$ cells in the mixed hSC culture. Percentages of S100$^+$ and S100$^-$ cells among total EGFP$^+$ cells were calculated. p values were determined by unpaired t-test (*$p \leq 0.05$; **$p \leq 0.01$; ****$p \leq 0.0001$).

hSCs with greater efficiency than both AAV-DJ and AAV2.7m8 (Figures 2A, 2B, and S8A).

Next, we determined the specificity of the novel variants at transducing primary hSCs. To do so, we used the pn09.2 culture, which contained both S100$^+$ (approximately 60%) and S100$^-$ cell populations (approximately 40%) as demonstrated by colocalization with the SCs marker S100β (Figure S7). Cells were transduced as outlined above (Figures 2A and 2B). Quantification of the EGFP signal in the S100$^+$ population 3 days after transduction showed that both Pep2hSC1 and Pep2hSC2 had greater efficiency at functionally transducing hSCs than AAV-DJ and AAV2.7m8 (Figure 2D). Furthermore, while Pep2hSC1 transduced the S100$^-$ population at similar efficiency to AAV2.7m8, Pep2hSC2 did not transduce S100$^-$ cells at a detectable level (Figures 2E and 2F). We repeated the study using a higher dose of 10,000 vg/cell, and we observed a similar trend, with Pep2hSC1 and Pep2hSC2 transducing more than 75% of hSCs (S100$^+$) and Pep2hSC2 not transducing S100$^-$ cells (Figures S9B and S9C). To gain additional insights into the function of Pep2hSC2, we transduced fibroblast cultures with a higher vector dose. Remarkably, Pep2hSC2 did not transduce fibroblasts, even when the highest dose was used (Figure S8C). Finally, by plotting the data as the percentage of the total transduced EGFP$^+$ cells, Pep2hSC1 and Pep2hSC2 clearly showed a preferential tropism to SCs (S100$^+$) over the S100$^-$ population at both doses tested (Figures 2F and S9D). Conversely, AAV-DJ and AAV2.7m8 displayed a stronger preference for transduction of fibroblasts (S100$^-$) over SCs (S100$^+$) (Figure 2F) at 1,000 vg/cell, and this preference toward fibroblasts was further enhanced at 10,000 vg/cell (Figure S9D).

Recognizing the crucial role of SCs in disorders like neurofibromatosis, particularly in the formation of plexiform neurofibromas,[28,30] we evaluated the performance of Pep2hSC1 and Pep2hSC2 in primary hSCs isolated from a plexiform neurofibroma, namely pNF01.3. This cell line harbors a mutation in exon 5 (c.565A>T, 787T) of the NF1 gene. The culture contained approximately 90%

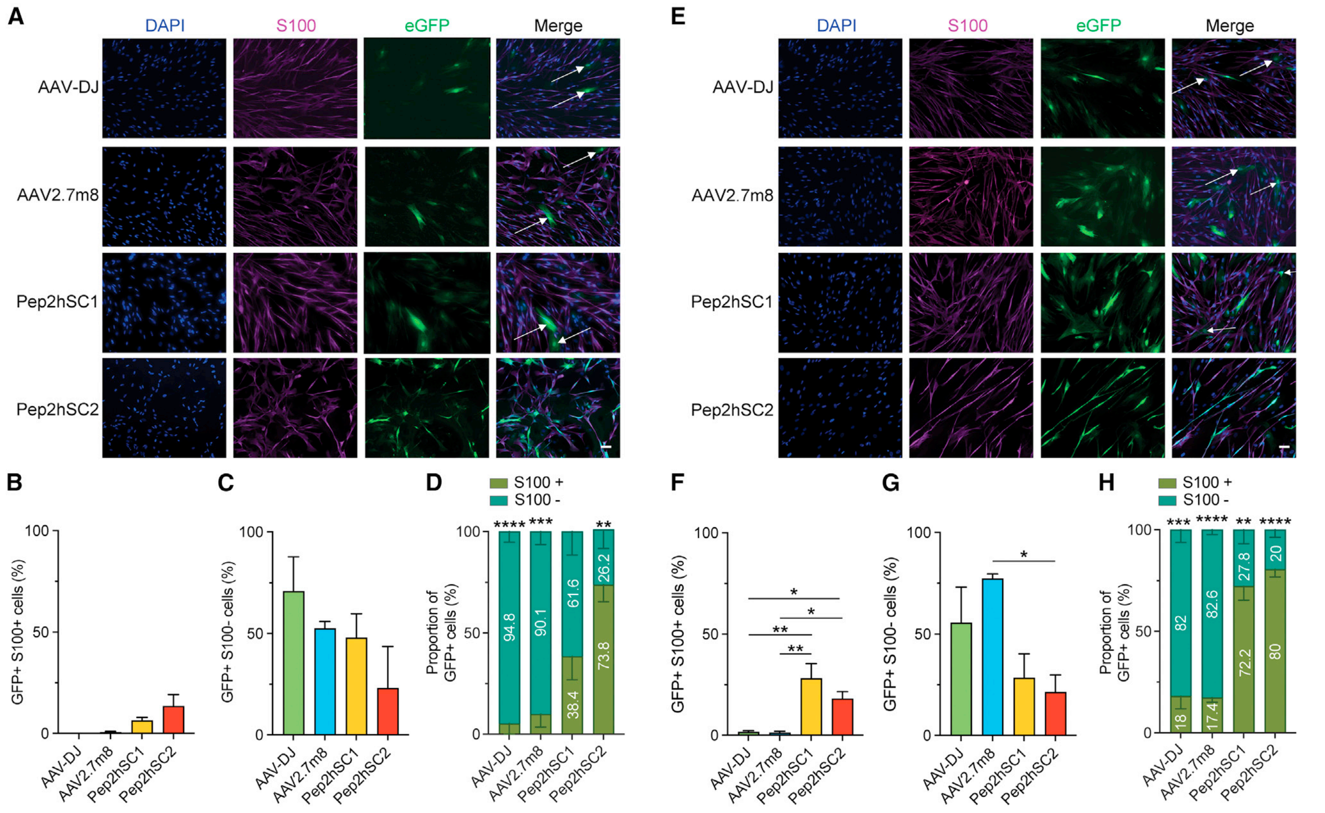

Figure 3. Novel AAV variants showed enhanced transduction in hSCs isolated from NF1 plexiform neurofibroma compared with AAV-DJ and AAV2.7m8
Functional analysis of indicated AAVs in hSC isolated from NF1 plexiform neurofibroma transduced at (A–D) 1,000 or (E–H) 10,000 vg/cell. Representative images of hSCs transduced with indicated AAVs encoding EGFP reporter at (A) 1,000 vg/cell or (E) 10,000 vg/cell. Blue, DAPI; purple, S100 (SCs marker); green, AAV-encoded EGFP. Arrows show $EGFP^+/S100^-$ cells. Scale bar, 50 μm. (B and C) Percentage of (B) $EGFP^+/S100^+$ cells and (C) $EGFP^+/S100^-$ cells. (D) Proportion of $EGFP^+$ cells in the mixed hSC culture. Percentages of $S100^+$ and $S100^-$ cells among total $EGFP^+$ cells were calculated. p values were determined by unpaired t-test (**$p \leq 0.01$; ***$p \leq 0.001$; ****$p \leq 0.0001$). (F and G) Percentage of (F) $EGFP^+/S100^+$ cells and (G) $EGFP^+/S100^-$ cells. (H) Proportion of $EGFP^+$ cells in the mixed hSC culture. Percentages of $S100^+$ and $S100^-$ cells among total $EGFP^+$ cells were calculated. p values were determined by unpaired t-test (**$p \leq 0.01$; ***$p \leq 0.001$; ****$p \leq 0.0001$). (B, C, F, and G) Quantification was performed using ≥30 cells per image, and ≥4 images per variant. p values were determined by one-way ANOVA with Holm-Śidák's's multiple comparison test (*$p \leq 0.05$; **$p \leq 0.01$). Data are shown as mean ± SEM.

SCs ($S100^+$), as shown in Figure S7. We transduced the pNF01.3 SC culture at 1,000 and 10,000 vg/cell and analyzed the EGFP signal by immunofluorescence 3 days after transduction.

The transduction levels of the four AAV variants in pNF01.3 culture were significantly lower than those observed in previous experiments involving healthy hSCs isolated from nerve tissue (Figure 2). At 1,000 vg/cell, Pep2hSC1 and Pep2hSC2 showed higher transduction in $S100^+$ cells, at 6% and 13.4%, respectively. In contrast, AAV-DJ and AAV2.7m8 exhibited minimal transduction with no detectable EGFP fluorescence in these cells (Figures 3A and 3B). These findings were a stark contrast to the results obtained from healthy hSCs (Figure 2).

Interestingly, in the $S100^-$ cell population, Pep2hSC1, AAV-DJ, and AAV2.7m8 achieved relatively high transduction efficiencies (70%, 52%, and 48%, respectively), while Pep2hSC2 transduced less than 25% of the cells (Figure 3C). When the dose was increased to 10,000 vg/cell, the pattern remained consistent, with negligible transduction for AAV-DJ and AAV2.7m8. However, Pep2hSC1 and Pep2hSC2 showed improved transduction in $S100^+$ cells by 4.6-fold and 1.4-fold, respectively (Figure 3F). Notably, the enhanced transduction efficiency of $S100^+$ cells with Pep2hSC1 was accompanied by an increased transduction rate in $S100^-$ cells (77%), while Pep2hSC2 showed no significant difference in this group (<25%) (Figure 3G). Finally, both Pep2hSC1 and Pep2hSC2 display a preferential tropism for SCs ($S100^+$) compared with the $S100^-$ population, even at the higher dose tested (Figures 3D and 3H).

Together these results demonstrate that Pep2hSC1 and Pep2hSC2 display superior performance over AAV-DJ and AAV2.7m8 in primary hSCs derived from healthy donors and NF1 patients. Furthermore, Pep2hSC2 exhibited an improved specificity for the SCs, with almost no transduction of fibroblast.

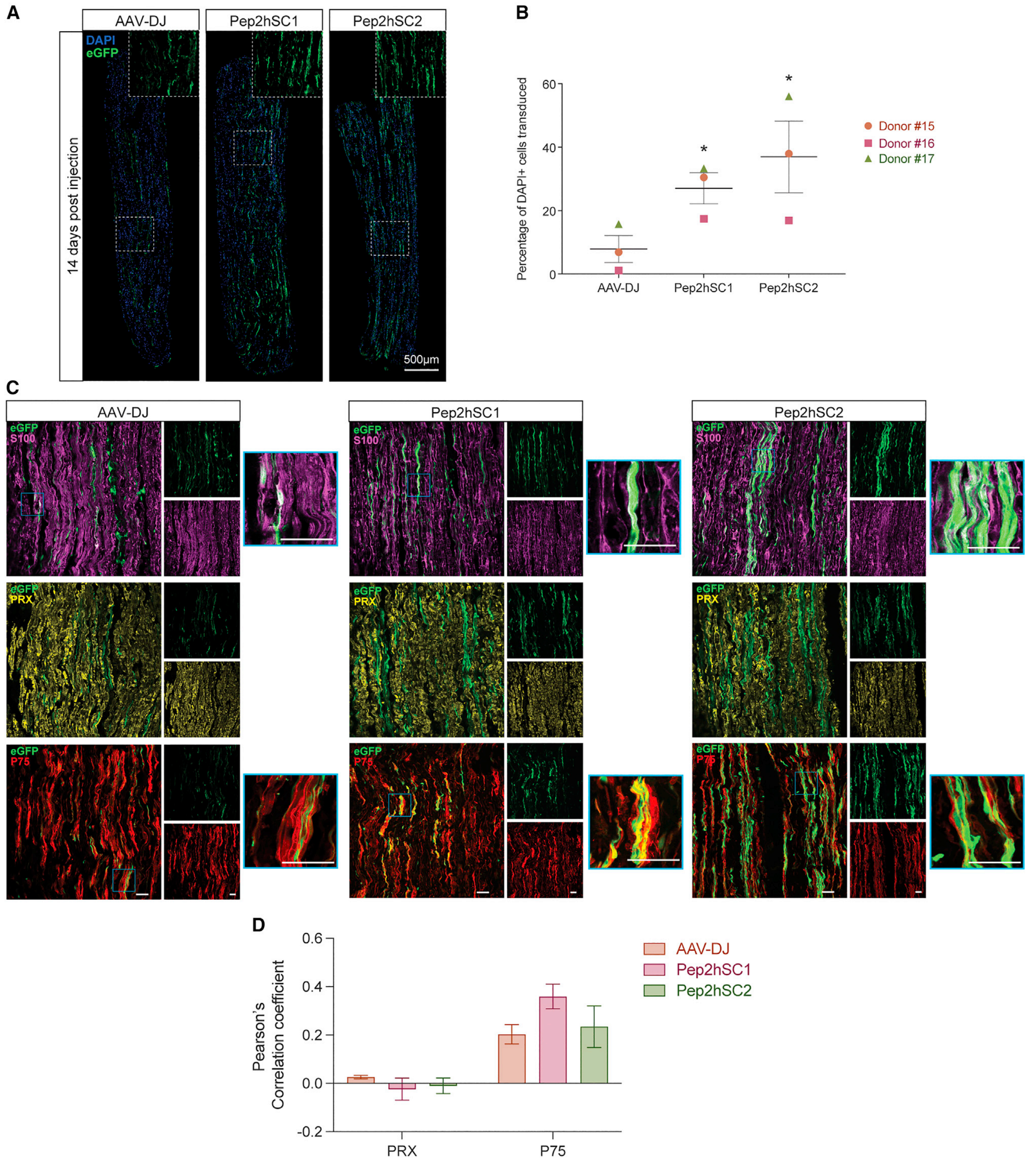

Figure 4. Novel AAV variants transduce SCs in human nerve segments

(A) Immunofluorescence of longitudinal sections of human sural nerve segments at 14 days after injection. Nerve segments (0.5cm) were injected with AAV-DJ, Pep2hSC1, or Pep2hSC2 vectors encoding a CMV-EGFP transgene (1×10^{10} vg dose per segment). Insets show magnified area of transduced cells with elongated morphology characteristic of SCs. DAPI (blue), AAV-encoded EGFP (green). Scale bar, 500 μm. (B) Number of EGFP^{+} cells overlapping with DAPI marker in nerve segment 14 days after injection from three donors (mean ± SEM; n = 3). p values were determined by unpaired t-test (*$p \leq 0.05$). (C) Confocal microscopy images of longitudinal sections

(legend continued on next page)

Evaluation of novel capsids for transduction efficiency of human nerve segments

Assessment of AAV tropism in cell cultures, while highly informative, has its limitations; the SCs in culture do not exhibit the same structures and differentiation as SCs surrounding neuronal axons. Indeed, it has been shown previously that the transduction of monolayers of cultured SCs does not predict the transduction efficiency in nerve segments.[16]

Therefore, we subsequently examined the capacity of the novel variants to transduce SCs in the context of human nerve segments. Sural nerve fascicles (approximately 0.5 cm long) from three individual donors (n = 1 segment per serotype and per donor) were injected with 1×10^{10} vg of either Pep2hSC1, Pep2hSC2, or AAV-DJ and assessed for expression of EGFP protein 7 and 14 days after AAV administration (Figures 4 and S10). Scanned sections showed a higher number of $EGFP^{+}$ cells and a stronger EGFP intensity for Pep2hSC1 and Pep2hSC2 after 7 days compared with AAV-DJ (Figure S10). On day 14, immunofluorescent analysis showed that transduction with AAV-DJ results in a significantly lower percentage of $EGFP^{+}$ cells than the novel AAV variants Pep2hSC1 and Pep2hSC2 (Figures 4B and S10). Of note, for donor 16, the EGFP signal was localized near the injection site, while the EGFP signal was scattered throughout the nerve segments for donors 15 and 17. Additionally, donor 17 exhibited an overall lower number of $EGFP^{+}$ cells than the other two donors.

Confocal imaging of the nerve segment revealed that the transduced cells had an elongated morphology with oval-shaped nuclei characteristic of hSCs. This was also confirmed with the co-labelling of EGFP and S100β (Figures 4C and S11). To further explore the tropism of Pep2hSC1 and Pep2hSC2, we performed a series of double-labelling immunohistochemistry experiments and Pearson' correlation coefficient analysis to determine the type of hSCs that were transduced. Interestingly, EGFP was rarely expressed by PRX-labelled mSCs. However, the transduced SCs from donors 15 and 16 were positive for p75 neurotrophin receptor (P75NTR) (Figures 4C, 4D, and S11), which is a marker specific for non-nmSCs or de-differentiated SCs.[29] Interestingly, the transduction of donor 17's cells showed co-expression of EGFP and PRX, suggesting that the novel variants can potentially transduce mSCs (Figure S11).

In summary, these results demonstrate that Pep2hSC1 and Pep2hSC2 capsids can transduce nmSCs and, to a lower extent, mSCs in human nerve tissue after direct injection into nerve fascicles *ex vivo*.

Transduction patterns differ between Pep2hSC1 and Pep2hSC2 in injured mouse sciatic nerve

Since the novel capsid variants also showed an improved transduction profile of rat and mouse SCs *in vitro*, we next used a mouse peripheral nerve injury model as a surrogate to gauge the transduction efficiency in human nerves.

Equal amounts of Pep2hSC1, Pep2hSC2, and AAV-DJ vectors encoding single-stranded AAV CMV-EGFP-pA cassettes were injected into the sciatic nerves of adult mice at 2×10^{10} vg per animal following sciatic nerve crush (Figure 5A). Four weeks later, longitudinal sections of the sciatic nerve were examined for EGFP expression (Figures 5B and 5C) and were co-stained for nmSC marker, PRX, and neurofilament (NF) axonal marker (Figures 5D and 5E).

Following sciatic nerve crush, we found that EGFP expression was more widespread after AAV-DJ injection compared to Pep2hSC1 and Pep2hSC2 with the biggest difference observed in the distal region (Figure 5B). However, similar levels of EGFP fluorescence were observed for all three vectors (Figure 5C). For all the vectors tested, many EGFP cells within the area proximal to the crushed site (uninjured segment) displayed a morphology resembling mSCs with distinct appearance of cytoplasmic bands (Figure 5D). Double labeling with the mSC marker PRX confirmed a close colocalization with EGFP (Figure 5D). Furthermore, staining with an NF antibody confirmed EGFP-ensheathed axons in a manner suggestive of myelin sheath formation (Figure 5D). Notably, this cell type bias contrasts with what we observed in the human nerve experiment, where the vectors showed a preference toward nmSCs (Figure 4D).

Interestingly, within the lesioned area in the distal nerve, we found that Pep2hSC1, Pep2hSC2, and AAV-DJ transduce distinct cell types (Figure 5E). Pep2hSC1-driven transgene expression was detected exclusively in axons, positive for NF. Conversely, for Pep2hSC2, EGFP expression was observed in mSCs, while AAV-DJ targeted mostly nmSCs. EGFP was assessed in the spinal cord and both AAV-DJ and Pep2hSC1 transduced motoneurons, with Pep2hSC1 demonstrating superior performance, while Pep2hSC2 demonstrated only limited transduction efficiency, with only one cell detected in the entire spinal cord (Figure S12).

Thus, these experiments indicate that Pep2hSC1 and Pep2hSC2 can transduce naive and injured murine SCs but exhibit unique tropism toward specific subtypes of SCs, particularly in the context of nerve injury.

DISCUSSION

Recent advances in bioengineering strategies, including rational design and directed evolution, have demonstrated the ability to design and select for novel, clinically meaningful properties of rAAV.[18,31] As a result, a significant effort has been committed to improving AAV vectors efficiency for clinically important tissue targets, notably the central nervous system,[32–34] the liver,[35,36] and muscle.[37]

immunostained for EGFP (green) with either S100 (purple) for SCs, PRX (yellow) for nmSCs, or P75 (red) for non-nmSCs. Blue outlined insets show magnified area of colocalization. Scale bar, 50 μm. (D) Pearson's correlation coefficient for colocalization of EGFP and PRX or P75 (mean ± SEM; n = 3). Donor 15 is a 42-year-old male, donor 16 is a 44-year-old male, Caucasian, and donor 17 is a 73-year-old male, Asian.

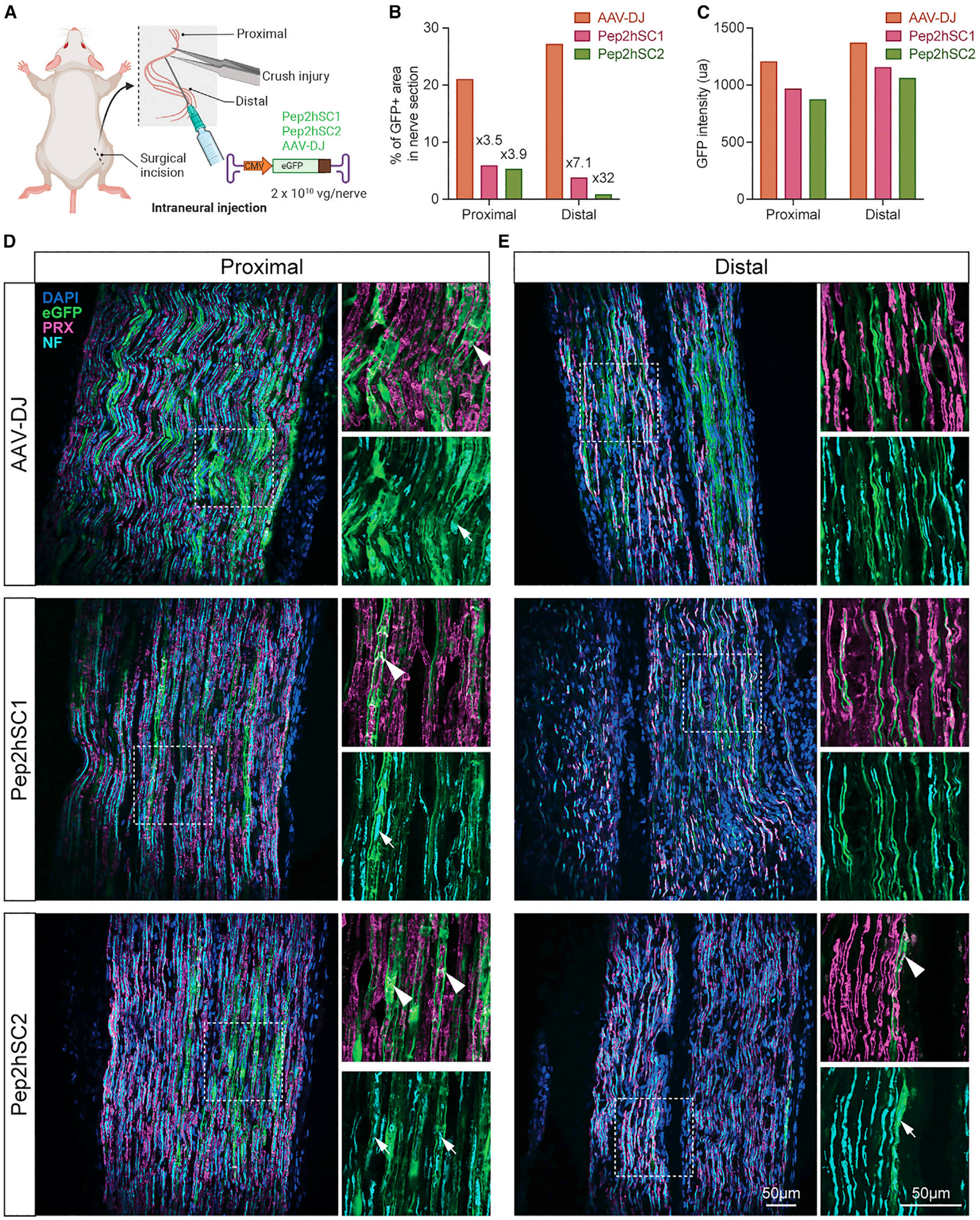

(legend on next page)

In this work, we report for the first time the development of AAV capsids bioengineered specifically to support efficient funtional transduction of primary hSCs. To improve the selection process, we used the powerful FT selection platform, which allows for robust and stringent selection of capsid candidates based on their ability to drive efficient transgene expression in the target cells, increasing the chance of selecting for highly functional variants from vector-encoded *cap* gene mRNA.[19] We performed an *in vitro* selection on primary hSCs and have identified two unique and promising variants, named Pep2hSC1 and Pep2hSC2. Both variants exhibited improved SCs transduction with greater specificity compared with AAV-DJ, the most functional variant for SC targeting reported to date.[10] We confirmed the hSCs transduction efficiency of Pep2hSC1 and Pep2hSC2 in a direct comparison using hSCs derived from primary nerve and skin from seven human donors of different ages and sexes. Interestingly, hSCs isolated from a plexiform neurofibroma were more resistant to AAV transduction than those isolated from nerve of healthy donors. This is a critical finding as it directly shows that data obtained from preclinical models based on healthy cells may not be the best predictor of vector function in the clinical setting where diseased cells will be targeted. It was highly encouraging that our novel AAV variants, Pep2hSC1 and Pep2hSC2, demonstrated the strongest performance in hSCs isolated from a plexiform neurofibroma among the AAVs tested, despite the fact the percentage of transduced S100$^+$ cells was lower than what was observed at the same dose in hSC isolated from healthy donors (Figures 2 and 3). Importantly from the perspective of *in vivo* therapeutic application, our variants showed higher specificity for hSCs in mixed SCs and fibroblast cultures. Specifically, Pep2hSC1 transduced fibroblasts at a level similar to control AAV2.7m8 and significantly less efficiently than AAV-DJ. In comparison, Pep2hSC2 did not transduce human fibroblasts, positioning this latter variant as a strong candidate for *in vivo* gene therapy applications. Of note, the specificity of Pep2hSC1 can be further improved with the use of the SC-specific promoters, such as the myelin-specific *Mpz* promoter,[38] which has been shown to drive a high level of expression in SCs.[39,40]

As exemplified by the successful treatment of patients with SMA using AAV9, which can cross the blood-brain barrier and transduce primary human neurons with high efficiency,[1] the clinical success of gene therapies depends on the ability to select a highly efficient vector at delivering the therapeutic cargo to the intended human tissue target. Given that an AAV vector for efficient and specific delivery to primary hSCs has not been previously reported, we have performed an AAV screen of highly variable libraries and subsequent validation of selected variants on primary hSCs. However, it is critical to consider that such a model cannot recapitulate the complexity of the whole tissue containing various cell types and barriers. Moreover, a study by Hoyng and colleagues[16] reported substantial AAV transduction differences between hSCs cultured as monolayers and SCs in nerve segments. Thus, we also included studies using human nerve segments. Those studies (Figure 4) also confirmed enhanced tropism of our variants toward SCs. A more detailed analysis confirmed that our novel variants preferentially transduced cells resembling nmSCs. This tropism toward nmSCs could be explained by the fact that we performed the selection process using primary hSCs in culture, which have been shown by Stratton and colleagues[41] to contain large proportions of immature SCs. Although we cannot exclude the possibility that these variants transduce dedifferentiated SCs or repair SCs, which are possibly present in the excised human nerve segment and share a similar appearance to nmSCs. However, the presence of numerous PRX-expressing mSCs even in 14-day explant cultures suggested that the degeneration process is extremely slow. Electron microscopy analysis of 14-day nerve explant cultures also demonstrated incomplete degradation of mSCs (data not shown), indicating that the phenotypes of SCs are likely preserved in nerve explant, reflecting a natural tropism toward nmSC rather than mSCs in human nerves.

It is essential to acknowledge the impact of nerve lesions on the observed tropism of Pep2hSC1 and Pep2hSC2. Nerve injuries, such as in our crush injury model, lead to significant alterations in the nerve, including reprogramming of SCs and changes in axonal properties. These modifications, occurring both immediately and during regeneration, can influence the transduction patterns of our AAV variants. Therefore, while our data indicate a preference for nmSCs and mSCs transduction, these findings might be influenced by the lesion-induced cellular environment. This highlights the need for further research to fully understand the behavior of these AAV variants in varying nerve conditions.

Most natural AAV isolates, and bioengineered variants target the liver with high efficiency. Liver off-target is a significant obstacle to the clinical development of therapies targeting organs other than the liver due to safety and cost implications. Indeed, liver toxicity has been recently reported in SMA patients treated with Zolgensma (AAV9-SMN)[42] and three fatalities related to liver toxicity have been reported to date following single intravenous administration of a high dose of AAV8 in a clinical trial targeting X-linked myotubular myopathy.[43,44] These alarming reports prompted us to evaluate our novel variants for their tropism toward human hepatocytes in a xenograft mouse model of the human liver. While Pep2hSC1 and Pep2hSC2 demonstrated higher transgene expression in human hepatocytes, both variants

Figure 5. Evaluation of novel AAV variants in sciatic nerve crush model

(A) Graphical illustration of AAV delivery following nerve crush injury. Following forceps-induced nerve injury, AAV-DJ, Pep2hSC1, or Pep2hSC2 were administered by intraneural injection (2×10^{10} vg dose per mouse) and sciatic nerves were harvested 4 weeks post-injection. (B) Quantification of the percentage of transduced area in proximal and distal region from the crushed site (transduction area has been reported as the percentage of EGFP stained area versus total nerve area). (C) Quantification of the mean EGFP intensity per transduced cell. (D and E) Longitudinal sections with DAPI (blue), EGFP (green), PRX (magenta) and neurofilament (NF) staining (light blue). (D and E) Representative images of (D) proximal region and (E) distal region from crushed site. Insets show a magnified views of selected areas highlighted by white dotted outline. Arrowheads indicate colocalization between EGFP and PRX (nmSC marker). Arrows show EGFP-labeled cells enclose the axons marked by NF staining. Scale bar, 50 μm.

showed reduced entry into those cells compared with parental AAV2, which could suggest that those variants have a lower risk of causing acute liver toxicity arising from viral load. It is also important to note that the use of cell/tissue-restrictive promoters and/or incorporation of microRNA target sequences (miR-122 specifically) into the 3′ UTR of the therapeutic transgenes would be used to suppress the off-target expression in the liver, further increasing the safety profile of gene therapies developed using these new AAV variants.[45–47]

The weak off-target effects of both variants in the human liver coupled with their ability to transduce hSCs with high efficiency and specificity (Figures 2, 3, and 4), could facilitate lower therapeutic vector doses. Importantly, lower doses not only lower the cost of clinical implementation, but also increases the overall safety profile of the vectors.

Ensuring translatability and clinical impact of newly generated vectors is an important consideration to address. To this end, cross-species tropism is an essential and highly desirable characteristic as it enables the preclinical studies to be performed in relevant *in vivo* preclinical models, such as mice or NHPs, without the need to utilize surrogate vector variants. Our data show that both novel variants reported here show good translatability between mice after intraneural injection and primary hSC cultures. However, the data also highlighted some inter-species differences regarding the specific types of SCs targeted by the vectors. Specifically, cell type characterization in murine *in vivo* studies showed that the novel variants had an improved tropism toward mSCs, in contrast with the tropism toward nmSCs observed in human nerve explant (Figure 4). This is not a surprise; it was previously shown that AAV tropism could differ in each animal model, as well as across species, due to cell surface receptor differences on the target cells.[48] A detailed examination of double-immunolabeled samples revealed that Pep2hSC1 and Pep2hSC2 transduced a minority of mSCs in human nerve explant from at least one human donor (Figure S11). To fully appreciate the clinical applicability of the new variants, further evaluations in human nerve explant from multiple donors will need to be performed. Moreover, in a crush injury model, the tropism was further biased toward axons and mSCs for Pep2hSC1 and Pep2hSC2, respectively. These observations are important considerations when planning applications of those new variants in hSC-related disorders. In addition, Pep2hSC1 and Pep2hSC2 have not yet been tested in the NHP model, and thus further studies are required to determine translatability in primates. Finally, alternate AAV delivery methods, such as intrathecal administration,[13,39,49] which has the advantage to be less affected by pre-existing anti-AAV neutralizing antibodies,[50] should also be evaluated in future studies.

In conclusion, we present, for the first time, novel AAV capsid variants, Pep2hSC1 and Pep2hSC2, bioengineered specifically for improved transduction efficiency and specificity of primary hSCs. Importantly, Pep2hSC1 and Pep2hSC2, demonstrate reduced vector entry into human primary hepatocytes, and stronger resistance to neutralization by IVIg than AAV2. Based on our data, these novel vectors may have the potential to address current limitations of AAV-based therapies targeting SCs.

MATERIALS AND METHODS

Cell culture conditions and cell origins

AAV production was performed using the HEK293T cell line (ATCC, Cat#CRL-3216) grown in DMEM (Gibco, Cat#11965) supplemented with 10% fetal bovine serum (FBS) (Sigma-Aldrich, Cat#F9423), 1× penicillin-streptomycin (PS) (Gibco, Cat#15070), and 25 mM HEPES (Gibco, Cat#15630).

Normal human neonatal dermal fibroblasts used for flow cytometry experiments were kindly provided by Associate Professor Anai Gonzalez-Cordero (CMRI). Cells were cultured and maintained and were grown in growth medium containing DMEM supplemented with 10% FBS, 1 × PS, 1 × L-glutamine (Gibco, Cat#25030-081) and 1× non-essential amino acids (Gibco, Cat#11140) in a humid 5% CO_2 incubator at 37°C.

Primary human SCs and fibroblasts

Human tissues were procured, with approval from the local institutional review board, via the Southern Alberta Donation Program and Human Organ Procurement and Exchange Program (Calgary, Canada) with donor or family consent. All samples were non-identifiable to the researchers in the study.

Human skin-derived SCs, dermal fibroblasts and nerve-derived SCs were isolated and purified according to protocol previously published.[21] Briefly, for skin-derived SCs, approximately 1cm^2 full thickness neck skin was removed from autopsy samples. The skin was washed three times in cold Hank's balanced salt solution (HBSS, Gibco) and then cut into 2- to 3-mm thin stripes and digested in 5 U dispase (Stemcell Tech) overnight at 4°C or 2–3 h at 37°C. The next day, the epidermis was removed and discarded and the dermis was cut into 1-mm pieces, and subsequently placed in 35-mm culture dishes and covered with a minimum volume of DMEM supplemented with 10% FBS and 1× PS, 50 ng/mL human recombinant neuregulin-1 (NRG1, Cat#100-03 Peprotech), 5 μM forskolin (Fsk) (Sigma), and 12.5 μg/mL Plasmocin (Invivogen) for 2 weeks as an explant culture. Media were replaced twice weekly. After 2 weeks, the skin explants were dissociated overnight (37°C and 5% CO_2) in DMEM with 10% FBS, 1.25 U dispase (StemCell, Cat#07913), and 1.25 mg/mL collagenase IV (Worthington, Cat#LS004288). The next day, the mixture was gently triturated with a 1-mL pipette, the dissociated cells were passed through a 40-μm cell strainer, and centrifuged at 300×g for 6 min at room temperature. After resuspension of the pellet in SC complete medium containing DMEM supplemented with 10% FBS and 1× PS, 50 ng/mL NRG1, and 5 μM Fsk, the cells were plated onto poly-D-lysine and laminin-coated culture dishes for initial expansion.

For nerve-derived SCs, human sciatic nerve samples were obtained from autopsy donors (Table S1). Individual nerve fascicles were pulled from 1 cm-long sciatic nerve, cut into 3-mm short segments, and subsequently placed in a 35-mm culture dishes as explant culture as described. Cell dissociation and initial expansion were identical to

skin-derived SCs. Samples were then dissociated and cultured as described.

After 1 week of expansion, mixed cell populations from both tissue origins were selected against p75 and Thy1 to isolate SCs and dermal fibroblasts, respectively. Hybridoma supernatants from 200-3-G6-4 (ATCC, Cat#HB-8373) and K117 (ATCC, Cat#HB-8553) were used. Purified cells were further expanded in SC complete medium before experimentation.

Primary human SCs (hSCs) pn02.8 and pn09.2 were kindly provided by Prof Margaret R Wallace. Media was changed every fourth day for both skin and nerve cells.

For the rat SC cultures, sciatic nerves were harvested from 6- to 8-week-old Wistar rats. All animal care and experimental procedures were approved by the joint CMRI and The Children's Hospital at Westmead Animal Care and Ethics Committee. Nerves were washed three times with ice-cold HBSS (Sigma, Cat#H9394). The epineurium was stripped off under a stereomicroscope, and the nerves were washed three times with ice-cold HBSS and were then transferred into a cell culture plate containing, serum-free DMEM. Short segments of 3–5 mm were cut and placed in a 35-mm Petri dish and covered with a minimum volume of DMEM supplemented with 10% FBS and 1× PS, 50 ng/mL NRG1 (Peprotech, Cat#100-03-100), and 5 μM Fsk (Sigma Aldrich, Cat#F6886) to keep them attached to the bottom of the dish. For 2 weeks, the medium was replaced twice a week. During this time, the majority of the endoneurial fibroblasts migrate out of the nerve segments onto the culture dish surface, whereas most of the SCs stayed in the nerve segments. After 2 weeks, the nerve segments were dissociated overnight (37°C and 5% CO_2) in DMEM supplemented with 25% dispase (StemCell, Cat#07913) and 0.125% collagenase IV (Worthington, Cat#LS004288). The next day, the mixture was gently triturated with a 1-mL pipette, the dissociated cells were passed through a 40-μm cell strainer and centrifuged at 300×g for 6 min at room temperature. After resuspension of the pellet in DMEM supplemented with 10% FCS and 1× PS, 50 ng/mL NRG 1, 5 μM Fsk, the cells were plated onto poly-D-lysine and laminin-coated Petri dishes.

SCs purity was expressed as the percentage of cells positive for S100β, and the total cell number was determined by DAPI staining.

AAV library preparation

The AAV2 peptide display library was generated as previously described.[19] For the AAV9 peptide display library, random heptamers were inserted between amino acids 588 and 589 of the AAV9 VP1 protein. In brief, double SfiI restriction sites were inserted into the local codon-optimized version of the AAV9 cap gene (Caplco9) at the Q588 insertion site. The resulting plasmid pRep2Caplco9_SfiI was then digested with SwaI and NsiI, and the capsid-containing fragment was ligated into the equally SwaI/NsiI digested FT-spleen focus-forming virus (SFFV) selection platform. This FT-SFFV-lco9_SfiI construct was subsequently digested twice with SfiI and was dephosphorylated using calf intestine alkaline phosphatase (NEB, Cat#M0290), using the manufacturer's protocol. The 7-mer random inserts were made double-stranded using a short primer binding on the homology arm upstream of the peptide (dslco9-library). The final library was generated by mixing 225 fmol of the digested FT-SFFV-lco9_SfiI backbone with 2,250 fmol of the dslco9-library insert into individual NEBuilder (NEB, Cat#E2621) reactions. The reactions were combined after assembly and purified using ethanol precipitation. The resulting pellet (1 μg of DNA) was used for electroporation into SS320 competent cells (Lucigen, Cat#60512). The recovered transformants were used to inoculate 250 mL of lysogeny broth containing 10 μg/mL trimethoprim. Only 10 μL of recovered transformants were used to plate a 5- to 10-fold dilution series of the electroporated bacteria to determine transformation efficiency. Total FT-SFFV-lco9_7mer library plasmid was purified with an EndoFree Maxiprep Kit (Invitrogen, Cat#A31217).

For the Shuffled AAVLib_1–12 capsid plasmid library, the AAV library was generated as previously described.[51] AAV variants 1–12 were included in the parental mix. To move the same library into the FT platform, the pRC-AAVLib_1–12 was digested overnight alongside the pFT-SFFV platform with SwaI and NsiI. We ligated 1.4 μg of the insert at 16°C with T4 DNA ligase (NEB, Cat#M0202) for 16 h into 1 μg of the pFT-SFFV platform. Ligation reactions were concentrated by using ethanol precipitation, electroporated into SS320 electro-competent bacteria, and grown as described above.

AAV library selection

The screening of AAV variants in cultured primary human SCs was based on the FT-based method.[19] The Lco2 capsid variants were selected by screening a lco2 peptide display library on hSCs *in vitro*. Approximately 2×10^{10} vgs of library were used to transduce the cultured primary hSCs for 24 h. At 7 days after transduction, DNA and RNA were isolated from cell pellets. Capsid variants DNA and expressed mRNA were amplified from the DNA and cDNA samples, respectively, with primers lco2_PepLib_F/PepLib-R surrounding the 7-mer insert. The amplicon was used for Gibson assembly into the twice SfiI-digested FT-SFFV-lco2 library recipient plasmid and electroporated into bacteria, as described in the AAV library preparation. The peptides recovered from the first round of screening were packaged and screened again in the cultured primary hSCs. Based on the NGS results, we ranked the recovered variants, and the top candidates were picked as described in the results and respective nucleotide sequences encoding these peptides were cloned into the cap gene of lco2 to obtain helper plasmids for producing capsid variants as vectors. Similar screening methods were performed with the lco9-based peptide display library. Briefly, the lco9-based peptide display was analyzed using NGS at every step of selection, including before selection (packaged library), after round 1 (FT-RNA), and after round 2 (FT-RNA), using primers lco9_PepLib_F/PepLib-R. We ranked the recovered variants and chose the top candidates for further tests as described below.

For the shuffled AAVLib_1–12 capsid library, selected AAV cap genes were recovered after four rounds of iterative passage in SCs

with flanking primers (F/R-cap-recovery). Briefly, genomic DNA was extracted, and the cap sequences were amplified by PCR using the F/R-cap-recovery primers (Table S3) and cloned directly with Gibson assembly into recipient plasmid. The reaction was used for electroporation and grown as above. The full cap was then excised using SwaI and NsiI and cloned into the FT-SFFV platform with 20 individual ligations at 16°C with T4 DNA ligase overnight. The ligations were combined and purified using ethanol precipitation. The resulting pellet (1 μg DNA) was used for electroporation into competent cells. AAV capsid ORFs from round 4 were cloned into standard packaging plasmid harboring *rep2* with Gibson assembly and 50 randomly chosen clones were sent for full Sanger sequencing with primers External_Seq_F/R and internal_cap_Seq (Table S3).

AAV packaging

All AAV vectors were produced in HEK293T cells by triple-transfection using PEI MAX (Polysciences, Cat#24765-1) to package two unique single-stranded *CMV*-EGFP-N_6*Barcode*(*BC*)-*WPRE* transgenes each.[20] The FT-lco2_7mer, FT-SFFV-lco9_7mer and FT-SFFV-AAVLib_1–12 libraries (alongside pRep2 helper plasmids) were produced in ten 15-cm dishes of HEK293T cells. For the CMV-GFP-N_6BC-WPRE construct, 5 μg transgene plasmid was transfected per 15-cm dish, while to reduce cross-packaging, 400 ng transgene plasmid was transfected per 15-cm dish for library production. Three days after transfection, recombinant virus was harvested from the cells and media and purified by ultracentrifuge using iodixanol gradient as previously described.[52] AAV titers were quantified by droplet digital PCR (ddPCR) (Bio-Rad) using EvaGreen supermix (Bio-Rad, Cat#1864034) and following the manufacturer's instructions using EGFP-specific primers (Table S3).

Mouse studies

All animal care and experimental procedures were approved by the joint CMRI and The Children's Hospital at Westmead Animal Care and Ethics Committee.

For animals receiving intraneural AAV-CMV-EGFP, in-house bred 16-week-old male C57BL/6J mice were used. Protocols were approved by the Animal Care Committee at the University of Calgary. All applicable international, national, and institutional guidelines for the care and use of animals were followed. Briefly, mice were deeply anesthetized using 2% isoflurane with oxygen, the surgical area was shaved and disinfected with isopropanol and betadine. Buprenorphine (0.05 mg/kg subcutaneously [s.c.]) was given 15 min prior to surgery. Sciatic nerve was exposed at mid-thigh level and crushed twice with a pair of number 5 forceps for 10 s each. The nerve was then injected with 3 μL AAVs, AAV-DJ, Pep2hSC1, or Pep2hSC2 using a 5-μL Hamilton syringe fitted with a 33G needle. The crush site was landmarked with a 10-0 suture. The AAVs were delivered proximal as well as distal to the crushed site to determine the susceptibility of normal and injured SCs. After the injection, the wound was closed with 6-0 Prolene sutures and animals returned to their home cages on a heating pad for recovery. Analgesics Metacam (1 mg/kg, s.c.) was given once daily for 2 days after surgery. Nerves were harvested one month after surgery. Briefly, animals were deeply anesthetized with 5% isoflurane followed by intraperitoneal injection of overdosed sodium pentobarbital. The sciatic nerve was re-exposed and harvested and processed as described below.

CMRI's established FRG mouse colony[26] was used to breed recipient animals. FRG mice were housed in individually ventilated cages with 2-(2-nitro-4-trifluoro-methylbenzoyl)-1,3-cyclohexanedione (NTBC) supplemented in drinking water (8 mg/mL). FRG mice, 6–8 weeks old, were engrafted with human hepatocytes (Lonza Group Ltd.) as described previously.[26] Levels of human hepatocyte engraftment in chimeric mice were estimated by measuring the presence of human albumin in peripheral blood, using the human albumin ELISA quantitation kit (Bethyl Laboratories, Cat# E80-129). hFRG mice were placed on 10% NTBC before transduction with vectors and were maintained on 10% NTBC until harvest. Detailed information on all mice used in the study, including individual estimated repopulation, can be found in Table S2. We injected 2×10^{11} vgs of each AAV variant intravenously (lateral tail vein) into hFRG mice. Mice were euthanized 2 weeks after injection, with one liver lobe collected for IHC analysis prior to liver perfusion. Hepatocytes for flow cytometry analysis were obtained by collagenase perfusion of the liver as previously described with minor modifications.[53] To distinguish between mouse liver cells and human hepatocytes, cells were labeled with biotin-conjugated anti-human-HLA-ABC (eBioscience, Cat#13-9983-82, 1:100), phycoerythrin-conjugated anti-mouse-H-2K^b (BD Pharmigen, Cat#553570; 1:100), and allophycocyanin-conjugated streptavidin (eBioscience, Cat#17-4317-82, 1:500). GFP-positive-labeled samples were sorted to a minimal 95% purity using a BD AriaIII cell sorter. Flow cytometry was performed in the Flow Cytometry Facility, Westmead Institute for Medical Research. The data were analyzed using FlowJo 7.6.1 (FlowJo, LLC).

DNA and RNA extraction from cells

DNA and RNA were isolated from the cell pellets from the *in vitro* experiments using the AllPrep DNA/RNA Mini Kit (Qiagen, Cat#80204) following the manufacturer's instructions.

DNA isolation from human hepatocytes

Isolation of DNA was performed, as described in detail before without modifications.[20] Briefly, we extracted DNA using a standard phenol: chloroform protocol after proteinase K digest and RNase A digestion step.

Reverse transcription of extracted RNA

Five hundred nanograms of total RNA was treated with TURBO DNase (Invitrogen, Cat#AM2238) following the manufacturer's instructions. The DNase-treated RNA was then used for cDNA synthesis using SuperScript IV First-Strand Synthesis System (Invitrogen, Cat#18091050) following the manufacturer's instructions using 2 μM of a WPRE-binding primer (Table S3) to specifically synthesize AAV encoded transgene cDNA for barcoded NGS analysis or 2 μM of local codon-optimized AAV capsid reverse primer (lco2/lco9-NGS_R) for peptide coding region recovery in the FT-RNA library selection.

Barcode amplification, NGS, and distribution analysis

Isolation of DNA and RNA and cDNA synthesis was performed, as described in detail before[35] without modifications. Briefly, the barcoded region was amplified from 50 ng of extracted total genomic and vector DNA as well as 3 μL final cDNA product with one of five BC_F forward primers (barcoded to allow multiplexing of different samples) and the universal reverse BC_R primers using the Q5 high-fidelity DNA polymerase (NEB, Cat#M0491L). NGS reads from the DNA and cDNA populations were normalized to the reads from the respective mixes of the vectors. Heatmaps were generated using the 'pHeatmap' (version 1.0.12) source code in R Studio. DNA/cDNA NGS reads were normalized to the NGS reads prior to injection and scaled using the 'scale' function in R Studio. Clustering was performed according to the Euclidean distance between scaled values.

Vector DNA copy number per cell

Vector copy numbers were measured via ddPCR using EvaGreen supermix and following the manufacturer's instructions. To detect AAV genomes, EGFP primers were used, and vector genomes were normalized to either human or mouse albumin copy number using primers hALB_F/R for cells from human origin as well as mALB_F/R for cells from mouse origin (Table S3).

Heparin competition assay on HEK293T cells

We seeded 1.5×10^5 HEK293T cells per well (24-well format) 24 h before transduction in culture medium (DMEM supplemented with 10% FBS and 1% P/S). Cells were transduced with or without the presence of 500 mg/mL heparin. Percentage of transgene-expressing cells was determined by flow cytometry 72 h after transduction.

Neutralization assay

Briefly, 1.5×10^5 HEK293T cells per well (24-well format) were seeded 24 h before transduction. Vectors diluted in DMEM medium (supplemented with 10% FBS and 1% P/S) were incubated for 1 h at 37°C with undiluted (neat), 1:2 diluted, 1:4 diluted, 1:16 diluted, and 1:32 diluted human IVIg (Intragam 10, 10g/100 mL, CSL Behring). The mixture of vector and serum diluted in medium was added onto the cells and incubated for 48 h at 37°C and 5% CO_2. Transduction efficiency in the presence of IVIg was normalized to the vector performance in the absence of IVIg (performed in parallel) and was analyzed by flow cytometry by determining the percentage of EGFP-positive cells.

In vitro AAV transduction and NGS

Mouse, rat, or human SCs were seeded on a poly-D-lysine and laminin-coated six-well plates using the culture conditions indicated above. Fibroblast were seeded at 300,000 cells per well in six-well plates. The cells were incubated overnight with the AAV testing kit or the hSC testing kit. After PBS wash, fresh medium was provided, and all cells were allowed to grow for an additional 32 h before harvest. Cells were harvested by incubating with TrypLE Express (Gibco, Cat#12604021) for 5 min at 37°C. The cells were then recovered in fresh media, spun at 300×*g* for 5 min, and the dry pellet was used for DNA and RNA extraction.

In vitro AAV transduction and flow cytometry

Mouse, rat, or human SCs were seeded on a poly-D-lysine and laminin-coated 12-well plate using the culture conditions indicated above. Fibroblast were seeded at 300,000 cells per well in six-well plates. The cells were incubated overnight with respective AAV vector variants at 1,000 vg per cell.

After 16 h, cells were then washed, and media were then replaced. Three days after exposure to the vector, the cells were rinsed once with PBS (Gibco, Cat#14190144), dissociated using TrypLE Express, and recovered in their culture media. Following transfer into 5-mL polystyrene tubes, the cells were spun down at 300×*g* for 5 min and resuspended in FACS buffer (PBS, 2% FBS, 5 mM EDTA) (Invitrogen, Cat#15575-020). The flow cytometry analysis was performed using a BD FACSCanto cell analyzer. The flow cytometry data was analyzed with Flow Jo 7.6.1.

AAV transduction of primary SC cultures and immunofluorescence

Rat or human SCs were seeded into 24-well plates containing poly-D-lysine and laminin-coated coverslips plates and allowed to adhere overnight. SCs were transduced with rAAVs packaged using candidate capsids at 1,000 vg/cell. After 16 h, cells were then washed and media were then replaced. EGFP expression was assessed by fluorescence microscopy (Zeiss Axio Imager.M1) 3 days after AAV exposure.

AAV transduction of human nerve explants and immunofluorescence

For the human nerve explants, individual nerve fascicles were pulled from autopsied sciatic nerve and cut into 1-cm nerve segments. AAV diluted in PBS/5% sucrose (1.1×10^{10} GC) was injected into the fascicles using a 5-μL Hamilton syringe fitted with a 33G needle, the nerve was trimmed to the 0.5-cm segment with maximal bolus retention. Individual nerve explants were then placed separately in 24-well plates for 14 days in DMEM with 10% FBS, 1% P/S supplemented with 50 ng/mL NRG1 and 5 μM Fsk in a humidified incubator at 5% CO_2 and 37°C. Media were changed twice weekly.

Immunohistochemistry

For immunostaining of chimeric liver samples, one lobe was collected and was fixed with 4% (w/v in PBS) paraformaldehyde (PFA) overnight at 4°C before being cryoprotected through a sucrose gradient (10%, 20% and 30% w/v sucrose in PBS). Liver samples were then frozen in optimal cutting temperature (OCT) (Tissue-Tek; Sakura Finetek USA). Liver sections (5 μm) were prepared on a Cryostat (Leica, Cat# CM1950). Sections were permeabilized with 0.2% Triton X-100 in PBS (PBST) and blocked in 10% rabbit serum (Sigma Aldrich) in PBS for 30 min at room temperature.

Sections were then incubated with rabbit monoclonal anti-human GAPDH antibody conjugated with Alexa Fluor 647 (Abcam, Cat#ab215227, clone AF674, 1:600 dilution) at room temperature

for 2 h. Histological analysis using immunofluorescence microscopy was performed using a Zeiss Axio Imager.M1 with ZEN 2 software.

For immunostaining of the sciatic nerve, sciatic nerve was dissected, immerse-fixed overnight in 4% PFA in 0.1 M phosphate buffer (PB) at 4°C. The specimens were then cryoprotected in 30% sucrose in PB solution at 4°C until the specimens had sunk. They were then embedded in optimal cutting temperature and sectioned on a cryostat at a thickness of 10 μm. Sections were collected on SuperFrost Plus slides (VWR). Sections were permeabilized and blocked with 10% normal serum, in PBST solution containing 0.3% Triton X-100 for 1 h at room temperature. Sections were then incubated with primary antibodies including rabbit anti-CD271 (p75ntr) (BioLegend, Cat# 839701, clone Poly18397, 1:1,000), rabbit anti-NF 200 (Sigma, Cat#N4142, 1:500), mouse anti-PRX (Novus, Cat#NBP-1-89598, 1:500), and rabbit anti-S100β (Dako, Cat#Z0311, 1:1,000) in diluent at 4°C overnight. Sections were washed in PBST buffer, then labeled with corresponding secondary antibodies for 1 h at room temperature. After washing with PBS, sections were mounted with PermaFluor mounting medium (Thermo Fisher Scientific), and imaged with a slide scanner (VS110, Olympus, Japan).

For cultured SCs, cells were first washed in cold PBS and then fixed in 4% PFA for 10–15 min at 25°C. After washing three times in PBS, cells were permeabilized in 0.2% PBST and blocked in 10% goat serum in PBST at 25°C for 30 min. Staining was performed with an FLEX rabbit polyclonal S100 antibody (Dako, Cat#GA50461-2) overnight at 4°C. Cells were washed three times in PBS and incubated with a secondary antibody Alexa Fluor Plus 594 (Thermo Fisher Scientific, Cat#A-11012, 1:500) at 25°C for 1 h. Cells were then washed in PBS and counterstained with DAPI (Invitrogen, Cat#D1306) at 0.08 ng/mL.

Statistics and reproducibility

Experimenters were not blinded for any of the studies performed in this manuscript. All data are presented as mean ± SEM. Statistical significance was assessed using GraphPad Prism 9 software. The test used is specified in figure legends. A p value of 0.05 or less was considered significant in all experiments.

DATA AND CODE AVAILABILITY

All data generated or analyzed during this study are included in this published article.

SUPPLEMENTAL INFORMATION

Supplemental information can be found online at https://doi.org/10.1016/j.omtm.2024.101234.

ACKNOWLEDGMENTS

The authors thank CMRI Vector and Genome Engineering Facility (VGEF) for help in vector preparation. The authors also thank the Imaging Facility of CMRI. We also thank the Cytometry Facility of the Westmead Institute for Medical Research for help with sorting of human hepatocytes. We also would like to thank all the members of the CMRI Bioresources, with special thanks to S. Dimech. We also thank Prof. Margaret Wallace (University of Florida) for providing primary human SCs. Figure 5A was generated with BioRender (https://biorender.com). This research was supported by NSW Health Early-Mid Career Gene and Cell Therapy Grants Program (grant K5126/RD665 to M.D.). This work was supported by Children's Tumor Foundation (CTF) grant award to J.B., S.L.G., and L.L.

AUTHOR CONTRIBUTIONS

M.D, S.L.G.; and L.L. designed the study. M.D., T-H.C., F.H., R.G.N., E.Z., A.W., M.C.-C., N.R., D.N., and E.L. performed experiments. R.G.N. and E.Z. assisted in hFRG experiments (injections and harvesting). T-H.C. carried out mouse intraneural experiments and *ex vivo* human nerve experiments. D.N. participated in AAV vector production. A.W. generated the AAV peptide libraries. F.H. participated in *in vitro* experiments on primary human Schwann cells. T-H.C., N.R., and E.L. performed *in vitro* experiments on primary human Schwann cells and fibroblasts at University of Calgary, Calgary. S.S. developed NGS analysis script and assisted with the bioinformatic analysis. J.M. helped with figure generation. M.D. prepared the figures with input from all authors. M.D., S.L.G., T-H.C., and L.L. analyzed data. M.D., S.L.G., J.B., I.E.A., and L.L. supervised all aspects of the work. M.D, S.L.G., and L.L wrote the manuscript.
All authors have reviewed the manuscript.

DECLARATION OF INTERESTS

M.D., J.M., M.C.-C., A.W., S.L.G., I.E.A., and L.L. are inventors on patent applications filed by Children's Medical Research Institute related to AAV capsid sequences, *in vivo* function of novel AAV variants and AAV selection platforms. L.L. is a co-founder and scientific advisor of LogicBio Therapeutics. L.L. and I.A.E. are co-founders of Exigen Biotherapeutics. L.L. and I.E.A. have consulted on broad technologies addressed in this paper. L.L. and I.A.E. have stock and/or equity in companies with technology broadly related to this paper.

REFERENCES

1. Mendell, J.R., Al-Zaidy, S., Shell, R., Arnold, W.D., Rodino-Klapac, L.R., Prior, T.W., Lowes, L., Alfano, L., Berry, K., Church, K., et al. (2017). Single-Dose Gene-Replacement Therapy for Spinal Muscular Atrophy. N. Engl. J. Med. *377*, 1713–1722. https://doi.org/10.1056/NEJMoa1706198.

2. Ginn, S.L., Amaya, A.K., Alexander, I.E., Edelstein, M., and Abedi, M.R. (2018). Gene therapy clinical trials worldwide to 2017: An update. J. Gene Med. *20*, e3015. https://doi.org/10.1002/jgm.3015.

3. Au, H.K.E., Isalan, M., and Mielcarek, M. (2021). Gene Therapy Advances: A Meta-Analysis of AAV Usage in Clinical Settings. Front. Med. *8*, 809118. https://doi.org/10.3389/fmed.2021.809118.

4. Büning, H., and Srivastava, A. (2019). Capsid Modifications for Targeting and Improving the Efficacy of AAV Vectors. Mol. Ther. Methods Clin. Dev. *12*, 248–265. https://doi.org/10.1016/j.omtm.2019.01.008.

5. Bhatheja, K., and Field, J. (2006). Schwann cells: Origins and role in axonal maintenance and regeneration. Int. J. Biochem. Cell Biol. *38*, 1995–1999. https://doi.org/10.1016/j.biocel.2006.05.007.

6. O'Carroll, S.J., Cook, W.H., and Young, D. (2020). AAV Targeting of Glial Cell Types in the Central and Peripheral Nervous System and Relevance to Human Gene Therapy. Front. Mol. Neurosci. *13*, 618020. https://doi.org/10.3389/fnmol.2020.618020.

7. Jessen, K.R., and Mirsky, R. (2016). The repair Schwann cell and its function in regenerating nerves. J. Physiol. *594*, 3521–3531. https://doi.org/10.1113/JP270874.

8. Wang, Q., Chen, F.-Y., Ling, Z.-M., Su, W.-F., Zhao, Y.-Y., Chen, G., and Wei, Z.-Y. (2022). The Effect of Schwann Cells/Schwann Cell-Like Cells on Cell Therapy for Peripheral Neuropathy. Front. Cell. Neurosci. *16*, 836931. https://doi.org/10.3389/fncel.2022.836931.

9. Sargiannidou, I., Kagiava, A., and Kleopa, K.A. (2020). Gene therapy approaches targeting Schwann cells for demyelinating neuropathies. Brain Res. *1728*, 146572. https://doi.org/10.1016/j.brainres.2019.146572.

10. Bai, R.-Y., Esposito, D., Tam, A.J., McCormick, F., Riggins, G.J., Wade Clapp, D., and Staedtke, V. (2019). Feasibility of using NF1-GRD and AAV for gene replacement therapy in NF1-associated tumors. Gene Ther. *26*, 277–286. https://doi.org/10.1038/s41434-019-0080-9.

11. Prabhakar, S., Beauchamp, R.L., Cheah, P.S., Yoshinaga, A., Haidar, E.A., Lule, S., Mani, G., Maalouf, K., Stemmer-Rachamimov, A., Jung, D.H., et al. (2022). Gene replacement therapy in a schwannoma mouse model of neurofibromatosis type 2. Mol. Ther. Methods Clin. Dev. *26*, 169–180. https://doi.org/10.1016/j.omtm.2022.06.012.

12. Schiza, N., Georgiou, E., Kagiava, A., Médard, J.J., Richter, J., Tryfonos, C., Sargiannidou, I., Heslegrave, A.J., Rossor, A.M., Zetterberg, H., et al. (2019). Gene replacement therapy in a model of Charcot-Marie-Tooth 4C neuropathy. Brain *142*, 1227–1241. https://doi.org/10.1093/brain/awz064.

13. Kagiava, A., Karaiskos, C., Richter, J., Tryfonos, C., Jennings, M.J., Heslegrave, A.J., Sargiannidou, I., Stavrou, M., Zetterberg, H., Reilly, M.M., et al. (2021). AAV9-mediated Schwann cell-targeted gene therapy rescues a model of demyelinating neuropathy. Gene Ther. *28*, 659–675. https://doi.org/10.1038/s41434-021-00250-0.

14. Gautier, B., Hajjar, H., Soares, S., Berthelot, J., Deck, M., Abbou, S., Campbell, G., Ceprian, M., Gonzalez, S., Fovet, C.-M., et al. (2021). AAV2/9-mediated silencing of PMP22 prevents the development of pathological features in a rat model of Charcot-Marie-Tooth disease 1 A. Nat. Commun. *12*, 2356. https://doi.org/10.1038/s41467-021-22593-3.

15. Srivastava, A. (2016). In vivo tissue-tropism of adeno-associated viral vectors. Curr. Opin. Virol. *21*, 75–80. https://doi.org/10.1016/j.coviro.2016.08.003.

16. Hoyng, S.A., de Winter, F., Gnavi, S., van Egmond, L., Attwell, C.L., Tannemaat, M.R., Verhaagen, J., and Malessy, M.J.A. (2015). Gene delivery to rat and human Schwann cells and nerve segments: a comparison of AAV 1–9 and lentiviral vectors. Gene Ther. *22*, 767–780. https://doi.org/10.1038/gt.2015.47.

17. Zincarelli, C., Soltys, S., Rengo, G., and Rabinowitz, J.E. (2008). Analysis of AAV Serotypes 1–9 Mediated Gene Expression and Tropism in Mice After Systemic Injection. Mol. Ther. *16*, 1073–1080. https://doi.org/10.1038/mt.2008.76.

18. Lisowski, L., Dane, A.P., Chu, K., Zhang, Y., Cunningham, S.C., Wilson, E.M., Nygaard, S., Grompe, M., Alexander, I.E., and Kay, M.A. (2014). Selection and evaluation of clinically relevant AAV variants in a xenograft liver model. Nature *506*, 382–386. https://doi.org/10.1038/nature12875.

19. Westhaus, A., Cabanes Creus, M., Jonker, T., Sallard, E., Navarro, R.G., Zhu, E., Baltazar, G., Lee, S., Wilmott, P., Gonzalez-Cordero, A., et al. (2022). AAV-p40 bioengineering platform for variant selection based on transgene expression. Hum. Gene Ther. *33*, 664–682. https://doi.org/10.1089/hum.2021.278.

20. Westhaus, A., Cabanes-Creus, M., Rybicki, A., Baltazar, G., Navarro, R.G., Zhu, E., Drouyer, M., Knight, M., Albu, R.F., Ng, B.H., et al. (2020). High-Throughput *In Vitro* , *Ex Vivo,* and *In Vivo* Screen of Adeno-Associated Virus Vectors Based on Physical and Functional Transduction. Hum. Gene Ther. *31*, 575–589. https://doi.org/10.1089/hum.2019.264.

21. Chu, T.-H., Baral, K., Labit, E., Rosin, N., Sinha, S., Umansky, D., Alzahrani, S., Arora, R., Cao, L., Rancourt, D., et al. (2022). Comparison of human skin- and nerve-derived Schwann cells reveals many similarities and subtle genomic and functional differences. Glia *70*, 2131–2156. https://doi.org/10.1002/glia.24242.

22. Cabanes Creus, M. (2019). Novel AAV Engineering Technology: Identification of Improved AAV Variants for Gene Addition and Genome Engineering in Primary Human Cells (Institute of Child Health, University College London), p. 255.

23. Pekrun, K., de Alencastro, G., Luo, Q.-J., Liu, J., Kim, Y., Nygaard, S., Galivo, F., Zhang, F., Song, R., Tiffany, M.R., et al. (2019). Using a barcoded AAV capsid library to select for clinically relevant gene therapy vectors. JCI Insight *4*, e131610. https://doi.org/10.1172/jci.insight.131610.

24. Opie, S.R., Warrington, K.H., Agbandje-McKenna, M., Zolotukhin, S., and Muzyczka, N. (2003). Identification of amino acid residues in the capsid proteins of adeno-associated virus type 2 that contribute to heparan sulfate proteoglycan binding. J. Virol. *77*, 6995–7006. https://doi.org/10.1128/jvi.77.12.6995-7006.2003.

25. Kern, A., Schmidt, K., Leder, C., Müller, O.J., Wobus, C.E., Bettinger, K., von der Lieth, C.W., King, J.A., and Kleinschmidt, J.A. (2003). Identification of a heparin-binding motif on adeno-associated virus type 2 capsids. J. Virol. *77*, 11072–11081. https://doi.org/10.1128/jvi.77.20.11072-11081.2003.

26. Azuma, H., Paulk, N., Ranade, A., Dorrell, C., Al-Dhalimy, M., Ellis, E., Strom, S., Kay, M.A., Finegold, M., and Grompe, M. (2007). Robust expansion of human hepatocytes in Fah−/−/Rag2−/−/Il2rg−/− mice. Nat. Biotechnol. *25*, 903–910. https://doi.org/10.1038/nbt1326.

27. Sugahara, G., Ishida, Y., Sun, J., Tateno, C., and Saito, T. (2020). Art of Making Artificial Liver: Depicting Human Liver Biology and Diseases in Mice. Semin. Liver Dis. *40*, 189–212. https://doi.org/10.1055/s-0040-1701444.

28. Zhu, Y., Ghosh, P., Charnay, P., Burns, D.K., and Parada, L.F. (2002). Neurofibromas in NF1: Schwann Cell Origin and Role of Tumor Environment. Science *296*, 920–922. https://doi.org/10.1126/science.1068452.

29. Ma, D., Wang, B., Zawadzka, M., Gonzalez, G., Wu, Z., Yu, B., Rawlins, E.L., Franklin, R.J.M., and Zhao, C. (2018). A Subpopulation of Foxj1-Expressing, Nonmyelinating Schwann Cells of the Peripheral Nervous System Contribute to Schwann Cell Remyelination in the Central Nervous System. J. Neurosci. *38*, 9228–9239. https://doi.org/10.1523/JNEUROSCI.0585-18.2018.

30. Chen, Z., Mo, J., Brosseau, J.-P., Shipman, T., Wang, Y., Liao, C.-P., Cooper, J.M., Allaway, R.J., Gosline, S.J.C., Guinney, J., et al. (2019). Spatiotemporal Loss of NF1 in Schwann Cell Lineage Leads to Different Types of Cutaneous Neurofibroma Susceptible to Modification by the Hippo Pathway. Cancer Discov. *9*, 114–129. https://doi.org/10.1158/2159-8290.CD-18-0151.

31. Li, C., and Samulski, R.J. (2020). Engineering adeno-associated virus vectors for gene therapy. Nat. Rev. Genet. *21*, 255–272. https://doi.org/10.1038/s41576-019-0205-4.

32. Goertsen, D., Flytzanis, N.C., Goeden, N., Chuapoco, M.R., Cummins, A., Chen, Y., Fan, Y., Zhang, Q., Sharma, J., Duan, Y., et al. (2022). AAV capsid variants with brain-wide transgene expression and decreased liver targeting after intravenous delivery in mouse and marmoset. Nat. Neurosci. *25*, 106–115. https://doi.org/10.1038/s41593-021-00969-4.

33. Nonnenmacher, M., Wang, W., Child, M.A., Ren, X.-Q., Huang, C., Ren, A.Z., Tocci, J., Chen, Q., Bittner, K., Tyson, K., et al. (2021). Rapid evolution of blood-brain-barrier-penetrating AAV capsids by RNA-driven biopanning. Mol. Ther. Methods Clin. Dev. *20*, 366–378. https://doi.org/10.1016/j.omtm.2020.12.006.

34. Öztürk, B.E., Johnson, M.E., Kleyman, M., Turunç, S., He, J., Jabalameli, S., Xi, Z., Visel, M., Dufour, V.L., Iwabe, S., et al. (2021). scAAVengr, a transcriptome-based pipeline for quantitative ranking of engineered AAVs with single-cell resolution. Elife *10*, e64175. https://doi.org/10.7554/eLife.64175.

35. Cabanes-Creus, M., Navarro, R.G., Zhu, E., Baltazar, G., Liao, S.H.Y., Drouyer, M., Amaya, A.K., Scott, S., Nguyen, L.H., Westhaus, A., et al. (2022). Novel human liver-tropic AAV variants define transferable domains that markedly enhance the human tropism of AAV7 and AAV8. Mol. Ther. Methods Clin. Dev. *24*, 88–101. https://doi.org/10.1016/j.omtm.2021.11.011.

36. Meumann, N., Cabanes-Creus, M., Ertelt, M., Navarro, R.G., Lucifora, J., Yuan, Q., Nien-Huber, K., Abdelrahman, A., Vu, X.K., Zhang, L., et al. (2023). Adeno-associated virus serotype 2 capsid variants for improved liver-directed gene therapy. Hepatology *77*, 802–815. https://doi.org/10.1002/hep.32733.

37. Tabebordbar, M., Lagerborg, K.A., Stanton, A., King, E.M., Ye, S., Tellez, L., Krunnfusz, A., Tavakoli, S., Widrick, J.J., Messemer, K.A., et al. (2021). Directed evolution of a family of AAV capsid variants enabling potent muscle-directed gene delivery across species. Cell *184*, 4919–4938.e22. https://doi.org/10.1016/j.cell.2021.08.028.

38. Messing, A., Behringer, R.R., Hammang, J.P., Palmiter, R.D., Brinster, R.L., and Lemke, G. (1992). P0 promoter directs expression of reporter and toxin genes to Schwann cells of transgenic mice. Neuron *8*, 507–520. https://doi.org/10.1016/0896-6273(92)90279-m.

39. Kagiava, A., Richter, J., Tryfonos, C., Leal-Julià, M., Sargiannidou, I., Christodoulou, C., Bosch, A., and Kleopa, K.A. (2021). Efficacy of AAV serotypes to target Schwann cells after intrathecal and intravenous delivery. Sci. Rep. *11*, 23358. https://doi.org/10.1038/s41598-021-02694-1.

40. Scherer, S.S., Xu, Y.-T., Messing, A., Willecke, K., Fischbeck, K.H., and Jeng, L.J.B. (2005). Transgenic Expression of Human *Connexin32* in Myelinating Schwann Cells Prevents Demyelination in *Connexin32* -Null Mice. J. Neurosci. *25*, 1550–1559. https://doi.org/10.1523/JNEUROSCI.3082-04.2005.

41. Stratton, J.A., Kumar, R., Sinha, S., Shah, P., Stykel, M., Shapira, Y., Midha, R., and Biernaskie, J. (2017). Purification and Characterization of Schwann Cells from Adult Human Skin and Nerve. eNeuro *4*. ENEURO.0307-16.2017. https://doi.org/10.1523/ENEURO.0307-16.2017.

42. Feldman, A.G., Parsons, J.A., Dutmer, C.M., Veerapandiyan, A., Hafberg, E., Maloney, N., and Mack, C.L. (2020). Subacute Liver Failure Following Gene Replacement Therapy for Spinal Muscular Atrophy Type 1. J. Pediatr. *225*, 252–258.e1. https://doi.org/10.1016/j.jpeds.2020.05.044.

43. Harrison, C. (2020). High-dose AAV gene therapy deaths. Nat. Biotechnol. *38*, 910. https://doi.org/10.1038/s41587-020-0642-9.

44. Morales, L., Gambhir, Y., Bennett, J., and Stedman, H.H. (2020). Broader Implications of Progressive Liver Dysfunction and Lethal Sepsis in Two Boys following Systemic High-Dose AAV. Mol. Ther. *28*, 1753–1755. https://doi.org/10.1016/j.ymthe.2020.07.009.

45. Qiao, C., Yuan, Z., Li, J., He, B., Zheng, H., Mayer, C., Li, J., and Xiao, X. (2011). Liver-specific microRNA-122 target sequences incorporated in AAV vectors efficiently inhibits transgene expression in the liver. Gene Ther. *18*, 403–410. https://doi.org/10.1038/gt.2010.157.

46. Xie, J., Ameres, S.L., Friedline, R., Hung, J.-H., Zhang, Y., Xie, Q., Zhong, L., Su, Q., He, R., Li, M., et al. (2012). Long-term, efficient inhibition of microRNA function in mice using rAAV vectors. Nat. Methods *9*, 403–409. https://doi.org/10.1038/nmeth.1903.

47. Xie, J., Xie, Q., Zhang, H., Ameres, S.L., Hung, J.-H., Su, Q., He, R., Mu, X., Seher Ahmed, S., Park, S., et al. (2011). MicroRNA-regulated, Systemically Delivered rAAV9: A Step Closer to CNS-restricted Transgene Expression. Mol. Ther. *19*, 526–535. https://doi.org/10.1038/mt.2010.279.

48. Hordeaux, J., Wang, Q., Katz, N., Buza, E.L., Bell, P., and Wilson, J.M. (2018). The Neurotropic Properties of AAV-PHP.B Are Limited to C57BL/6J Mice. Mol. Ther. *26*, 664–668. https://doi.org/10.1016/j.ymthe.2018.01.018.

49. Beharry, A., Gong, Y., Kim, J.C., Hanlon, K.S., Nammour, J., Hieber, K., Eichler, F., Cheng, M., Stemmer-Rachamimov, A., Stankovic, K.M., et al. (2022). The AAV9 Variant Capsid AAV-F Mediates Widespread Transgene Expression in Nonhuman Primate Spinal Cord After Intrathecal Administration. Hum. Gene Ther. *33*, 61–75. https://doi.org/10.1089/hum.2021.069.

50. Gray, S.J., Nagabhushan Kalburgi, S., McCown, T.J., and Jude Samulski, R. (2013). Global CNS gene delivery and evasion of anti-AAV-neutralizing antibodies by intrathecal AAV administration in non-human primates. Gene Ther. *20*, 450–459. https://doi.org/10.1038/gt.2012.101.

51. Cabanes-Creus, M., Ginn, S.L., Amaya, A.K., Liao, S.H.Y., Westhaus, A., Hallwirth, C.V., Wilmott, P., Ward, J., Dilworth, K.L., Santilli, G., et al. (2019). Codon-Optimization of Wild-Type Adeno-Associated Virus Capsid Sequences Enhances DNA Family Shuffling while Conserving Functionality. Mol. Ther. Methods Clin. Dev. *12*, 71–84. https://doi.org/10.1016/j.omtm.2018.10.016.

52. Khan, I.F., Hirata, R.K., and Russell, D.W. (2011). AAV-mediated gene targeting methods for human cells. Nat. Protoc. *6*, 482–501. https://doi.org/10.1038/nprot.2011.301.

53. Cabanes-Creus, M., Hallwirth, C.V., Westhaus, A., Ng, B.H., Liao, S.H.Y., Zhu, E., Navarro, R.G., Baltazar, G., Drouyer, M., Scott, S., et al. (2020). Restoring the natural tropism of AAV2 vectors for human liver. Sci. Transl. Med. *12*. eaba3312–13. https://doi.org/10.1126/scitranslmed.aba3312.

OMTM, Volume *32*

Supplemental information

Novel AAV variants with improved tropism for human Schwann cells

Matthieu Drouyer, Tak-Ho Chu, Elodie Labit, Florencia Haase, Renina Gale Navarro, Deborah Nazareth, Nicole Rosin, Jessica Merjane, Suzanne Scott, Marti Cabanes-Creus, Adrian Westhaus, Erhua Zhu, Rajiv Midha, Ian E. Alexander, Jeff Biernaskie, Samantha L. Ginn, and Leszek Lisowski

Supplemental material

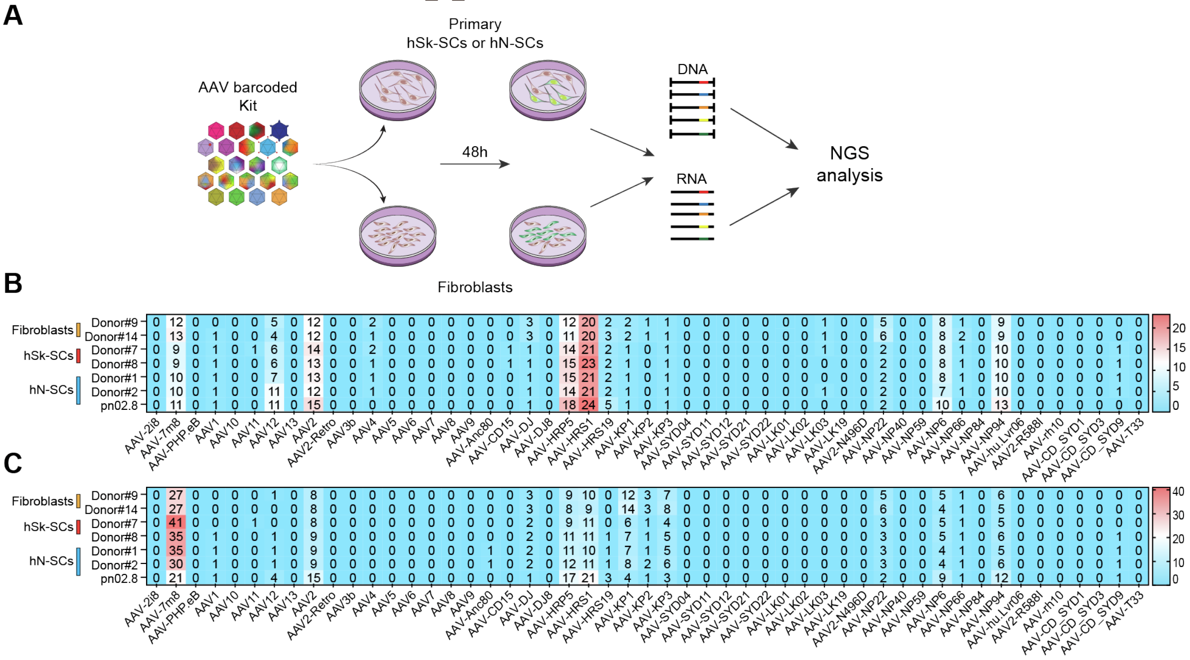

Figure S1. Performance of pooled AAV variants in primary hSCs and in fibroblasts. (A) Graphical overview of the AAV testing kit process *in vitro*. (B and C) The use of the pooled 51-variant "AAV Testing Kit" allows for simultaneous analysis of barcoded variants with capsid recovery achieved at the level of both (B) cell entry (DNA) and (C) transgene expression (mRNA). Heat-map indicates the performance of each capsid as a percentage of total NGS reads per cell type. Values are normalized to the 'pre-mix pool'.

DNA_human SCs

Donor#11	Donor#12	Donor#13	Donor#14	Donor#4	Donor#3	pn02.8	Variant	Average
11.860	8.610	9.000	7.260	8.200	8.770	2.964	Pep2hSC2	8.095
11.420	7.790	7.040	6.390	6.870	7.390	4.216	Pep2hSC1	7.302
5.950	4.410	4.110	4.710	5.630	5.040	4.056	AAV-HRP5	4.844
5.070	4.430	4.140	4.350	4.880	4.840	3.749	AAV2	4.494
4.500	4.270	3.990	4.200	4.810	4.230	3.431	AAV-NP6	4.204
4.530	4.630	3.800	4.160	4.600	4.200	3.470	Shuffle35	4.199
4.360	4.040	3.940	4.100	4.530	4.230	3.400	AAV-NP94	4.086
4.420	4.150	3.790	3.950	4.430	4.160	3.447	AAV-HRS1	4.050
4.420	3.900	3.970	3.460	3.500	3.810	3.790	Pep9hSC5	3.836
4.040	3.910	3.710	3.780	3.960	3.920	3.302	Shuffle9	3.803
1.630	2.880	3.010	3.700	6.950	2.940	3.711	Pep9hSC2	3.546
2.810	3.720	4.050	3.850	3.930	3.590	2.093	AAV-7m8	3.435
3.040	3.210	3.130	3.180	3.740	3.210	2.666	Shuffle32	3.168
2.660	3.190	3.450	3.080	2.160	3.240	3.972	Pep9hSC9	3.107
1.710	2.390	2.490	2.700	3.810	2.350	3.621	Pep9hSC1	2.724
3.730	2.350	2.440	2.210	2.550	2.460	3.190	PephSC10	2.704
2.710	2.870	2.700	2.830	1.520	2.680	3.031	Shuffle31	2.620
2.720	2.700	2.880	2.520	1.340	2.510	2.894	Pep2hSC5	2.509
1.780	2.340	2.420	2.150	3.330	2.120	2.444	Pep2hSC4	2.369
1.390	2.380	2.400	2.680	1.670	2.400	3.643	Pep9hSC10	2.366
1.370	2.080	2.240	3.180	0.780	2.510	3.478	Pep9hSC8	2.234
1.270	2.040	2.070	2.480	0.690	2.230	3.498	Pep9hSC7	2.040
1.120	1.740	2.050	1.520	4.030	1.370	1.175	AAV-DJ	1.858
1.870	2.020	2.290	1.930	0.410	2.340	2.137	Pep2hSC9	1.857
1.780	1.690	1.730	1.740	0.980	1.770	1.394	AAV-KP3	1.583
0.630	1.010	1.140	1.850	2.530	1.150	2.605	Pep9hSC6	1.559
1.260	1.730	2.000	1.240	1.130	1.520	1.641	PephSC8	1.503
0.660	1.370	1.360	1.930	0.840	1.450	2.845	Pep9hSC11	1.494
0.890	1.460	1.490	1.110	1.030	1.250	1.914	Pep2hSC7	1.306
1.170	1.510	1.460	1.150	0.630	0.960	2.227	Pep2hSC3	1.301
0.850	1.310	1.440	1.220	1.020	1.130	1.847	Pep2hSC6	1.260
0.510	0.980	0.920	1.590	0.690	1.040	2.644	Pep9hSC12	1.196
0.480	0.880	1.050	1.500	0.230	1.060	2.625	Pep9hSC3	1.118
0.630	0.820	1.030	0.790	0.590	0.640	0.850	AAV-NP22	0.764
0.350	0.570	0.540	1.010	0.680	0.780	1.339	Pep9hSC4	0.753
0.410	0.640	0.730	0.510	1.320	0.700	0.693	AAV-KP1	0.715

cDNA_human SCs

Donor#11	Donor#12	Donor#13	Donor#14	Donor#4	Donor#3	pn02.8	Variant	Average
18.250	11.050	7.510	19.040	15.290	20.210	9.615	Pep2hSC1	14.424
8.420	20.330	18.660	2.260	13.450	6.720	4.816	AAV-7m8	10.665
10.010	3.200	1.800	18.120	5.800	5.150	1.521	Pep2hSC2	6.514
5.590	5.500	4.970	3.120	6.150	4.690	4.827	Shuffle35	4.978
4.370	4.350	3.880	7.850	5.860	3.700	4.531	Shuffle32	4.934
3.510	8.460	11.290	1.120	4.830	2.470	1.211	AAV-KP1	4.699
3.890	3.040	3.090	7.150	3.900	3.720	4.263	Shuffle9	4.150
2.920	4.980	5.830	0.660	4.140	2.980	1.949	AAV-KP3	3.351
3.040	3.760	3.640	0.660	4.210	3.580	4.499	Shuffle31	3.341
4.280	2.440	2.340	0.830	2.950	3.770	4.766	AAV-HRP5	3.054
3.280	2.440	3.210	0.620	2.750	3.180	5.255	AAV2	2.962
3.160	2.310	3.130	1.500	2.910	3.050	3.853	AAV-NP6	2.845
3.410	2.120	1.730	1.740	2.980	4.170	2.611	Pep9hSC5	2.680
2.930	1.720	2.420	1.490	2.370	2.770	4.428	AAV-NP94	2.590
2.360	1.810	1.350	3.990	2.200	3.210	2.845	Pep9hSC9	2.538
2.800	1.680	2.530	0.760	2.430	2.910	4.643	AAV-HRS1	2.536
1.770	3.080	4.110	1.070	2.140	1.040	1.272	AAV-DJ	2.069
2.100	2.230	2.770	0.520	1.650	1.600	2.512	Pep2hSC5	1.912
1.280	1.530	1.460	3.080	1.420	2.330	2.026	Pep2hSC4	1.875
0.990	1.640	1.420	4.350	1.630	1.510	0.999	Pep2hSC8	1.791
1.160	1.040	0.950	1.430	0.990	2.040	3.235	Pep9hSC1	1.549
0.530	0.740	0.700	3.130	0.630	1.590	2.284	Pep9hSC11	1.372
0.350	0.540	0.510	0.850	0.500	1.200	2.280	Pep2hSC6	1.335
0.910	0.870	0.810	1.210	0.860	1.650	2.674	Pep9hSC2	1.283
1.130	1.760	1.440	0.640	0.950	0.800	2.055	Pep2hSC3	1.254
0.830	0.900	1.310	1.200	1.170	0.950	1.884	Pep2hSC7	1.178
0.610	0.750	0.710	1.100	0.860	1.480	2.025	Pep9hSC7	1.076
0.280	0.250	0.220	4.440	0.270	0.440	1.056	Pep9hSC3	0.994
0.840	1.620	2.260	0.010	0.910	0.530	0.773	AAV-NP22	0.992
0.580	0.600	0.530	1.290	0.560	1.250	1.571	Pep9hSC8	0.912
1.610	0.350	0.290	1.080	0.760	0.740	1.443	PephSC10	0.896
0.940	1.320	1.660	1.460	0.880	1.350	1.737	Pep9hSC6	0.890
0.680	0.460	0.390	1.090	0.650	0.900	0.849	Pep9hSC10	0.717
0.700	0.560	0.560	0.220	0.510	0.890	1.491	Pep2hSC9	0.704
0.290	0.360	0.300	0.580	0.310	0.760	1.391	Pep9hSC12	0.570
0.190	0.220	0.230	0.370	0.150	0.670	0.808	Pep9hSC4	0.377

DNA_Fibroblasts

F-Donor#10	F-Donor#6	F-Donor#5	Variant	Average
5.830	6.120	6.380	AAV2	6.110
5.640	5.710	5.720	AAV-7m8	5.690
5.400	5.770	5.820	AAV-NP94	5.663
5.200	5.610	5.880	Shuffle35	5.563
5.280	5.520	5.850	AAV-NP6	5.550
6.180	5.390	4.550	Pep2hSC5	5.373
4.940	5.340	5.660	Shuffle9	5.313
4.900	5.370	5.560	AAV-HRS1	5.277
4.390	4.530	4.750	AAV-HRP5	4.557
5.380	4.830	2.940	AAV-DJ	4.383
4.100	4.340	4.460	Shuffle31	4.300
3.890	4.160	4.620	Shuffle32	4.223
3.900	3.870	3.520	Pep2hSC4	3.763
3.390	3.590	3.820	Pep2hSC10	3.600
3.480	3.250	2.920	Pep2hSC9	3.217
3.600	2.710	2.440	Pep2hSC8	2.917
2.330	2.600	3.630	Pep2hSC1	2.853
2.710	2.600	1.960	Pep2hSC7	2.423
2.510	2.430	2.240	AAV-KP3	2.393
1.930	2.120	3.050	Pep2hSC2	2.367
2.400	2.160	2.140	Pep2hSC3	2.233
2.680	2.070	1.220	AAV-NP22	1.990
2.360	1.660	1.010	AAV-KP1	1.677
1.800	1.690	1.200	Pep2hSC6	1.563
1.180	1.170	1.640	Pep9hSC5	1.330
0.920	1.040	1.250	Pep9hSC9	1.070
0.540	0.670	0.930	Pep9hSC2	0.713
0.500	0.680	0.820	Pep9hSC1	0.667
0.530	0.560	0.770	Pep9hSC10	0.620
0.500	0.580	0.720	Pep9hSC8	0.600
0.430	0.510	0.700	Pep9hSC7	0.547
0.260	0.310	0.420	Pep9hSC11	0.330
0.230	0.320	0.440	Pep9hSC12	0.330
0.310	0.310	0.360	Pep9hSC6	0.327
0.230	0.250	0.340	Pep9hSC3	0.273
0.120	0.160	0.240	Pep9hSC4	0.173

cDNA_Fibroblasts

F-Donor#10	F-Donor#6	F-Donor#5	Variant	Average
23.200	17.700	10.630	AAV-KP1	17.177
13.340	13.740	20.100	AAV-7m8	15.727
7.650	6.840	6.570	AAV-KP3	7.020
8.040	6.800	4.890	AAV-DJ	6.577
5.400	5.970	7.650	Shuffle32	6.340
4.970	5.900	7.150	Shuffle35	6.007
1.830	4.280	5.700	Shuffle31	3.937
3.280	3.590	4.460	Shuffle9	3.777
2.630	3.080	4.610	AAV2	3.440
2.700	3.320	3.940	AAV-HRP5	3.320
2.370	3.020	4.430	AAV-NP6	3.273
2.160	2.610	3.820	AAV-HRS1	2.863
2.100	2.620	3.630	AAV-NP94	2.783
2.930	2.620	2.250	Pep2hSC5	2.600
3.120	3.770	0.660	Pep2hSC1	2.517
2.830	2.530	1.820	AAV-NP22	2.393
1.830	1.510	1.370	Pep2hSC7	1.570
1.870	1.450	1.240	Pep2hSC8	1.520
1.300	1.390	1.130	Pep2hSC6	1.273
1.000	0.930	1.140	Pep2hSC3	1.023
0.970	1.030	1.010	Pep2hSC4	1.003
1.120	1.330	0.210	Pep2hSC2	0.887
0.640	0.900	0.170	Pep9hSC5	0.570
0.500	0.550	0.530	Pep2hSC9	0.527
0.450	0.590	0.110	Pep9hSC9	0.383
0.320	0.350	0.140	Pep2hSC10	0.270
0.290	0.330	0.140	Pep9hSC1	0.253
0.220	0.250	0.100	Pep9hSC2	0.190
0.160	0.170	0.060	Pep9hSC6	0.130
0.150	0.160	0.070	Pep9hSC7	0.127
0.150	0.130	0.050	Pep9hSC11	0.110
0.130	0.150	0.050	Pep9hSC8	0.110
0.110	0.140	0.040	Pep9hSC10	0.097
0.090	0.100	0.030	Pep9hSC12	0.073
0.080	0.090	0.050	Pep9hSC3	0.073
0.050	0.060	0.030	Pep9hSC4	0.047

Highest

Lowest

Figure S2. Performance of selected AAV variants in primary hSCs and in fibroblasts. Heat maps show the performance of selected variants. Percentage of NGS reads mapped to each AAV capsid (average of n = 2 barcodes/capsid) in seven primary SCs cultures and in three fibroblast cultures at both the level of DNA (cell entry, physical transduction) and mRNA (expression, functional transduction). Data is normalized to the 'pre-mix pool'.

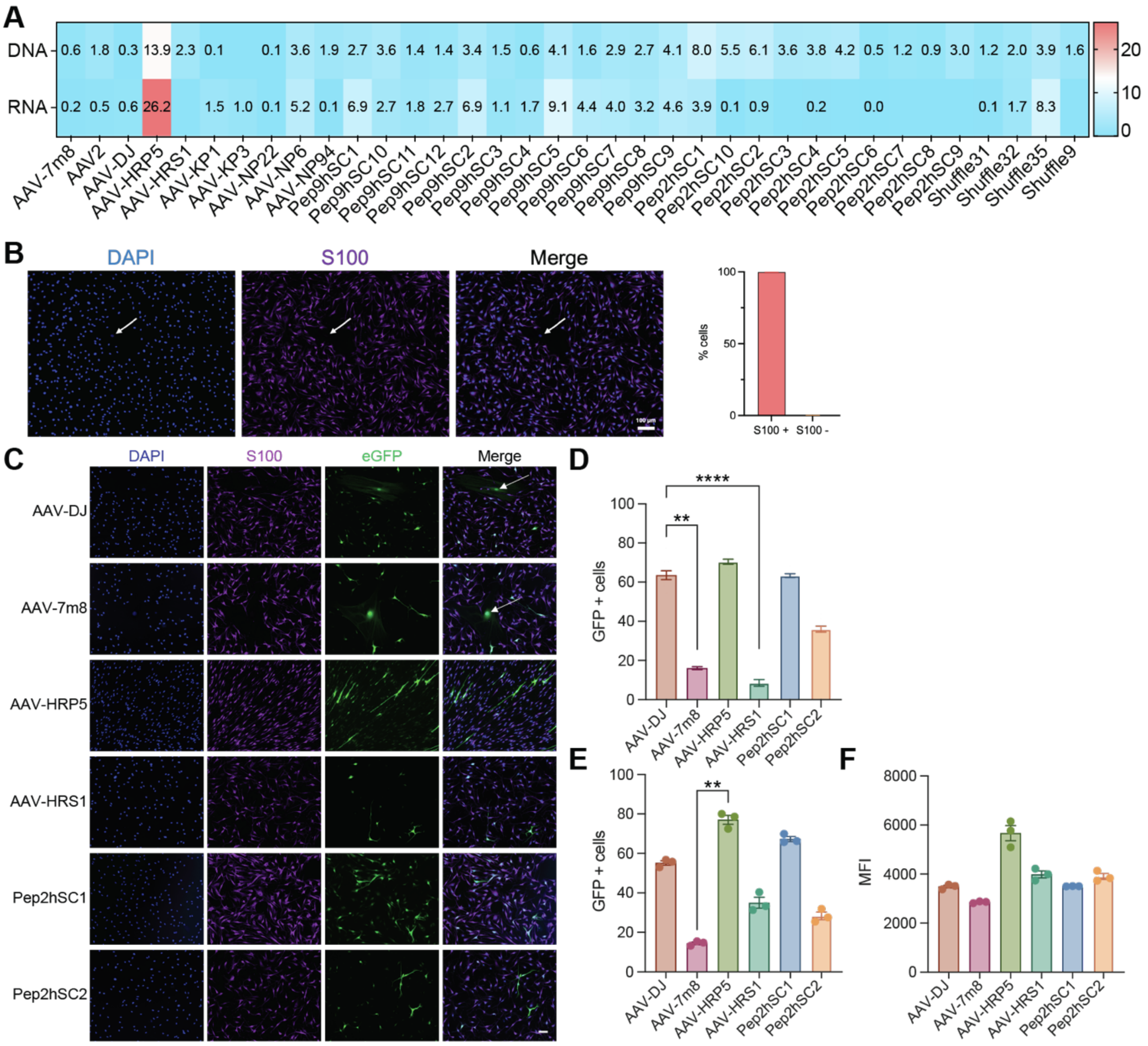

Figure S3. Assessment of AAV transduction on rat SCs.
(A) Analysis of barcoded variants with capsid recovery achieved at the level of both cell entry (DNA) and transgene expression (mRNA). Heat-map indicates the performance of each capsid as a percentage of total NGS reads for each cell type. Values are normalized to 'pre-mix pool' and are the average of 2 barcodes. (B and C) Rat SCs were transduced with the indicated variants (1,000 vg/cell) and analyzed by flow cytometry. (D) Characterization of the primary rat SC cultures derived from rat nerve segment. Representative images of rat and SC cultures stained with DAPI (blue) and the SCs marker S100 (purple). Quantification of the S100+ and S100- cells in three independent cultures. (E) Representative images of pure cultured rat SCs transduced with eGFP reporter AAVs packaged using indicated capsids (1,000 vg/cell). Blue: DAPI, purple: S100 (SCs marker), green: AAV-encoded GFP. Scale bar: 50 µm. (F) Percentage of eGFP+/S100+ cells. Quantification was performed from ≥ 10 images per variants with ≥ 80 cells counted per image. P values were determined by one-way ANOVA non-parametric Kruskal-Wallis test with Dunn's multiple comparison test (**p≤0.01; ****p≤0.0001). Data is represented as mean ± SEM.

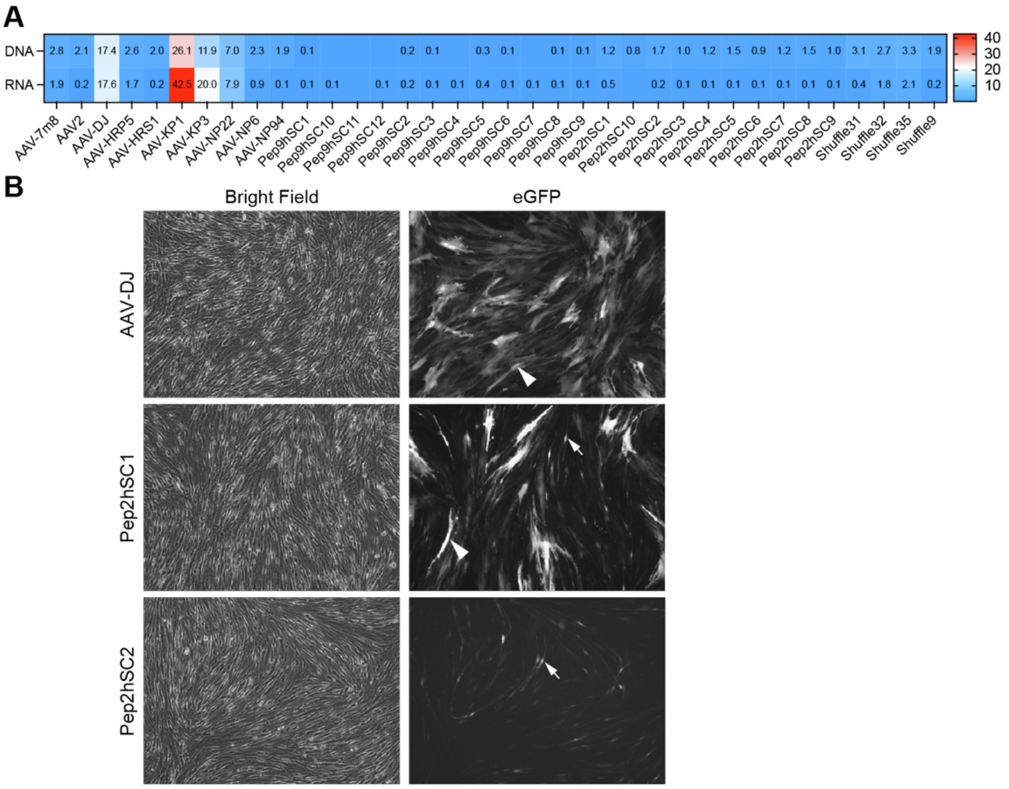

Figure S4. Assessment of AAV transduction on mouse SCs.

(A) Analysis of barcoded variants with capsid recovery achieved at the level of both cell entry (DNA) and transgene expression (mRNA). Heat-map indicates the performance of each capsid as a percentage of total NGS reads for each cell type. Values were normalized to 'pre-mix pool' and are the average of 2 barcodes. (B) Bright-field and fluorescence images 48 hours after AAV-GFP transduction of mouse SCs. Examples of transduced fibroblasts (arrowheads) and mouse SCs (arrows) are shown.

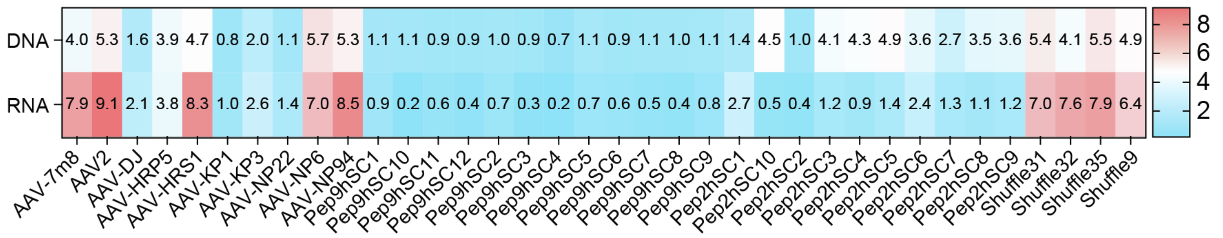

Figure S5. Assessment of AAV transduction on immortalized hSCs.

Analysis of barcoded variants with capsid recovery achieved at the level of both cell entry (DNA) and transgene expression (mRNA). Heat-map indicates the performance of each capsid as a percentage of total NGS reads from two transductions. Values were normalized to the 'pre-mix pool' and are the average of 2 barcodes.

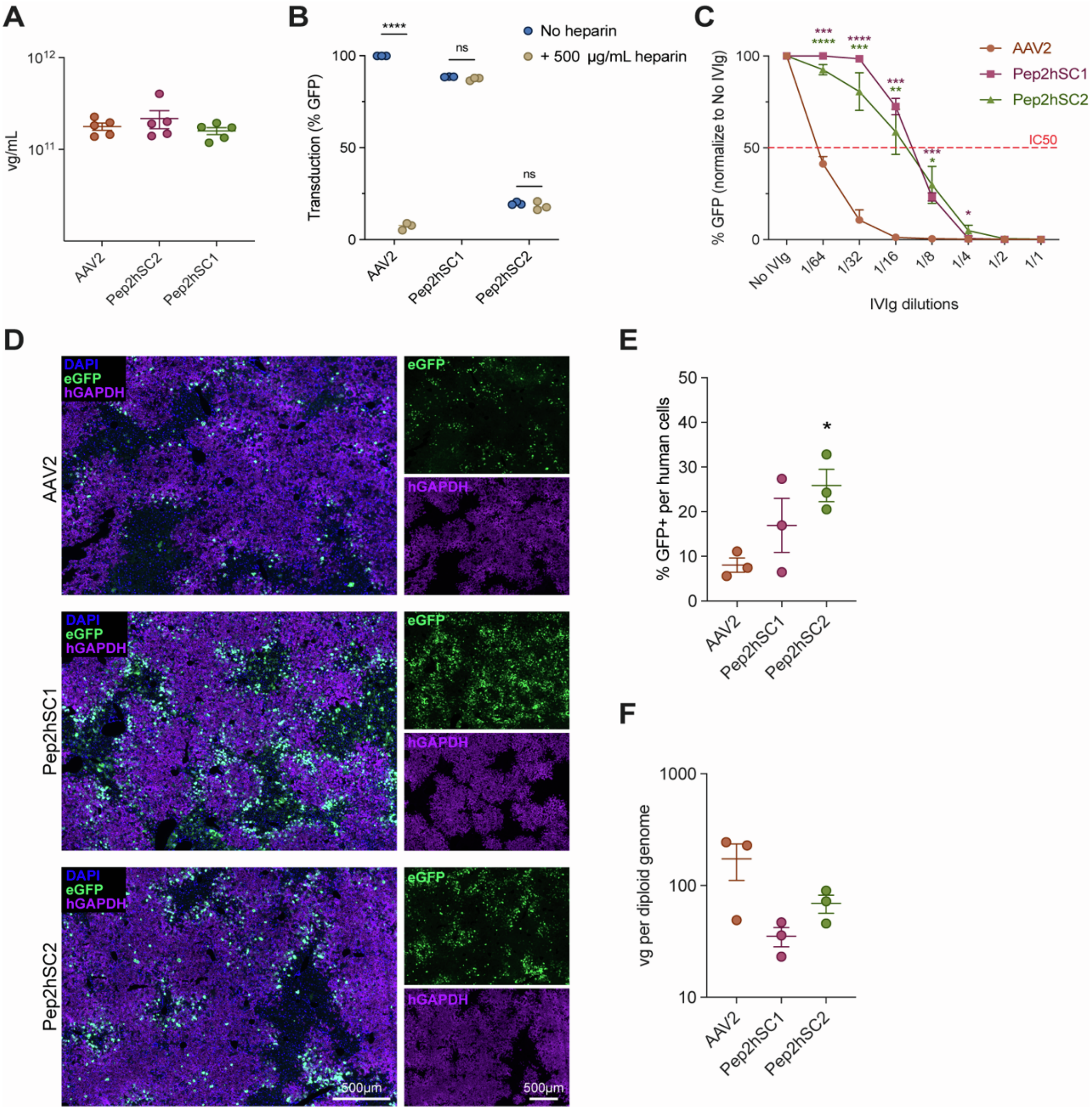

Figure S6. Characterisation of novel AAV capsid variants.

(A) Comparison of recombinant AAV crude production. Data are presented as mean ± SEM (n = 5 independent single dish lysate). (B) Heparin competition assay. AAV vector variants and the parental AAV2 were pre-incubated with heparin or not. Transduction efficiency on HEK293T cells was quantified by FACS according to eGFP positive cells 72h after transduction (n = 3 independent experiments). Data are represented as means ± SEM. Statistical significance was calculated using the two-tailed Mann-Whitney test by comparing vector performance with and without the presence of soluble heparin (****$p \leq 0.0001$; n.s., $p > 0.05$). (C) Neutralization assay of indicated AAV vectors following pre-incubation with human IVIg prior to transduction of HEK293T cells. The percentage of eGFP-positive cells 72h after transduction was analyzed by flow cytometry (n = 4 independent experiments). The dotted red line represents IVIg-mediated inhibition of AAV transduction by 50%. P values were determined by ordinary two-way ANOVA using Śidák's multiple comparison test (*$p \leq 0.05$; **$p \leq 0.01$, ***$p \leq 0.001$; ****$p \leq 0.0001$). Data is shown as mean ± SEM. (D-F) Evaluation of novel AAV variants in the humanized FRG (hFRG) model. (D) Representative images of hFRG mice liver tissue following I.V. injection with either AAV-DJ, Pep2hSC1 or Pep2hSC2 (dose = 2×10^{11} vg; n = 3 per variant). Human hepatocytes (purple), vector-expressed eGFP (green), DAPI (blue). Scale bar: 500µm. (E) Quantification of the percentage of transduced

human hepatocytes, individual data points represent the average of 11-15 human clusters per mouse. (F) AAV vector genomes per diploid cell in human hepatocyte cells. (F) Quantification of (D) the percentage of transduced human hepatocytes, Individual data points represent the average of 11-15 human clusters per mouse. Data are represented as mean ± SEM.

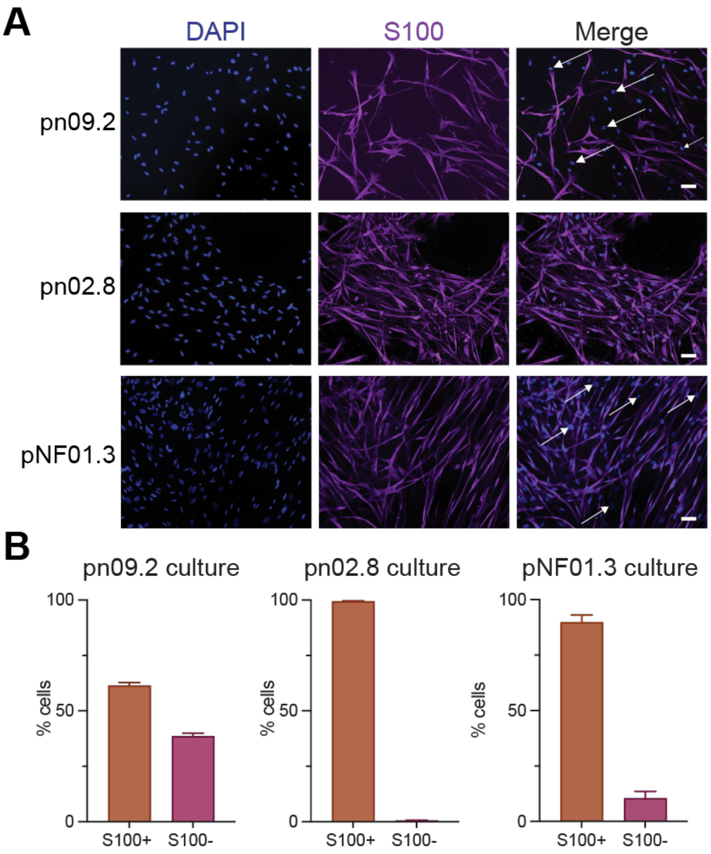

Figure S7. Characterisation of the primary human SC cultures.
(A) Representative images of human pn09.2, pn02.8 and pNF01.3 SC cultures stained with DAPI (blue) and the SCs marker S100 (purple). Arrows indicate cells that are S100-. Scale bar, 50µm. (B) Quantification of the S100+ and S100- cells in three independent cultures shows that pn09.2 culture contained both population with on average 60% of S100 positive and 40% of S100 negative cells, pNF03.1 culture contained both population with on average 90% of S100 positive and 10% of S100 negative cells while pn02.8 culture contained only S100 positive cells.

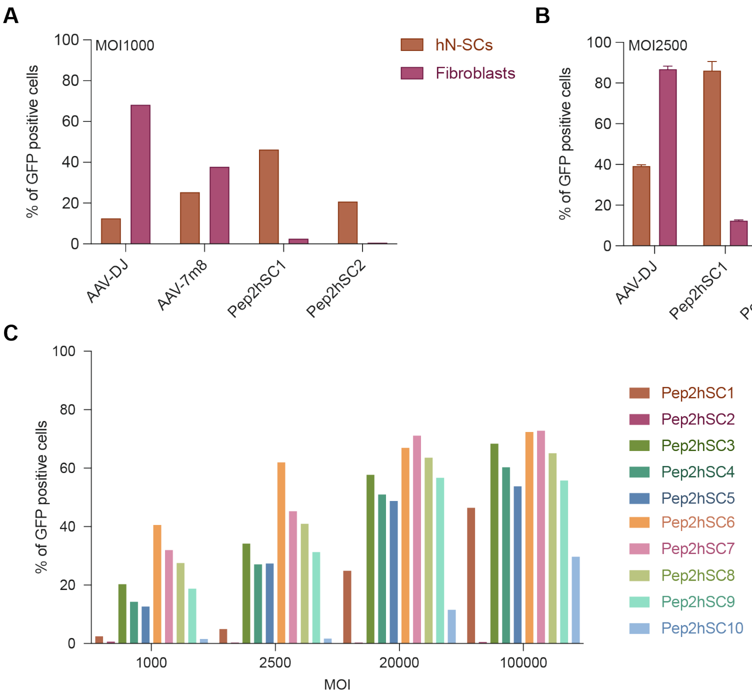

Figure S8. Transduction efficiency of selected variants for primary human SCs and fibroblasts.

(A-C) hN-SCs or fibroblast cells were transduced with vectors encoding a CMV-eGFP transgene at the doses indicated. 3 days post transduction cells were analysed by flow cytometry according to eGFP expression.

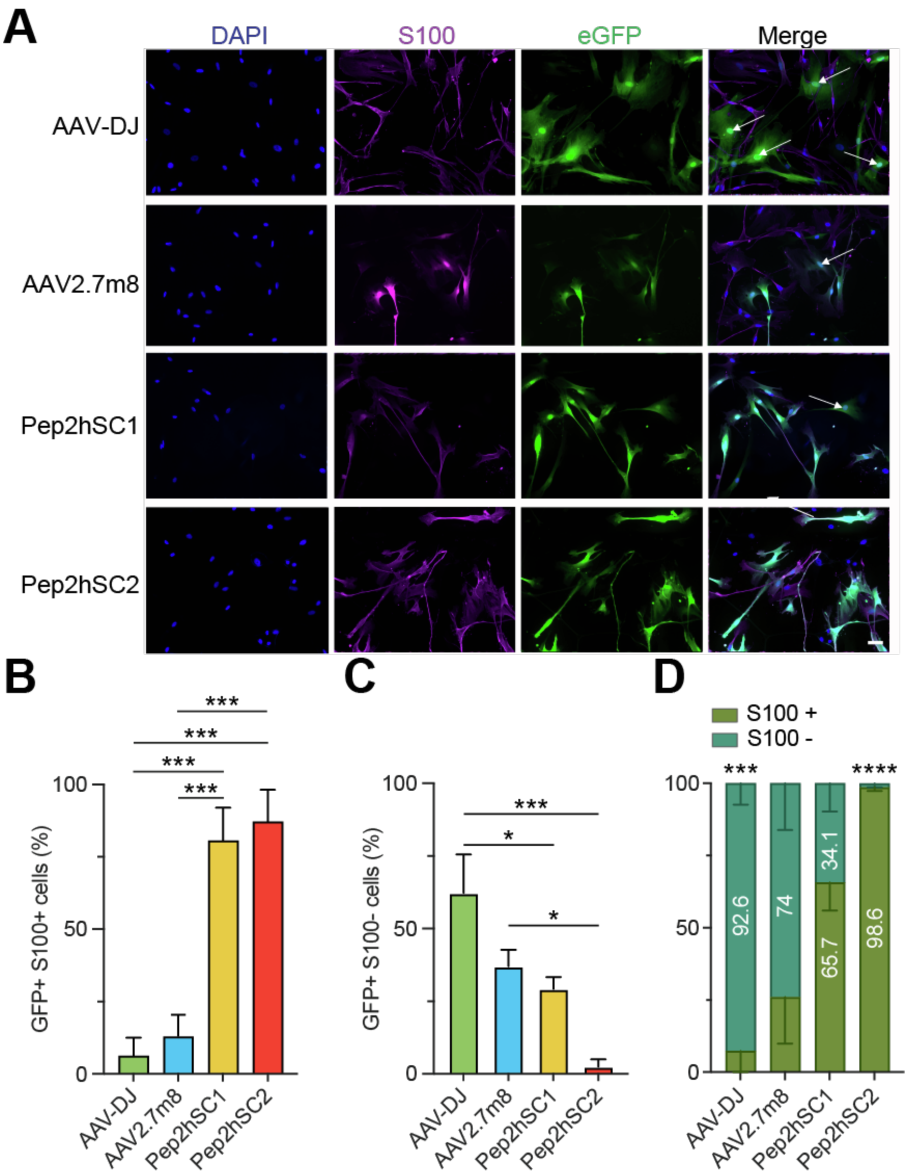

Figure S9. Transduction of pn09.2 SC culture with top AAV variants at MOT 10,000. (A) Representative immunofluorescence images of mixed cultured hSCs transduced with indicated AAV variants encoding an eGFP reporter (10,000 vg/cell). Arrows indicate eGFP+/S100- cells. Scale bar, 50µm. (B and C) Percentage of (B) eGFP+/S100+ cells, (C) eGFP+/S100- cells. Quantification was performed using ≥ 30 cells per image, and ≥ 4 images per variant. P values were determined by one-way ANOVA with Holm-Śidák's multiple comparison test (*p≤0.05; ***p≤0.001). Data are given as mean ± SEM. (D) Proportion of eGFP+ cells in the mixed hSC culture. Percentages of S100+ and S100- cells among total eGFP+ cells were calculated. P values were determined by unpaired t-test (***p≤0.001; ****p≤0.0001).

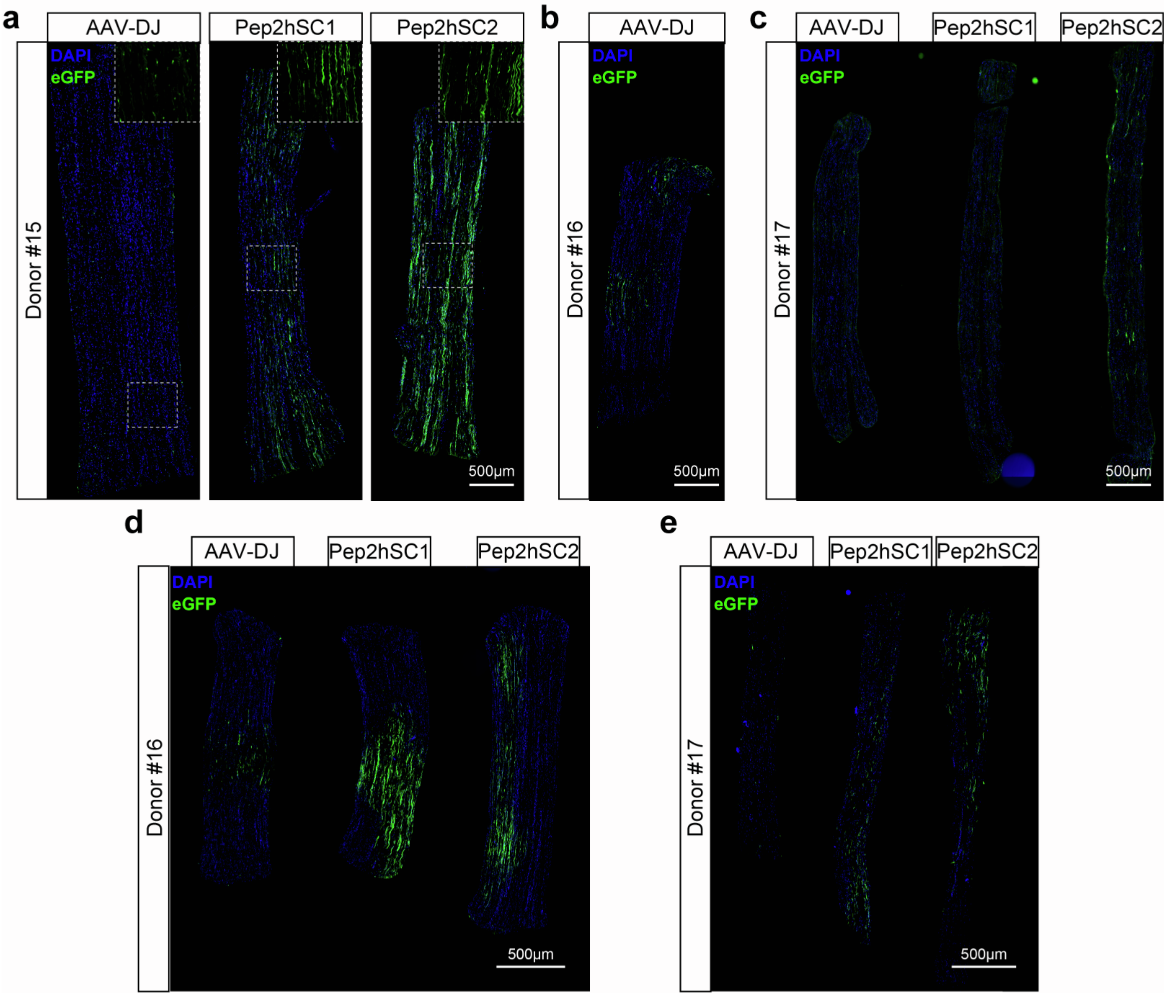

Figure S10. eGFP expression in human nerve segments.
Immunofluorescence of longitudinal sections of human sural nerve segments at (A-C) 7 days post injection and (D and E) 14 days post injection. Nerve segments (0.5cm) were injected with AAV-DJ, Pep2hSC1 or Pep2hSC2 vectors encoding a CMV-eGFP transgene (1×10^{10} vg dose per segment). DAPI (blue), AAV-encoded eGFP (green). Scale bar: 500µm.
Donor#15: 42-year-old male; Donor#16: 44-year-old male, Caucasian; Donor#17: 73-year-old male, Asian.

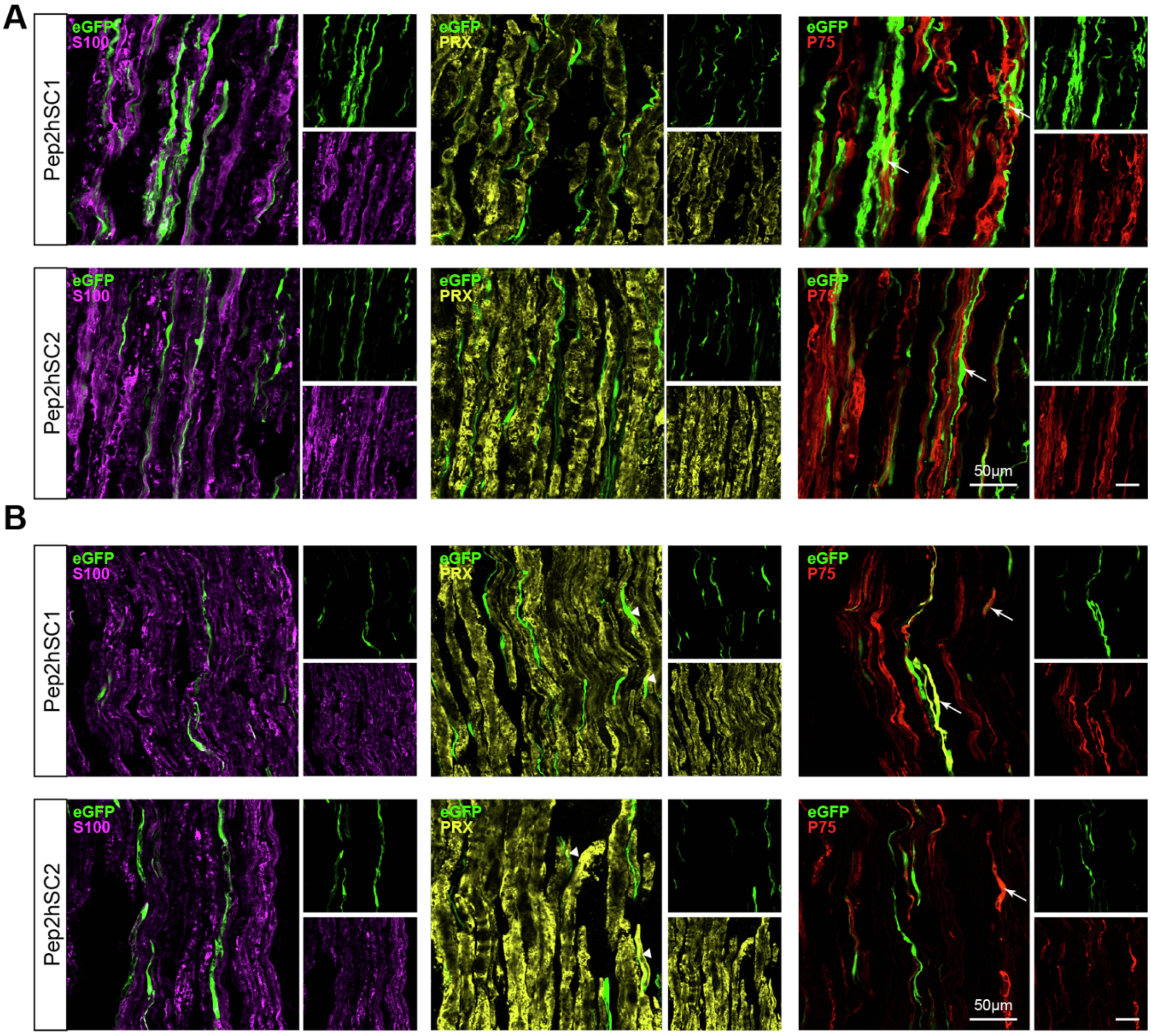

Figure S11. Immunolabeling for myelinating and non-myelinating SCs.
(A and B) Confocal microscopy images showing double immunostaining for eGFP and various markers on longitudinal sections (A) for Donor#16 and (B) for Donor#17. The sections were stained with either S100 (purple) for SCs, PRX (yellow) for myelinating SCs, or P75 (red) for non-myelinating SCs. Arrowheads in the right panel images show colocalization with non-myelinating SC marker P75. Donor#17 had some eGFP+ and PRX positive cells (arrows). Scale bar: 50µm.
Donor#16: 44-year-old male, Caucasian; Donor#17: 73-year-old male, Asian.

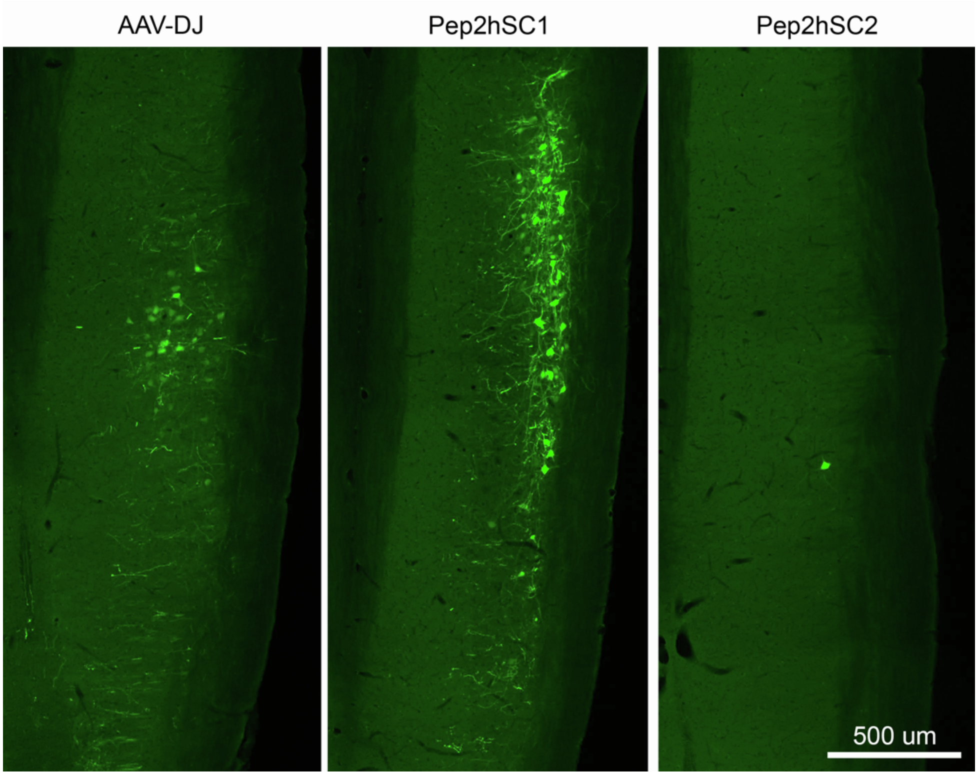

Figure S12. eGFP expression in spinal cord of mice administered by intraneural injection. Representative images of longitudinal spinal cord sections of mice following intraneural injection with either AAV-DJ, Pep2hSC1 or Pep2hSC2 (dose = 2×10^{10} vg; n = 1 per variant). Scale bar: 500µm.

Table S1. Information of donors used in studies conducted in primary hSC and fibroblast cultures and with *ex vivo* nerve explants.

Cell/Donor ID	Skin/Nerve/Fibroblast	Race/ethnicity	Age	Sex	Sources
pn02.8	Nerve	/	55	M	/
pNF01.3	Nerve tumor	/	/	/	Biopsy
pn09.2	Nerve	/	/	/	/
Donor#1	Nerve	Caucasian	28	M	Autopsy
Donor#2	Nerve	Asian	47	M	Autopsy
Donor#3	Nerve	Caucasian	58	M	Autopsy
Donor#4	Nerve	Caucasian	62	M	Autopsy
Donor#5	Fibroblast	Caucasian	50	F	Biopsy
Donor#6	Fibroblast	Caucasian	60	M	Biopsy
Donor#7	Skin	Caucasian	51	F	Biopsy
Donor#8	Skin	Caucasian	56	F	Biopsy
Donor#9	Fibroblast	Caucasian	63	M	Autopsy
Donor#10	Fibroblast	Caucasian	48	F	Biopsy
Donor#11	Skin	Caucasian	26	M*	Biopsy
Donor#12	Skin	Caucasian	64	F	Biopsy
Donor#13	Skin	Caucasian	49	F	Biopsy
Donor#14	Nerve	Caucasian	66	M	Autopsy
Donor#15	Nerve	/	42	M	Autopsy
Donor#16	Nerve	Caucasian	44	M	Autopsy
Donor#17	Nerve	Asian	73	M	Autopsy

Table S2. Information of primary human hepatocyte donors used to engraft FRG mice.

Sex	Internal ID	Albumin level [mg/ml]	Experiment	Cell origin	Replacement Index
Female	311	16.207	AAV2_2e11vg	Lonza #HUM181971	90.7%
Female	323	14.188	AAV2_2e11vg	Lonza #HUM181971	85.5%
Female	70	16.446	AAV2_2e11vg	Lonza #HUM181971	81.0%
Female	548	10.506	Pep2hSC1_2e11vg	Lonza #HUM181141	78.4%
Female	392	15.481	Pep2hSC1_2e11vg	Lonza #HUM181141	88.6%
Female	434	14.224	Pep2hSC1_2e11vg	Lonza #HUM181971	84.2%
Female	589	13.72	Pep2hSC2_2e11vg	Lonza #HUM181141	37.3%
Female	438	17.057	Pep2hSC2_2e11vg	Lonza #HUM181971	81.6%
Female	469	15.331	Pep2hSC2_2e11vg	Lonza #HUM181971	89.1%

Table S3. Oligonucleotide sequences.

Primer Name	Sequence (5'->3')
lco9_NNK7	CCATCCGGTTTGGGCCGCCTBMNNMNNMNNMNNMNNMNNMNNMCTCTGGCCCTGGTGGTTGGT
lco9-dsSyn	ACCAACCACCAGGGCCAGAG
F-cap-recovery	CCCTGCAGACAATGCGAGAGAATGAATCAGAATTCAAATATCTGC
R-cap-recovery	ATGCATATGGAAACTAGATAAGAAAGAAATACG
External_Seq_F	TGTGGATTTGGATGACTGC
External_Seq_R	GACCAAAGTTCAACTGAAACG
internal_cap_Seq	GTTCTTGATGAGAATCTGTGGAGGAGG
EGFP-F	TCAAGATCCGCCACAACATC
EGFP-R	TTCTCGTTGGGGTCTTTGCT
WPRE_R	GGATTTATACAAGGAGGAGAAAATGAAAG
lco2/lco9-NGS_R	CGGTTTATTGATTAACAGGCAATTACAGATTACG
lco2_PepLib_F	RCCACTAACCCTGTGGCC
lco9_PepLib_F	AACGAAGAGGAAATTAAAACCACTAACC
PepLib-R	CGTCTCTGTCTTGCCACACC
BC_F01	GTTCAGCTGGAGTTCGTGACCGCCG
BC_F02	GTCATGCTGGAGTTCGTGACCGCCG
BC_F03	CTGTAGCTGGAGTTCGTGACCGCCG
BC_F04	GTATTGCTGGAGTTCGTGACCGCCG
BC_F05	CTAGTGCTGGAGTTCGTGACCGCCG
BC_R	CAACATAGTTAAGAATACCAGTCAATCTTTCACAAATTTTGTAATCCAGAGG
hALB_F	TGCTGTCATCTCTTGTGGGCTG
hALB_R	AACTCATGGGAGCTGCTGGTTC
Pep2hSC1-F	GATTCGTGGTCTGAGGCGGCCGCGACCGCA
Pep2hSC1-R	ACCCCGCTTCCTCTGGCCCTGGAGGTTGGT
Pep2hSC2-F	GTTGATTTTGATAAGGCGGCCGCGACCGCA
Pep2hSC2-R	ATCCTTCCTCCTCTGGCCCTGGAGGTTGGT
